# Macroecological laws describe variation and diversity in microbial communities

**DOI:** 10.1038/s41467-020-18529-y

**Published:** 2020-09-21

**Authors:** Jacopo Grilli

**Affiliations:** 1 The Abdus Salam International Centre for Theoretical Physics (ICTP), Trieste, 34151 Italy; 2 Santa Fe Institute, Santa Fe, NM 87501 USA

**Keywords:** Biodiversity, Ecological modelling, Macroecology, Community ecology, Microbial ecology

## Abstract

How the coexistence of many species is maintained is a fundamental and unresolved question in ecology. Coexistence is a puzzle because we lack a mechanistic understanding of the variation in species presence and abundance. Whether variation in ecological communities is driven by deterministic or random processes is one of the most controversial issues in ecology. Here, I study the variation of species presence and abundance in microbial communities from a macroecological standpoint. I identify three macroecological laws that quantitatively characterize the fluctuation of species abundance across communities and over time. Using these three laws, one can predict species’ presence and absence, diversity, and commonly studied macroecological patterns. I show that a mathematical model based on environmental stochasticity, the stochastic logistic model, quantitatively predicts the three macroecological laws, as well as non-stationary properties of community dynamics.

## Abstract (web summary)

Microbes interact in different ways than macro-organisms, but their interactions can still form the basis for broader macroecological patterns like the Species Abundance Distribution. Here, the author shows that thre general ecological patterns can be found in microbes, within and across biome types.

## Introduction

No two ecological communities are alike, as species composition and abundance vary widely. Surveys of microbial communities, mapping taxonomy from Arctic oceans to zebras’ guts, have shown the incredible diversity of these ecosystems.

Often, we have a detailed understanding of which environmental factors affect community variability^1–4^ and, sometimes, the genetic drivers determining the response to different environmental conditions^5,6^. This qualitative understanding of the correlates, and potential causes, of the observed variation does not parallel with a mechanistic understanding of its fundamental and general properties^7–9^.

Recent experiments allowed to document the existence and quantify the effect of several ecological mechanisms driving diversity in vitro^10–14^. Sometimes, with counter-intuitive results. For instance, many species can coexist on a single supplied resource thanks to widespread cross-feeding^13^. Environmental modification can lead to ecological suicide when one species, in the absence of other ones, modify pH to such a degree that lead to extinction of the whole population^14^. These growing body of fundamental results in microbial ecology are made possible by the simplified nature of the experimental communities, which typically consist of an handful of interacting species. It is challenging to upscale the experimental setups to match the complex spatio-temporal conditions of natural communities, in order to characterize the processes shaping the variation of many coexisting species.

Environmental fluctuations, competition, cross-feeding, environmental modification, demographic stochasticity, migration, and many other ecological forces shape microbial communities over time and space. The existence of such forces is not in doubt. Their quantitative strength and relative relevance in determining composition and variation in natural communities are unknown. It is in fact extremely challenging to disentangle the effect of multiple mechanisms in communities with thousands of species interacting. In such complex communities, mechanisms and microscopic forces manifest in emergent, macroscopic, properties. Macroecology, the study of ecological communities through patterns of abundance, diversity, and distribution^15^, is therefore a promising approach to study quantitatively variation in microbial communities^16–18^, and to provide quantification of mechanisms that are shaping them.

The most studied pattern in (macro)ecology is the species abundance distribution (SAD)^19,20^, which is defined as the fraction of species with a given abundance. Multiple functional forms, and consequently multiple mechanisms, have been proposed to describe the empirical SAD in microbial communities^17^. While SADs are highly studied and characterized, it is often neglected that three distinct and independent sources of variation influence their shape: sampling noise, fluctuation of abundances of individual species, and variability in abundance across species. This work disentangles these sources of variation in three macroecological laws.

Here, I show that three macroecological laws describe the fluctuations of abundance and diversity. These three ecological laws hold across biomes and for both cross-sectional and temporal data, and are fundamental, as they suffice to predict, without fitting any additional parameters, the scaling of diversity and other commonly studied macroecological patterns, such as the SAD. These laws allow to generate in silico ecological communities, providing a statistically sophisticated ground truth, that allows to test ecological theories, models, and mechanisms.

Macroecological patterns are the bridges from uncharacterized variation to ecological processes and mechanisms. I show that the stochastic logistic growth model, which is based on environmental stochasticity, reproduces the three macroecological laws, as well as dynamic patterns in temporal data. Both data and model show that, at the taxonomic resolution commonly used, competitive exclusion is rare and variation of species presence and abundance is mostly due to environmental fluctuations.

## Results

### Abundance fluctuations are gamma distributed

**Fig. 1:**
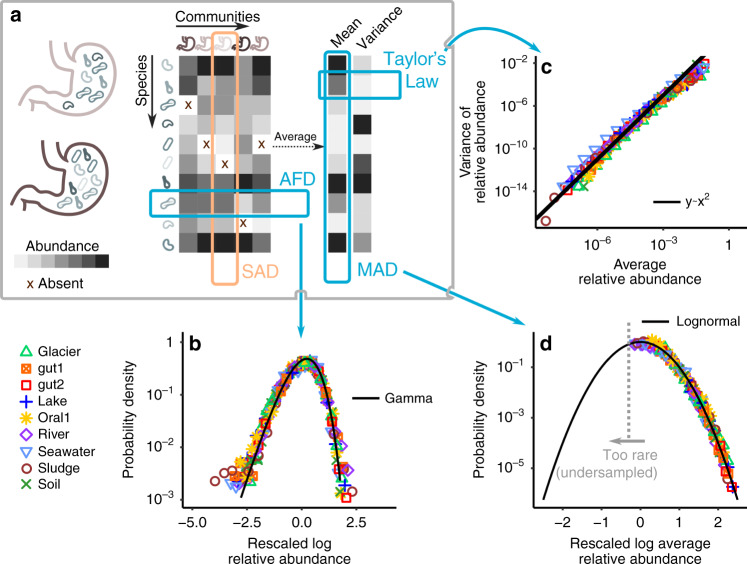
Laws of variation in microbial communities. **a** The species abundance distribution (SAD) describes the fluctuations of abundance across species in a community. **b** The Abundance Fluctuation Distribution (AFD) describes the distribution of abundances of a species across communities. I consider cross-sectional data from 9 data sets (colored symbols, see Methods). A Gamma distribution (solid black line) closely matches the AFD, here reported for the most abundant species (see Methods). The Gamma distribution describes the AFD of both abundant and rare species (Supplementary Note 1 and Supplementary Fig. 2). **c** The mean and variance of the abundance distribution are not independent across species, a relationship known as Taylor’s Law. The variance is, in fact, proportional to the square of the mean (solid line), implying that the coefficient of variation of the abundance fluctuations is constant across species (Supplementary Fig. 7). Taylor’s Law (together with a Gamma AFD) implies that a single parameter per species (the average abundance) recapitulates the distribution of fluctuations. **d** The Mean Abundance Distribution (MAD), defined as the distribution of mean abundance (obtained by averaging over communities) across species, is Lognormally distributed (black line, Supplementary Note 7).

The first pattern I consider is the abundance fluctuation distribution (AFD), which is defined as the distribution of abundances of a species across communities (Fig. 1a).

This quantity is strongly influenced by sampling errors, especially when, because of fluctuations, a species becomes rare. For the most abundant species, these sampling errors can be neglected and Fig. 1b shows that the Gamma distribution, with species’ dependent parameters, well describes the AFD across biomes for the most occurrent species (Supplementary Fig. 1). In the Methods section, I introduce a method, based on the moment generating function, to remove sampling effects and infer the original distribution of abundance fluctuations. Supplementary Fig. 2 shows that the abundance fluctuations of rarer species are also Gamma distributed, independently of their presence and typical abundance. The probability that species *i* has abundance *x* in a given community is therefore

1 $${\rho }_{i}(x)=\frac{1}{\Gamma ({\beta }_{i})}{\left(\frac{{\beta }_{i}}{{\bar{x}}_{i}}\right)}^{{\beta }_{i}}{x}^{{\beta }_{i}-1}\exp \left(-{\beta }_{i}\frac{x}{{\bar{x}}_{i}}\right)\,.$$

The two parameters ${\bar{x}}_{i}$ and *β*_*i*_ fully characterize the AFD of each species. The parameter *β*_*i*_ is the squared inverse coefficient of variation: ${\beta }_{i}={\bar{x}}_{i}^{2}/{\sigma }_{{x}_{i}}^{2}$, where ${\bar{x}}_{i}$ is the average abundance of species *i* and ${\sigma }_{{x}_{i}}$ is its standard deviation.

This law was tested against two alternative distributions (Lognormal in Supplementary Fig. 5 and zero-inflated Gamma in Supplementary Fig. 6), obtaining a superior performance of the Gamma distribution in all the data sets considered in this study. Whichever ecological processes are at the origin of species’ abundance variation, they manifest regularly and consistently in a Gamma AFD.

### Abundance predicts presence

**Fig. 2:**
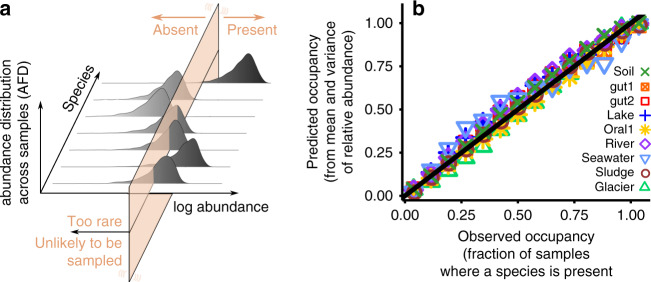
The AFD predicts the presence/absence of species from fluctuations of abundance. **a** Relationship between fluctuation in abundance and the absence of species. The fluctuations of species abundances across communities (AFD) are Gamma distributed (Fig. 1), which implies that species are absent only because of finite sampling. **b** Tests the prediction, by comparing the occupancy of species (the fraction of communities where a species is presence) in different biomes with what expected from independent sampling from Gamma distributed relative abundances (Supplementary Note 4 and Supplementary Fig. 3).

The probability that a Gamma-distributed variable is exactly equal to zero vanishes. A direct consequence of the first macroecological law (a Gamma AFD) is that all instances in which a species is absent should be imputed to sampling error. This surprising prediction is directly tested in two ways. If the absence is caused by sampling error, one can predict the occupancy of a species, defined as the fraction of communities where it is present, from the AFD. Assuming a Gamma AFD, the expected occupancy of species *i* is given by (see Methods and Supplementary Note 4 for the full derivation)

2 $$\langle {o}_{i}\rangle =1-\frac{1}{T}\sum_{s = 1}^{T}{\left(1+\frac{{\bar{x}}_{i}{N}_{s}}{{\beta }_{i}}\right)}^{-{\beta }_{i}}\,,$$

where *N*_*s*_ is the total number of reads in sample *s* and *T* is the total number of samples. Since absence is predicted to be due to sampling errors, as sampling error reduces (i.e., when the total number of reads *N*_*s*_ increases) occupancy is predicted to tend to 1. Figure 2 shows that Eq. (2) predicts the occupancy from the first two moments of species abundance fluctuations (Supplementary Fig. 3). Note that the fact that a Gamma AFD reproduces this pattern is also an indirect test of the hypothesis that the AFD is Gamma. Supplementary Fig. 4 shows that a Lognormal AFD fails in reproducing the observed occupancy.

Further evidence to the claim that most instances where a species is absent are due to sampling error is provided using Bayesian model selection. A Gamma AFD is compared with a zero-inflated Gamma distribution, which explicitly includes species absence. The Gamma AFD is statistically superior to the zero-inflated Gamma distribution (see Methods and Supplementary Fig. 6).

This result strongly suggests that, at the taxonomic resolution used in this study, competitive exclusion is absent or, at least, statistically irrelevant. Importantly, this result clarifies the relation between abundance and occupancy^21^, which has been reported in multiple microbial systems^18,22,23^ but has never been quantitatively characterized and explained.

### Taylor’s Law

The mean and variance of abundance fluctuations are sufficient to characterize the full distribution of abundances of species across communities, as Eq. (1) depends only on the two moments ${\bar{x}}_{i}$ and ${\sigma }_{{x}_{i}}$. The second macroecological law describes the relation between mean and variance of species abundance, which is often referred to as Taylor’s Law^24^. Taylor’s law has been reported in many contexts, ranging from ecology^25,26^ to physiology^27–29^, from economics^30^ to geomorphology^31^. Figure 1c shows that Taylor’s law holds for the composition of microbial communities. In particular, the variance scale quadratically with the mean, implying that the coefficient of variation of the abundance fluctuations is constant (with respect to mean abundance, see Methods and Supplementary Note 5). Thanks to Taylor’s Law, one needs therefore only one, instead of two, parameters per species—their average abundance—to describe species abundance fluctuations. In particular, it implies that *β*_*i*_ = *β* for all species.

It is known that a Taylor’s law with exponent 2 can arise as a consequence of sampling biases^32,33^. Average and variance can, in principle, be calculated over independent realizations of a process, over time, or over both. If the duration of observations is too large compared to the number of independent replicates, the empirically measured value of Taylor’s exponent converges to 2 independently of the true exponent^32^. Since the relationship between variance and mean was considered over the variation across communities, without any time dimension, this caveat does not apply to the results of Fig. 1. Another bias emerges when data are sampled in blocks from the same skewed distributions. Also in this case a Taylor’s law with exponent 2 emerges between sample mean and standard deviation^33^. The existence of a large variation (of several orders of magnitudes) of the sample average abundance suggests that the observed Taylor’s law reflects a true scaling of mean and variance between distribution, rather than a sampling effect. This is confirmed by the replicability of the average abundance: species’ average abundance strongly correlates in similar biomes across data sets (Supplementary Fig. 8). Both these important caveats, therefore, do not apply to the analysis presented above, suggesting that the exponent 2 reported in Fig. 1 corresponds to an actual property of the data.

### Average abundances are lognormally distributed

Since Taylor’s law holds, the average abundance alone characterizes the distribution of abundance fluctuations of each species. Supplementary Fig. 8 shows that the average species abundances have a reproducible dependence on the biome, implying that its variation across species and biomes carries important biological information.

The mean abundance distribution (MAD) describes how the average abundance is distributed across species. Figure 1d shows that the MAD is Lognormally distributed for all the data sets considered in this work (Supplementary Figs. 9 and 10): if a species is picked at random, the probabily of observing an average abundance $\bar{x}$ is

3 $$p(\bar{x})=\frac{1}{\sqrt{2\pi {\sigma }^{2}}\bar{x}}\exp \left(-\frac{{({\mathrm{log}}\,\bar{x}-\mu )}^{2}}{2{\sigma }^{2}}\right)\,.$$

The parameter *σ* characterizes the variability of the logarithm of the mean abundance across species. Since in a finite number of samples rare species are likely to be never sampled, the empirical MAD displays a lower cutoff which is determined by sampling. In fact, if a species is rare enough (i.e., if ${\bar{x}}_{i}<c$, where *c* is a cutoff determined by the number of samples and the total number of reads in each sample), it becomes extremely unlikely to observe it. If the “true” distribution of ${\bar{x}}_{i}$s is described by the probability distribution function $p(\bar{x})$, one expects to observe only the right part of the distribution, i.e.,

4 $${p}_{\mathrm{emp}}(\bar{x})=\frac{\theta (\bar{x}-c)p(\bar{x})}{\int\ \mathrm{d}z\theta (z-c)p(z)}\,,$$

where *θ*(*z* − *c*) is 1 if *z* > *c* and zero otherwise (see Supplementary Note 7 for details on parameter estimate). Note that, in reality, *c* is not a hard cut-off. In this context, it refers to the minimal average abundance above which the error on the mean abundance due to sampling is negligible.

Equation (4) allows to estimate the total diversity, under the assumption that Eq. (3) holds, i.e., that the MAD is lognormal also for the rarer species. I find that the total diversity is typically at least twice as large as the recorded one (Supplementart Table 2). A Lognormal MAD also rules out Neutral Theory^34,35^ as an explanation of community variability. Neutral Theory in fact assumes species’ symmetry^35^—the outcome is statistically invariant when exchanging species identities—which implies that average abundances (averaged over time or across replicates) are species independent. Averaging over an infinite number of replicates one would find that, in Neutral models, the averages abundances of different species converge to the same value, and the MAD to a Delta distribution. For a finite number of independent samples, one would observe a Gaussian MAD (Supplementary Note 11), which can be easily rejected from the data.

### Prediction of other macroecological patterns

The three laws presented so far—the Gamma AFD, Taylor’s Law with exponent 2 and the Lognormal MAD—can be fully parameterized for each biome knowing the first two moments *μ* and *σ* (Eq. (3)) of the MAD (how the mean relative abundance differs across species), the total diversity and the coefficient of variation of the AFD (what is the average variation of species’ abundance across communities, i.e., the intercept of Fig. 1c), which is related to *β* (Eq. (1)).

Knowing the three laws and their parameters, and assuming that species abundance fluctuations are independent, one can generate synthetic communities for arbitrary levels of sampling. Here I contrast these synthetic communities to the empirical ones, by comparing their statistical properties. In particular, I focus on commonly studied macroecological patterns (e.g., the SAD). The goal of this comparison is twofold. On the one hand, it allows to testing the realism of these synthetic communities, serving as a further indirect test of the correctness and the statistical relevance of the three macroecological laws. In fact, a strong mismatch between the properties of synthetic communities and the empirical ones would imply the existence of other strong statistical constraints that go beyond the three laws. For instance, if species abundance fluctuations were strongly correlated, one would expect to find a significant mismatch between observed SAD and the ones predicted assuming independence. On the other hand, it is also a test for the relevance of other macroecological patterns, given the knowledge of the three macroecological laws. For instance, one might wonder whether the shape of the SAD add information—that is not already encoded in the three macroecological laws—on the statistical properties of the community structure.

**Fig. 3:**
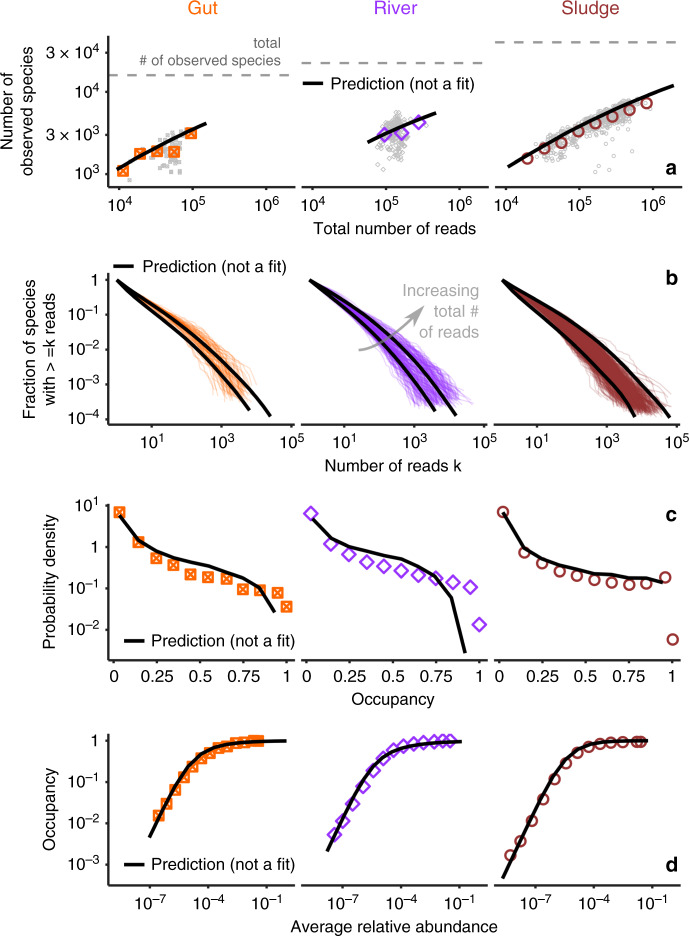
The AFD, Taylor’s Law and MAD quantitatively predict macroecological patterns. **a** Scaling of diversity (measured as the number of species) with the total number of reads (Supplementary Fig. 13). **b** Species abundance distribution (Supplementary Fig. 17). **c** Occupancy distribution (Supplementary Fig. 15). **d** Abundance-occupancy relation^21^ (Supplementary Fig. 16). Data are colored points/line. Predictions (black lines) are obtained from the macroecological laws without fitting any additional parameter. Gray points in (**a**) are individual communities (colored points are averages).

Four macroecological patters are considered: the relation between diversity and the number of sequences sampled^36^ (which parallels the Species-Area relationship)^16^, the SAD^19,20^, the occupancy distribution^37^ (the probability that a species is present in a given fraction of the samples) and the abundance-occupancy relationship^21^ (see Supplementary Note 8 for other quantities). It is important to note that these patterns are all affected by sampling, species abundance fluctuations, and species differences. Knowing the three macroecological laws allows to analytically calculate a prediction for these quantities (see Methods and Supplementary Note 8 for the analytical derivation). Figure 3 shows that the predictions of these macroecological patterns match the data accurately. The three laws do not only hold in general, being valid across biomes: they are also fundamental, as they suffice to predict other macroecological quantities.

### Macroecological laws hold for temporal data

**Fig. 4:**
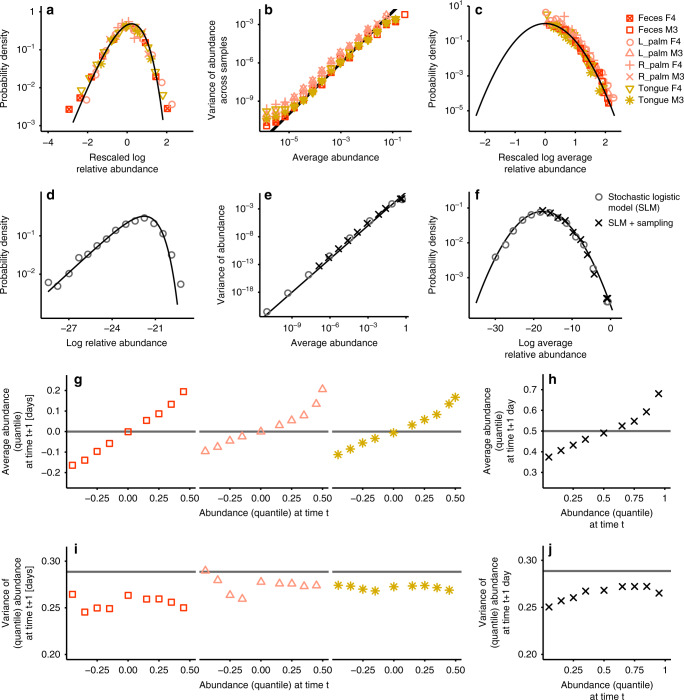
Macroecological laws hold for temporal data. **a**–**c** The same laws that describe presence and abundance variation across the community (black lines) also hold for time data (colored points, see Methods and Supplementary Note 9). **d**–**f** The stochastic logistic model (SLM) reproduces the empirically observed AFD, Taylor’s law and MAD, respectively. Gray circles are the results obtained with the SLM, and the black crosses the ones obtained using SLM together with sampling. **g** The average quantile abundance given an average quantile abundance in the previous day (averaged over species, see Methods). The gray solid line shows the expected relation in the absence of time dependence. **i** Similar to (**g**), the variance of the quantile abundance given an average quantile abundance in the previous day (averaged over species). **h**, **j** The SLM correctly predicts the non-stationary properties shown in (**g**) and (**i**) (see Methods).

A question that naturally arises is whether the success of the AFD, together with the other two macroecological laws, in predicting the scaling of abundance and diversity translates into an ecological prediction on the nature of stochasticity. Which ecological process is responsible for the fluctuations of species abundance across communities? The ability of a Gamma AFD in predicting occupancy from its first two central moments, as illustrated in Fig. 2, rules out mechanisms that explain variation as a consequence of alternative stable states driven by biotic or abiotic interactions. These mechanisms would correspond in fact to more complicated relationships between abundance and occupancy (Supplementary Note 11), that cannot be described by a Gamma AFD. An alternative is that the variation in abundances is the effect of a mechanism with some intrinsic variability. This variability could be due to heterogeneity (e.g., two communities are different because the environmental conditions were, are and will be different) or stochasticity (e.g., two communities are different because the environmental conditions are independently fluctuating over time). I tested these two scenarios using longitudinal (temporal) data (see Methods). In the former scenario, the three macroecological laws should differ between cross-sectional (i.e., across communities) and longitudinal (i.e., across time) studies. While in the latter case, they should also hold when a community is followed over time. Figure 4 shows that the three macroecological laws also hold for longitudinal data, suggesting that fluctuations in abundance are mainly due to temporal stochasticity (Supplementary Note 9). This result does not contradict the existence of replicable differences between communities (e.g., host genetics correlates with community composition of gut microbiome)^38^: most of the variation, and not all of it, is due to temporal stochasticity.

### The stochastic logistic model (SLM) reproduces macroecological laws

The observation that variation in abundances is mostly due to stochasticity over time, together with the three macroecological laws, strongly constrains the validity of models aiming at explaining and reproducing community dynamics. It is natural to interpret stochasticity as due to environmental fluctuations (an alternative would be demographic stochasticity, which is ruled out in Methods).

I considered the SLM to describe species population dynamics. The SLM assumes that species populations grow logistically, with a time-dependent growth rate, which fluctuates at a faster rate than the average growth rate (i.e., the timescale associated with growth-rate fluctuations is much shorter than the typical timescale of population dynamics). If *x*_*i*_ is the abundance of species *i*

5 $$\frac{{d}{x}_{i}}{{d}t}=\frac{{x}_{i}}{{\tau }_{i}}\left(1-\frac{{x}_{i}}{{K}_{i}}\right)+\sqrt{\frac{{\sigma }_{i}}{{\tau }_{i}}}{x}_{i}{\xi }_{i}(t)\,.$$

where the noise *ξ*(*t*) is assumed to have time correlation $\langle {\xi }_{i}(t){\xi }_{j}(t^{\prime} )\rangle ={\delta }_{ij}\delta (t-t^{\prime} )$. Taylor’s Law and the observed Lognormal MAD constraints the parameters value. Taylor’s Law requires *σ*_*i*_ = *σ* (independently of *i*, Supplementary Note 12), while the Lognormal MAD implies that the *K*_*i*_s are lognormally distributed across species. Figure 4 shows that the SLM reproduces the three macroecological laws at stationarity. In particular it predicts a Gamma AFD^39^ as observed in the data.

One important assumption of the SLM is that environmental noise is white, with autocorrelation time which is much shorter than the population dynamics timescale *τ*. It is known that environmental fluctuations are typically non-white^40,41^. Supplementary Note 13 extends the model to include non-white environmental noise, with finite autocorrelation time *τ*_*ϵ*_. Surprisingly, simulations and analytical calculations (see Supplementary Note 13, Supplementary Fig. 25 and Methods) show that the AFD is almost unaffected even when the noise correlation timescale and the population dynamics timescale overlap (i.e., when *τ*_*ϵ*_/*τ* ≈ 1).

The SLM assumes that species are not interacting and their populations change independently over time. As it is known that species interact, it is natural to ask under what conditions this is a useful approximation. It is known that the interacting models (e.g., the Lotka-Volterra system of equations) can reduce effectively to the SLM, when the number of species is large enough. Intuitively, the effect of all the other populations on a focal one can be effectively approximated as random noise when many species interact in a perturbed environment. It is possible to rigorously derive the SLM as an effective equation using tools from the statistical physics of disordered systems^42^.

A correct model describing population dynamics should not only reproduce the stationary distribution but also time-dependent quantities. The dynamics of the system can be fully characterized by the transition probability, which is defined as the probability of observing an abundance at time *t* + Δ*t*, conditioned to the abundance at time *t*. Figure 4 shows the first two central moments of this distribution (see Methods), for Δ*t* = 1 day.

An important observation is that one can detect a signature of dynamics: the longitudinal data, collected with a time-spacing of 1 day, display a non-trivial time correlation (with a typical relaxation time-scale equal to 19 hours, see Methods). This timescale might appear much longer than the typical duplication time of bacteria in standard experimental conditions. In drawing this comparison, it should however be considered that in nature resource are more limited and the environment more stressful than typical lab conditions^43^. Measuring doubling times in the wild is challenging, but existing estimates are consistent with the inferred relaxation timescale of about 19 h. For instance Gibson et al.^44^, by measuring the rate of mutation accumulations, estimate that *E. coli* doubles every 15 h in the wild as opposed to 20 min in its most favorable lab condition. Across species, doubling times are 2-fold to 50-fold longer in the wild than in the laboratory, consistently with our finding. Figure 4 shows the SLM reproduces also the dynamics patterns, giving further validation to the hypothesis that environmental fluctuations drive the variability observed in the data.

## Discussion

Here, I considered longitudinal and cross-sectional data of microbial communities from many different environments and studied their patterns of presence, abundance, and diversity with a macroecological perspective. Three general and fundamental laws emerge. These laws characterize quantitatively the abundance variability of individual species across space and time and the difference in typical abundance across species.

From a methodological standpoint, the characterization of these laws allows to formulate a data-driven null model that can be used to generate communities in silico. By exploring the statistical properties of synthetic communities, one can show that they match the empirical ones. This comparison is not just a statistical exercise and it has deep consequences on how these data should be used and interpreted. For instance, it is shown that abundance fluctuations and sampling effects alone can predict presence, implying that most of the instances where species are absent are due to sampling errors. These results raise concerns on the interpretation of presence–absence data, for instance used to define core microbiomes^45^ and of co-occurrence data^46^. More interestingly, these observations have deep implications on our understanding of the mechanisms shaping the composition of microbial communities. As true absence of species appears to be quite rare, limiting similarity and competitive exclusion must have a small role in determining inter-community variability.

Differences between in silico and in vivo communities also emerge and provide important insight on mechanisms. For instance, non-trivial spurious correlations between species abundance fluctuations emerge in in silico communities, mainly because of finite sampling (Supplementary Fig. 26 and Supplementary Note 14). The majority of species pairs have correlations compatible with what predicted by the null model, while only a small group is significantly correlated. The relative small, yet significant, degree of the deviation implies that microbial communities are in a weakly interacting (or weakly correlated) regime, where interactions are sparse and correlations are weak.

These results are contingent on the very definition of species and the taxonomic resolution used in this work (97% OTUs, Supplementary Note 1). While competition appears not to be a driver of correlated variation at this taxonomic resolution, it very likely becomes an important contributor at finer resolutions. On the other hand, the trophic structure of functional groups^47^ might be masked at the current resolution, and it might be revealed as variation is studied at a coarser taxonomic (or functional) scale. Whether the macroecological laws differ when the taxonomic resolution is changed is an open question. The possible dependence of macroecological patterns on the taxonomic scale is not a limitation, but is rather an asset. It would in fact correspond to a shift in importance between ecological processes that shape variation at different taxonomic scales.

One of the early critiques to macroecology is the lack of a direct connection between ecological mechanisms and patterns. For instance, the shape of the SAD is quite insensitive to the underlying variation of ecological forces^48,49^. This paper contributes in filling the gap between mechanistic models and macroscopic patterns, by disentangling different sources of variation of species abundance. We showed that the SLM describes both stationary patterns in static (cross-sectional) data and abundance dynamics in temporal (longitudinal) data. The model points to environmental variability as the main source of variation of presence and abundance in microbial communities.

These results parallel the ones found in non-microbial ecosystems, in tropical forests in particular. In those ecosystems, neutral theory has played an important role in predicting static^34,50,51^ and dynamic^52^ patterns of diversity. While the success of neutral theory in predicting static patterns, and the SAD, in particular, is well accepted, it has been increasingly recognized that neutral models fail in explaining temporal abundance dynamics^53,54^. Adding environmental noise to neutral models^55–57^, but still keeping a species-symmetric assumption^35^, allows to better explain the tempo and properties of abundance dynamics.

One important difference between the models proposed to explain the dynamics in tropical forests and the SLM parameterized as in this work is that the former assume species-symmetry: the microscopic rates of birth, death, migration fluctuate over time with equal statistical properties across species. The latter does not. Interestingly, in this work, the motivation for refuting species-symmetric models comes mainly from static data, which are typically considered to be well explained by neutral, species-symmetric, models. In fact, most of the analysis and predictions of Neutral Theory focus on SADs. Also in the context of microbial communities, previous works have focused on the shape of the SADs^17,58^. As explained in the introduction, the variation in the SADs come from two fundamentally different sources: the variation of abundance of each species across communities and/or time and the variation of typical abundance across species. By disentangling the regularities in these sources of variation into AFD and MAD, it is possible to show that species-symmetric models cannot explain the large variation in MAD.

Comparing the patterns and the processes between tropical forests and microbial communities is extremely tempting: they are both large, diverse, communities. Whether the regularities of SADs are the byproduct of regularities in the AFDs and MAD also in tropical forests is an open and interesting question. The fact that environmental fluctuations seem to be responsible of the variation in both is also suggestive. When drawing comparison it is important however to confront the issue of scales. More than 3 × 10^13^ bacterial cells live in a single adult human colon^59^, which is about 10 times the number of trees on our whole planet^60^. A year of temporal data is estimated to correspond to about 500 generations for bacteria^44^, which would correspond to 25,000 years of data assuming 50 years generation time for trees^61^. The spatial and temporal scale of observation has in fact fundamental effect on the processes that appear to determine community variation^62^, with demographic stochasticity becoming more important at small spatio-temporal scales and environmental effects more relevant at larger scales.

In microbial communities, the SLM predicts the Gamma AFD and properties of temporal dynamics. Importantly, Taylor’s law and the Lognormal MAD are not predicted by the SLM, but they strongly constrain the parameterization of the SLM. For Taylor’s law, this result parallels the observation that any exponent can be obtained by any family of distribution, provided some mild conditions^63^. As shown in the Methods section in the case of a neutral model with species-dependent migration rate, Taylor’s law constrains parameters variability across species.

The mechanism at the origin of species average abundance and of the robust emergence of the Lognormal MAD remains instead as an open question. The literature on Lognormal SAD is vast^64,65^. It is known to perform well as a statistical model in describing the empirical shape of SAD in tropical forests as an alternative to neutral theory predictions^66^. It also describes reasonably well the empirical shape of SAD in microbial communities^17^. This success in describing the empirical shape of SADs does not parallel with a mechanistic understanding of its emergence in terms of fundamental ecological processes^67^. In this context, our results show that the Lognormality of the SAD in microbial community is only apparent and results as a consequence of the Lognormality of the MAD. This observation has important mechanistic consequences: the origin of Lognormality has to be found in the processes that set species typical abundance and not in the processes determining abundance variability and fluctuations. One interesting direction would be to explore the scaling of average abundances with other physiological parameters (e.g., typical cell size).

The main factor responsible of species abundance fluctuations appears to be environmental stochasticity. It is important to stress that both biotic and abiotic factors contribute to environmental noise. These fluctuations effectively capture multiple biological processes. For instance, the concentration of resources available to a given species constantly fluctuate because the abundance of competitors and cross-feeders fluctuate as well. In large, diverse, communities these fluctuation sum up and result effectively in fast environmental noise. These considerations can be more formally derived in the context of large interacting dynamical systems, where an effective description of single-species dynamics can be obtained^42,68^.

The combination of several complex processes determines the ultimate composition of microbial communities. Their complexity inevitably leads to the emergence of robust and predictive laws. The characterization of such laws, at multiple spatial, temporal, and taxonomic scale, will help in disentangling and quantifying the ecological forces responsible of the stunning (microbial) biodiversity of our planet.

## Methods

### Data

All the data sets analyzed in this work have been previously published and were obtained from EBI Metagenomics^69^. Previous publications (Supplementart Table 1) report the original experiments and corresponding analysis. In order to test the robustness of the macroecological laws and the modeling framework presented in this work, we considered 7 data sets that differ not only for the biome considered, but also for the sequencing techniques and the pipeline used to process the data. Data sets were selected to represent a wide set of biomes. We considered only data sets with at least 50 samples with more than 10^4^ reads. No data set was excluded a-posteriori.

### Sampling and compositional data

In order to study how (relative) abundance varies across communities and species, one needs to remove the effect of sampling noise, as it is not a biologically informative source of variation. By explicitly modeling sampling (Supplementary Note 2), one finds that the probability of observing *n* reads of species *i* in a sample with *N* total number of reads, is given by

6 $${P}_{i}(n| N)=\int_{0}^{1}dx\,{\rho }_{i}(x)\,\left(\begin{array}{*{20}{c}} {n} \\ {N} \end{array}\right){x}^{n}{(1-x)}^{N-n}\,,$$

where *ρ*_*i*_(*x*) is the AFD, i.e., the probability (over communities or times) that the relative abundance of *i* is equal to *x*. Note that this equation does not assume anything about independence across species or communities. It only assumes the sampling process is carried independently across communities.

Since the random variable *x*_*i*_, whose distribution is *ρ*_*i*_(*x*), is a relative abundance, one has that ∑_*i*_*x*_*i*_ = 1 (i.e., the data are compositional^70^). As discussed in Supplementary Note 2, given the range of variation of the empirical relative abundances, one can substitute Eq. (6) with

7 $${P}_{i}(n| N)=\int_{0}^{\infty }dx\,{\rho }_{i}(x)\frac{{(xN)}^{n}}{n!}{e}^{-xN}\,,$$

and the condition $\sum$_*i*_*x*_*i*_ = 1 to ${\sum }_{i}{\bar{x}}_{i}=1$, where ${\bar{x}}_{i}=\mathop{\int}\nolimits_{0}^{\infty }dx\,{\rho }_{i}(x)x$ is the mean value of *x*_*i*_. Under this assumption, one can also take the limits of the integration from 0 to *∞*, instead of considering them from 0 to 1, as the contribution of the integrand from 1 to *∞* is negligible.

Note that, because of sampling, the average of a function *f*(*x*) over the pdf *ρ*(*x*) differs in general from the average of *f*(*n*/*N*) over *P*(*n*∣*N*)

8 $$\int_{0}^{1}dx\,\rho (x)f(x)\, \ne\, \sum _{n = 0}^{N}P(n| N)f\left(\frac{n}{N}\right)=\int_{0}^{1}dx\,\rho (x)\sum _{n = 0}^{N}f\left(\frac{n}{N}\right)\frac{{(xN)}^{n}}{n!}{e}^{-xN}\,,$$

and the inequality becomes equality only if *f*(*x*) is linear. The important difference between the right- and the left-hand side is often neglected in the literature. In fact, the right-hand side is a good approximation of the left-hand size only in the limit *xN* ≫ 1, which is far from being realized in the data for most of the species. Supplementary Note 2 introduces a method to reconstruct the moments of *ρ*(*x*) from the moments of *P*(*n*∣*N*). More generally, I show that it is possible to infer the moment generating function of *ρ*(*x*) from the data, which allow to reconstruct the shape of the empirical *ρ*(*x*).

### Excluding competitive exclusion

A Gamma-distributed AFD implies that all the species present in a community of a biome are present in all the communities from that biome. Therefore, when a species is not observed is because it is undetected due to sampling errors. I test this claim in two different ways. First, it is shown that one can in fact predict the occupancy of a species from its abundance fluctuations. Secondly, I show that a model without true absences is statistically more supported than a model where species are allowed to be absent.

The first way to test this hypothesis is to directly test its immediate prediction: if the absence is a consequence of sampling, one should be able to predict occupancy of a species (the probability that a species is present) simply from its average and variance of abundance (together with the total number of reads of each sample). In particular, assuming a Gamma AFD, the occupancy of species *i* is given by

9 $$\langle {o}_{i}\rangle =1-\frac{1}{T}\sum _{s}P(0| {N}_{s})=1-\frac{1}{T}\sum _{s = 1}^{T}{\left(1+\frac{{\bar{x}}_{i}{N}_{s}}{{\beta }_{i}}\right)}^{-{\beta }_{i}}\,,$$

where *N*_*s*_ is the total number of reads in sample *s*, *T* is the total number of samples, and ${\beta }_{i}={\bar{x}}_{i}^{2}/{\sigma }_{{x}_{i}}^{2}$. As shown in Fig. 2 and in Supplementary Fig. 3, this prediction well reproduces the observed occupancy across species. The prediction of Eq. (12) also matches the occupancy of temporal (longitudinal) data Supplementary Fig. 20.

The second, more rigorous, way to test the hypothesis that (most) species are always present is to use model selection. In this context we want to compare two (or more) models that aim at describing the observed number of reads of each species starting from alternative hypothesis. In particular I compare a purely Gamma AFD with a zero-inflated Gamma, which reads

10 $${\varrho }_{i}(x| {\vartheta }_{i},{\beta }_{i},{\bar{x}}_{i})={\vartheta }_{i}\delta (x)+(1-{\vartheta }_{i})\frac{1}{\Gamma ({\beta }_{i})}{\left(\frac{{\beta }_{i}}{{\bar{x}}_{i}}\right)}^{{\beta }_{i}}{x}^{{\beta }_{i}-1}\exp \left(-{\beta }_{i}\frac{x}{{\bar{x}}_{i}}\right)\,,$$

where *ϑ*_*i*_ is the probability that a species is truly absent in a community and *δ*( ⋅ ) is the Dirac delta distribution. Our goal is to test whether the *ϑ*_*i*_s are significantly different from zero. Since the two models are nested, one can compare the maximum likelihood estimator in the case *ϑ*_*i*_ = 0 with the (maximum) likelihood marginalized over *ϑ* (which has prior *μ*(*ϑ*)). Given the number of reads ${n}_{i}^{s}$ of species *i* in community *s*, with *N*_*s*_ total number of reads, one can compute the ratio (Supplementary Note 4)

11 $${\ell }_{i}=\frac{\mathop{\max }\nolimits_{\bar{x},\beta }{\prod }_{s}\int\ dx{\varrho }_{i}(x| 0,\beta ,\bar{x})\frac{{(x{N}_{s})}^{{n}_{i}^{s}}}{{n}_{i}^{s}!}{e}^{-x{N}_{s}}}{\int\ d\vartheta \,\mu (\vartheta )\left(\mathop{\max }\nolimits_{\bar{x},\beta }{\prod }_{s}\int\ dx{\varrho }_{i}(x| \vartheta ,\beta ,\bar{x})\frac{{(x{N}_{s})}^{{n}_{i}^{s}}}{{n}_{i}^{s}!}{e}^{-x{N}_{s}}\right)}\,,$$

where *μ*(*ϑ*) is a prior over *ϑ*. If *ℓ*_*i*_ > 1, the model with *ϑ*_*i*_ = 0 is more strongly supported than the model with *ϑ* ≠ 0. Under Beta prior with parameters 0.25 and 8, one obtains that *ℓ*_*i*_ > 1 in 98.8% of the cases (averaged across data sets, ranging from 94.4 to 99.7%) and *ℓ*_*i*_ > 100 in 97.5% cases (ranging from 92.8 to 99.2%). See Supplementary Note 4 for a more detailed description of the methodology and Supplementary Fig. 6 for results obtained with other priors.

### Prediction of macroecological patterns

Given laws #1, #2, and #3, the probability to observe *n* reads of a randomly chosen species in a sample with *N* total reads is

12 $$P(n| N)=\int_{-\infty }^{\infty }d\eta \,\frac{\Gamma (\beta +n)}{n!\Gamma (\beta )}{\left(\frac{{e}^{\eta }N}{\beta +{e}^{\eta }N}\right)}^{n}{\left(\frac{\beta }{\beta +{e}^{\eta }N}\right)}^{\beta }\frac{\exp \left(-\frac{{(\eta -\mu )}^{2}}{2{\sigma }^{2}}\right)}{\sqrt{2\pi {\sigma }^{2}}}\,,$$

where $\eta ={\mathrm{log}}\,(\bar{x})$. All the properties of species are fully specified by its mean abundance $\bar{x}={e}^{\eta }$. The probability of observing *n* reads of species with average abundance $\bar{x}$ in a sample with *N* total number of reads is therefore

13 $$P(n| N,\bar{x})=\frac{\Gamma (\beta +n)}{n!\Gamma (\beta )}{\left(\frac{\bar{x}N}{\beta +\bar{x}N}\right)}^{n}{\left(\frac{\beta }{\beta +\bar{x}N}\right)}^{\beta }\,.$$

The predictions for the patterns shown in Fig. 3 are reported here. The full derivation of this and other patterns is presented in Supplementary Note 8.

The total number of observed species in a sample with *N* total number of reads can be easily calculated using Eq. (12). The probability of not observing a species is simply *P*(0∣*N*). The expected number of distinct species 〈*s*(*N*)〉 in a sample with *N* reads is therefore

14 $$\langle s(N)\rangle ={s}_{tot}\left(1-P(0| N)\right)={s}_{tot}\left(1-\int_{-\infty }^{\infty }d\eta \,\frac{\exp \left(-\frac{{(\eta -\mu )}^{2}}{2{\sigma }^{2}}\right)}{\sqrt{2\pi {\sigma }^{2}}}\,{\left(\frac{\beta }{\beta +{e}^{\eta }N}\right)}^{\beta }\right)\,,$$

where *s*_tot_ is the total number of species in the biome (including unobserved ones, see Supplementary Note 7). Note that *s*_tot_ is (substantially) larger than *s*_obs_, the number of different species observed in the union of all the communities, which can instead be written as

15 $$\langle {s}_{obs}\rangle ={s}_{tot}\left(1-\int_{-\infty }^{\infty }d\eta \,\frac{\exp \left(-\frac{{(\eta -\mu )}^{2}}{2{\sigma }^{2}}\right)}{\sqrt{2\pi {\sigma }^{2}}}\,{\left(\prod _{s = 1}^{T}\frac{\beta }{\beta +{e}^{\eta }{N}_{s}}\right)}^{\beta }\right)\,.$$

Figure 3a shows that the prediction of Eq. (14) correctly matches the data (Supplementary Fig. 13).

The SAD, one of the most studied patterns in ecology and directly related to the Relative Species Abundance^35^, is defined as the fraction of species with a given abundance. According to our model, the expected SAD is given by

16 $$\langle {\Phi }_{n}(N)\rangle := \frac{\langle {s}_{n}(N)\rangle }{\langle s(N)\rangle }=\frac{P(n| N)}{1-P(0,N)}=\frac{\int_{-\infty }^{\infty }d\eta \,\frac{\Gamma (\beta +n)}{n!\Gamma (\beta )}{\left(\frac{{e}^{\eta }N}{\beta +{e}^{\eta }N}\right)}^{n}{\left(\frac{\beta }{\beta +{e}^{\eta }N}\right)}^{\beta }\frac{\exp \left(-\frac{{(\eta -\mu )}^{2}}{2{\sigma }^{2}}\right)}{\sqrt{2\pi {\sigma }^{2}}}}{1-\int_{-\infty }^{\infty }d\eta \,{\left(\frac{\beta }{\beta +{e}^{\eta }N}\right)}^{\beta }\,\frac{\exp \left(-\frac{{(\eta -\mu )}^{2}}{2{\sigma }^{2}}\right)}{\sqrt{2\pi {\sigma }^{2}}}}\,,$$

where 〈*s*_*n*_(*N*)〉 is the number of species with *n* reads in a sample with *N* total number of reads. The cumulative SAD is defined as

17 $$\langle {\Phi }_{n}^{ \,{> }\,}(N)\rangle := \sum _{m = n}^{\infty }\langle {\Phi }_{m}(N)\rangle =\frac{\int\ \mathop{\int}\nolimits_{-\infty }^{\infty }\,{I}_{\frac{{e}^{\eta }N}{\beta +{e}^{\eta }N}}(n,\beta )\frac{\exp \left(-\frac{{(\eta -\mu )}^{2}}{2{\sigma }^{2}}\right)}{\sqrt{2\pi {\sigma }^{2}}}}{1-\mathop{\int}\nolimits_{-\infty }^{\infty }\eta \,{\left(\frac{\beta }{\beta +{e}^{\eta }N}\right)}^{\beta }\,\frac{\exp \left(-\frac{{(\eta -\mu )}^{2}}{2{\sigma }^{2}}\right)}{\sqrt{2\pi {\sigma }^{2}}}}\,,$$

where *I*_*p*_(*n*, *β*) is the regularized incomplete Beta function. Figure 3b shows that the Eq. (17) captures the empirical cumulative SAD (Supplementary Fig. 17).

The occupancy probability is defined as the probability that a species is present in a given fraction of communities. This quantity has been extensively studied in a variety of contexts (from genomics^71^ to Lego sets and texts^72^) and has been more recently considered in microbial ecology^37^. The three macroecological laws predict (see derivation in Supplementary Note 8)

18 $${p}_{obs}(o)=\frac{\mathop{\int}\nolimits_{-\infty }^{\infty }d\eta \,\sum_{t = 1}^{T}\delta \left(o-1+\frac{1}{T}\mathop{\sum }\nolimits_{s = 1}^{T}{\left(\frac{\beta }{\beta +{e}^{\eta }{N}_{s}}\right)}^{\beta }\right)\frac{\exp \left(-\frac{{(\eta -\mu )}^{2}}{2{\sigma }^{2}}\right)}{\sqrt{2\pi {\sigma }^{2}}}\mathop{\prod }\nolimits_{s = 1}^{T}\left(1-{\left(\frac{\beta }{\beta +{e}^{\eta }{N}_{s}}\right)}^{\beta }\right)}{\mathop{\int}\nolimits_{-\infty }^{\infty }d\eta \,\frac{\exp \left(-\frac{{(\eta -\mu )}^{2}}{2{\sigma }^{2}}\right)}{\sqrt{2\pi {\sigma }^{2}}}\,\mathop{\prod }\nolimits_{s = 1}^{T}\left(1-{\left(\frac{\beta }{\beta +{e}^{\eta }{N}_{s}}\right)}^{\beta }\right)}\,,$$

where *δ*( ⋅ ) is a Dirac delta function. Figure 3c compares the prediction of Eq. (18) with the data (Supplementary Fig. 15).

Occupancy (the fraction of communities where a species is found) and abundance are not independent properties, and their relative dependence is often referred to as occupancy-abundance relationship^21^ Given an average (relative) abundance $\bar{x}=\exp (\eta )$, the expected occurrence is

19 $${\langle o\rangle }_{\eta }=1-\frac{1}{T}\sum _{s = 1}^{T}P(0| {N}_{s},\bar{x})=1-\frac{1}{T}\sum _{s = 1}^{T}{\left(\frac{\beta }{\beta +\bar{x}{N}_{s}}\right)}^{\beta }\,,$$

Figure 3d shows the comparison between data and predictions (Supplementary Fig. 16). These predictions are also tested for temporal (longitudinal) data in Supplementary Figs. 22–24.

### Transition probabilities in longitudinal data

For longitudinal data, in addition to the stationary AFD, one can study the probability ${\rho }_{i}(x^{\prime} ,t+\Delta t| x,t)$ that a species *i* has abundance $x^{\prime}$ at time *t* + Δ*t* conditioned on having abundance *x* at time *t*. Instead of focusing on the full distribution, we study its first two (conditional) central moments, i.e. the average and variance of the abundance at *t* + Δ*t* conditioned to abundance *x* at time *t*. In the analysis of the data stationarity is assumed (the distribution ${\rho }_{i}(x^{\prime} ,t+\Delta t| x,t)$ depends on Δ*t* but not on *t*). I also assume that the dynamics of different species are governed by similar equations that only differ in their parameters. One would like therefore to average over species, by properly rescaling their abundances. The average over species is potentially problematic, as it could add a spurious effect to the conditional averages. For instance, only species with larger fluctuations would appear for extreme values of the initial abundance. In order to avoid these problems, instead of consider the actual abundance, its cumulative probability distribution value (calculated using the empirical AFD of each species) was used, that is referred as “quantile abundance”. This is equivalent to rank the abundances of each species over communities and use the (relative) ranking of each community instead of the abundance. A value equal to 0 corresponds to the lowest observed abundance, and a value equal to 1 to the highest. By definition, the quantile abundance is always uniformly distributed.

### Ruling out demographic stochasticity

Demographic stochasticity can reproduce a Gamma AFD. A birth, death, and immigration process has a Gamma as stationary distribution^35^. In the limit of large populations sizes, it corresponds to the following equation^35^

20 $$\frac{dx}{dt}=m-(d-b)x+\sqrt{(b+d)x}\xi (t)\,,$$

where *m* is the migration rate, while *b* and *d* are the per-capita birth and death rate. The Gaussian white noise term *ξ*(*t*) has mean zero and time-correlation $\langle \xi (t)\xi (t^{\prime} )\rangle =\delta (t-t^{\prime} )$. The stationary distribution of this process turns out to be

21 $$\rho (x)=\frac{1}{\Gamma \left(2\frac{m}{b+d}\right)}{\left(\frac{b+d}{2(d-b)}\right)}^{-2\frac{m}{b+d}}{x}^{2\frac{m}{b+d}-1}\exp \left(-2\frac{d-b}{b+d}x\right)\,.$$

The average abundance is equal to $\bar{x}=m/(d-b)$, while the variance turns out to be ${\sigma }_{x}^{2}=(m/2)(b+d)/{(b-d)}^{2}$. The square of the coefficient of variation would therefore be equal to (*b* + *d*)/(2*m*).

More generally, one can assume that all the parameters are species dependent, and the population of species *i* is described by

22 $$\frac{d{x}_{i}}{dt}={m}_{i}-({d}_{i}-{b}_{i}){x}_{i}+\sqrt{({b}_{i}+{d}_{i}){x}_{i}}{\xi }_{i}(t)\,,$$

where $\langle {\xi }_{i}(t){\xi }_{j}(t^{\prime} )\rangle ={\delta }_{ij}\delta (t-t^{\prime} )$ was assumed.

Taylor’s Law and the wide variation of average abundance together imply that *m*_*i*_/(*b*_*i*_ + *d*_*i*_) is constant while *m*_*i*_/(*d*_*i*_ − *b*_*i*_) varies across species on several orders of magnitudes. This imposes a constraint on the variation of parameter values across species.

For instance, one can consider the scenario where species migrate to local communities from a common species pool (metacommunity). As abundance in the metacommunity varies across species the migration rate is a species-dependent quantity. Under neutrality, the per-capita birth and death rates in the local communities are constant and independent of the identity of the species. In this case *m*_*i*_ depends on the species, while *b* and *d* do not. One could recover the Lognormal MAD by imposing that *m*_*i*_ is Lognormally distributed. On the other hand, this model would fail in reproducing Taylor’s law with exponent 2, as it would predict and exponent 1.

More in general, the condition imposed on the parameters corresponds to an unnatural fine-tuned relationship between migration, birth, and death rates. Variation of the average abundance is observed across, at least, 7 orders of magnitudes. In order to reproduce this variation across species and Taylor’s law with exponent 2, the range of variability of (*b*_*i*_ − *d*_*i*_)/(*b*_*i*_ +  *d*_*i*_) should be of the same order. It is unrealistic that the relative difference between birth and death rates, which have strong and direct connection to fundamental biological processes, vary so much across bacterial species. It is important to underline however, that the model of Eq. (22) can, in fact, for a proper parameterization, explain the observed variation of the data. But the choice of parameters explaining the empirical variation require for achieving this goal requires careful and unrealistic fine-tuning of the microscopic parameters.

### Stochastic logistic model

The SLM is defined as

23 $$\frac{d{x}_{i}}{dt}=\frac{{x}_{i}}{{\tau }_{i}}\left(1-\frac{{x}_{i}}{{K}_{i}}\right)+\sqrt{\frac{{\sigma }_{i}}{{\tau }_{i}}}{x}_{i}{\xi }_{i}(t)\,,$$

where *ξ*(*t*) is a Gaussian white noise term with mean zero and correlation $\langle {\xi }_{i}(t){\xi }_{j}(t^{\prime} )\rangle ={\delta }_{ij}\delta (t-t^{\prime} )$. Taylor’s Law and the observed Lognormal MAD constraints the parameter value. The parameters 1/*τ*_*i*_, *K*_*i*_ and *σ*_*i*_ are the intrinsic growth rate, the carrying capacity and the coefficient of variation of the growth-rate fluctuations. Taylor’s Law requires *σ*_*i*_ = *σ* (independently of *i*). Since the average abundance of the SLM is ${\bar{x}}_{i}={K}_{i}(1-{\sigma }_{i}/2)$, if *σ*_*i*_ = *σ*, the average abundance and the carrying capacity turn out to be proportional to each other. The lognormal MAD implies therefore that the *K*_*i*_s are lognormally distributed. The stationary distribution corresponding to Eq. (23) reads

24 $${\rho }_{i}(x)=\frac{1}{\Gamma (2{\sigma }_{i}^{-1}-1)}{\left(\frac{2}{{K}_{i}{\sigma }_{i}}\right)}^{2{\sigma }_{i}^{-1}-1}\exp \left(-\frac{2}{{K}_{i}{\sigma }_{i}}x\right){x}^{2{\sigma }_{i}^{-1}-2}\,.$$

The parameter *τ*_*i*_ does not affect stationary properties, but determines the timescale of relaxation to the stationary distribution. For small deviation of abundance from the average and for large times, the conditional expected abundance behaves as

25 $${\langle {x}_{i}(t+\Delta t)\rangle }_{{x}_{i}(t)}={\bar{x}}_{i}+\left({x}_{i}(t)-{\bar{x}}_{i}\right){e}^{-\frac{\Delta t}{{\tau }_{i}}}\,.$$

From the slopes of Fig. 4g one can then determine the timescales *τ*_*i*_, which turn out to be approximately equal to 19 h. In Fig. 4 it was assumed *τ*_*i*_ = 19 h for all species.

Equation (23) can emerge as effective description of more complicated coupled equations. For instance, it is possible to show that a Lotka-Volterra system of equation with random interactions reduces to Eq. (23) (with colored noise to be self-consistently determined)^42^. If the coefficient of variation of the interaction coefficient does not increase with the number of species (e.g., if it is constant) then the Lotka-Volterra equations can be effectively approximated with Eq. (23).

The noise term in Eq. (23) can be interpreted as corresponding to environmental fluctuations. These fluctuations are typically known to have a characteristic timescale and are not white^40,41^. Supplementary Note 13 and Supplementary Fig. 25 show that colored noise in Eq. (23) does not affect significantly the predictions obtained with the SLM with white noise.

### Reporting summary

Further information on research design is available in the Nature Research Reporting Summary linked to this article.

## Supplementary information

Supplementary Information

Reporting Summary

## Data Availability

All the data used in this work were previously published and publicly available (Supplementary Table 1).

## Code availability

Code to carry out analyses is publicly available on Zenodo doi:10.5281/zenodo.3992642 and of Github https://github.com/jacopogrilli/lawsdiv.
