# Supplementary material for: Macroecological laws describe variation and diversity in microbial communities
Source: Nat Commun. 2020 Sep 21;11:4743. doi: 10.1038/s41467-020-18529-y (PMC7506021; doi:10.1038/s41467-020-18529-y)
Supplement: Supplementary file 1 — Supplementary Information [file 41467_2020_18529_MOESM1_ESM.pdf]

Macroecological laws describe variation and diversity in microbial communities

— Supplementary Information —

Grilli

Supplementary Table 1: Description and references for the datasets used in this work. In column 'Type', c refers to cross-sectional (across communities) and l to longitudinal (across time).

Biome ID	Type	EBI ID	Magnify ID	Pipeline Version	NCBI ID	Reference	# Samples T	Range Tot # Reads N_s
glacier	c	ERP017997	MGYS00001292	3.0	PRJEB16145	[1]	30	$[79765, 1104214]$
gut1	c	SRP056641	MGYS00001056	2.0	PRJNA275349	[1]	66	$[13842, 102971]$
gut2	c	ERP015450	MGYS00001556	3.0	PRJEB13870	[2]	195	$[24717, 614229]$
lake	c	ERP012927	MGYS00001669	3.0	PRJEB11530	[3]	198	$[57408, 350877]$
oral1	c	SRP056641	MGYS00001056	2.0	PRJNA275349	[1]	62	$[10006, 138172]$
river	c	ERP012927	MGYS00001669	3.0	PRJEB11530	[3]	188	$[76042, 352675]$
seawater	c	SRP128662	MGYS00002437	4.1	PRJNA429259	[4]	474	$[11260, 492477]$
sludge	c	ERP009143	MGYS00001064	2.0	PRJEB8105	[3]	575	$[22255, 912713]$
soil	c	SRP052295	MGYS00000905	2.0	PRJNA272333	-	112	$[11352, 58219]$
feces F4	l	ERP021896	MGYS00002184	4.1	PRJEB19825	[5]	131	$[21008, 51986]$
feces M3	l	ERP021896	MGYS00002184	4.1	PRJEB19825	[5]	334	$[15047, 58463]$
L_palm F4	l	ERP021896	MGYS00002184	4.1	PRJEB19825	[5]	134	$[12298, 34607]$
L_palm M3	l	ERP021896	MGYS00002184	4.1	PRJEB19825	[5]	365	$[144, 48475]$
R_palm F4	l	ERP021896	MGYS00002184	4.1	PRJEB19825	[5]	134	$[3214, 9052]$
R_palm M3	l	ERP021896	MGYS00002184	4.1	PRJEB19825	[5]	358	$[135, 91953]$
Tongue F4	l	ERP021896	MGYS00002184	4.1	PRJEB19825	[5]	135	$[5683, 12651]$

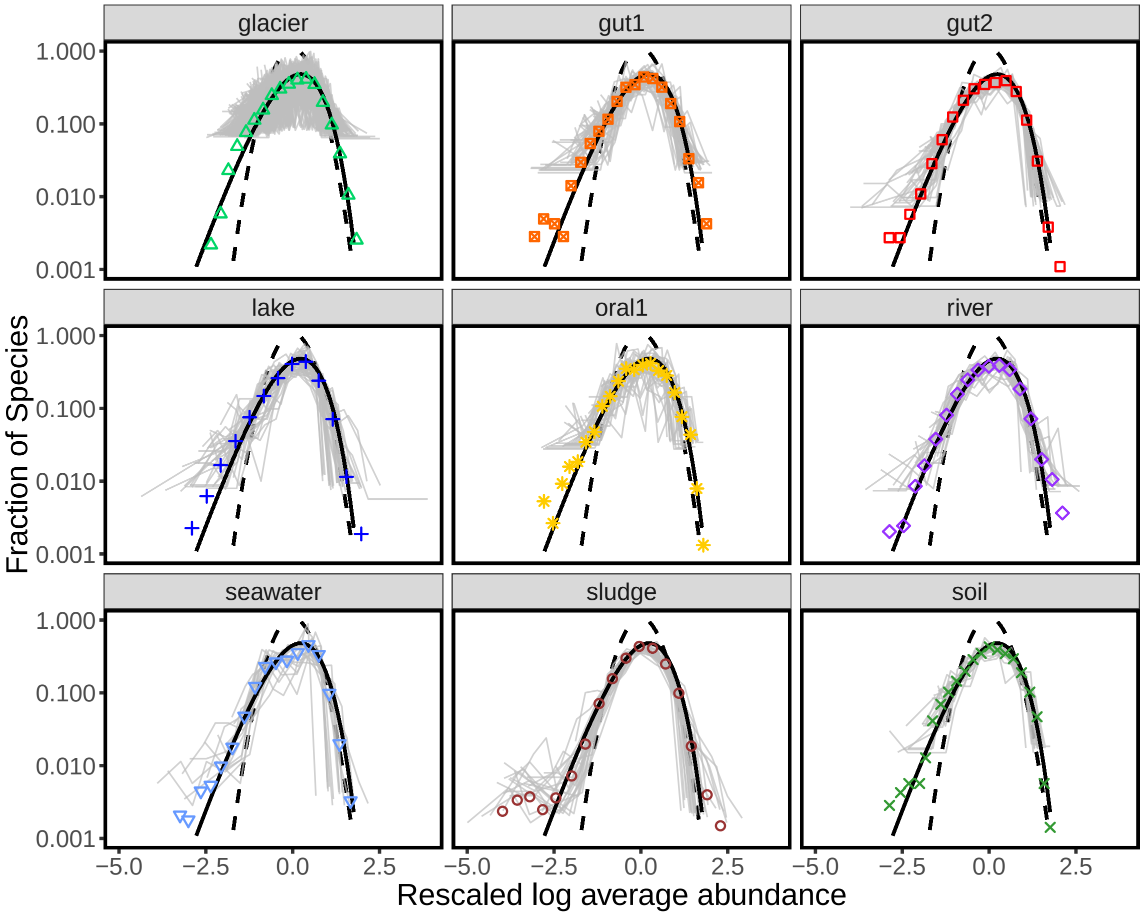

Supplementary Figure 1: **Fluctuations of species abundance.** These panels report exactly the same data shown in 1. For each biome, they were considered only the species present in all the communities. The logarithm of their relative abundances were rescaled (so to have mean zero and unitary variance). The panels report the distribution of these rescaled fluctuations for each biome. Colored points are distributions calculated over both communities and species (same as shown is figure 1). Gray lines are the distribution for individual species over communities. The black continuous line is a Gamma distribution and the black dashed line a Lognormal distribution.

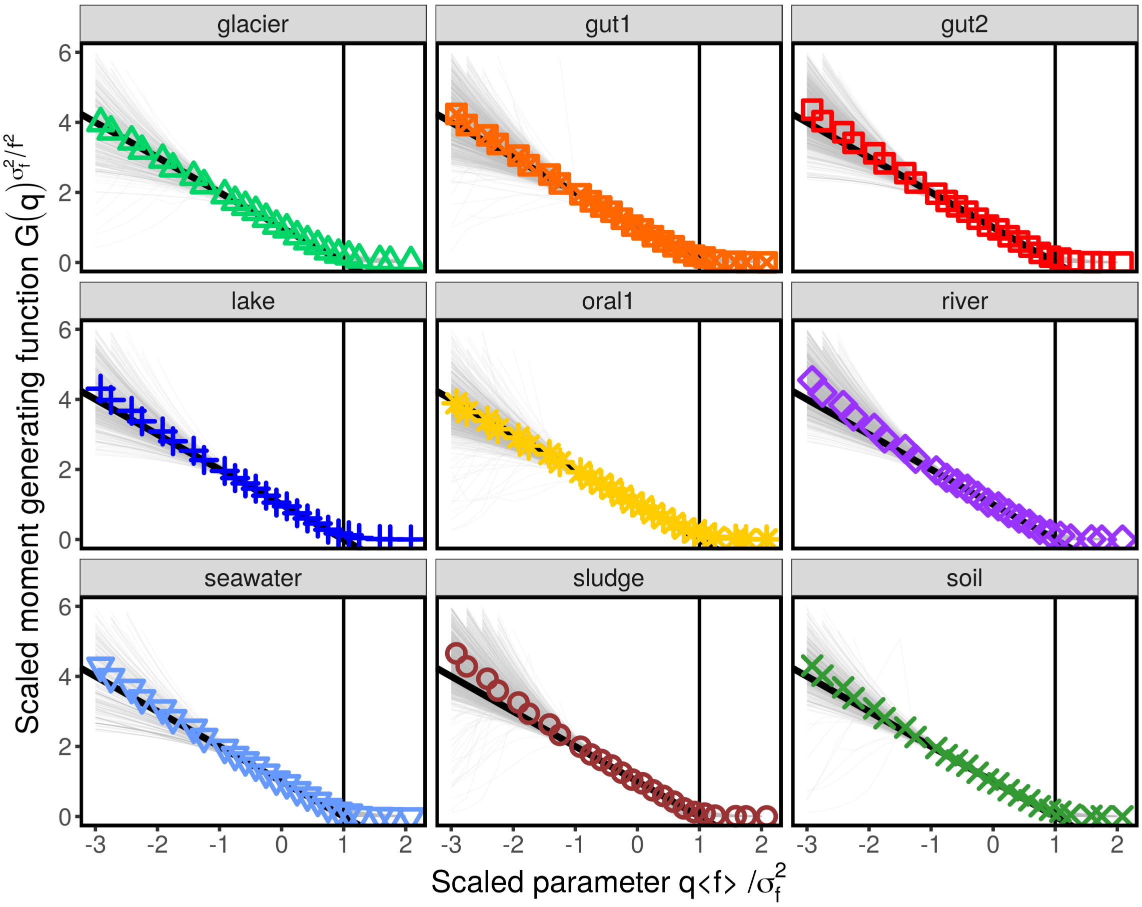

Supplementary Figure 2: **Moment generating function estimated from data.** The panels show the moment generating function estimated from the data using equation 33. The gray lines were obtained for individual species (with average abundance $\bar{x}_i > 5 \cdot 10^{-5}$), while colored points are averages over species. The black solid line is the prediction for the Gamma distribution.

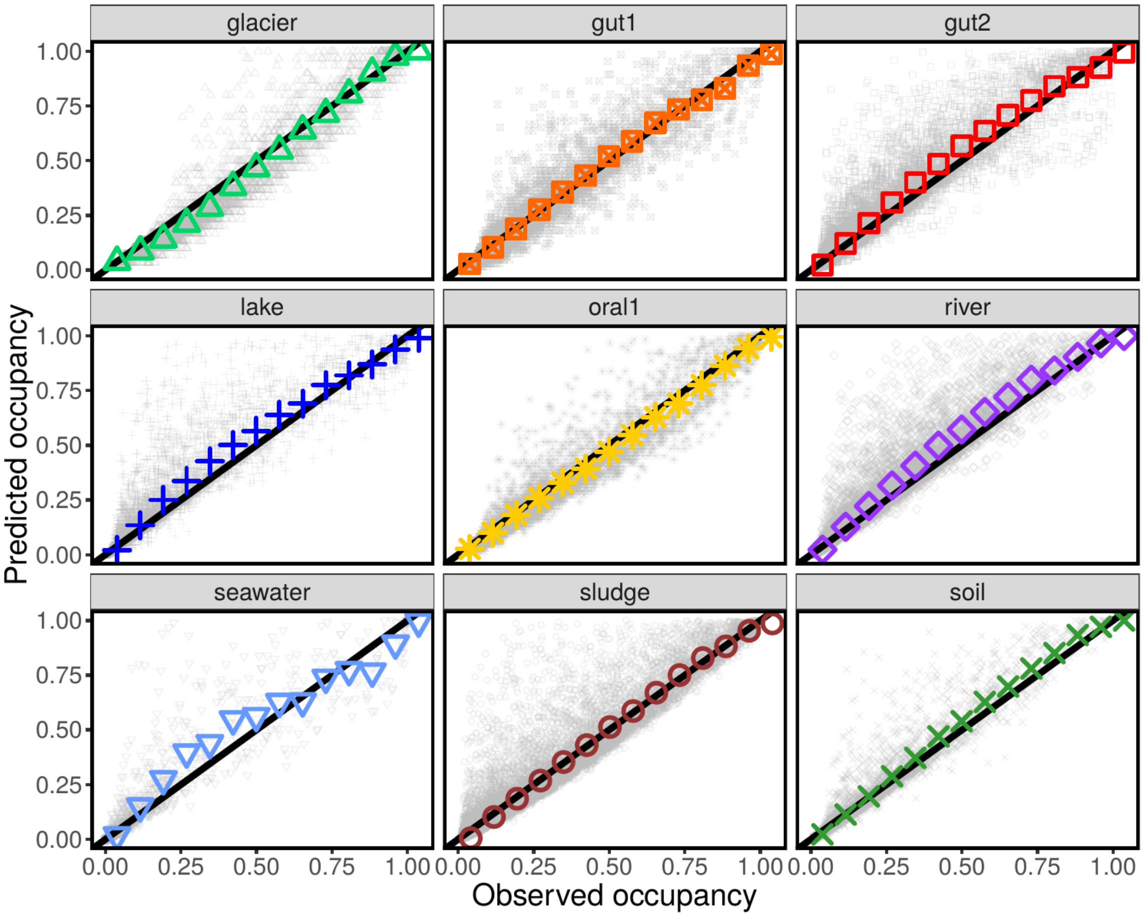

Supplementary Figure 3: **A Gamma AFD correctly predicts species' occupancies.** The occupancy is defined as the fraction of samples/communities where a given species is found to be present. The predicted occupancy was obtained using equation 38, which assumes a Gamma AFD. Using the average and the variance of species' relative abundances, one can in fact estimate the parameters of the AFD and the probability that a species is not found in a sample/community, given the level of sampling. The black line is the 1 : 1 line, indicating a correct prediction. The gray points are individual species (no filter on average abundance was applied), while the colored points are averages over species.

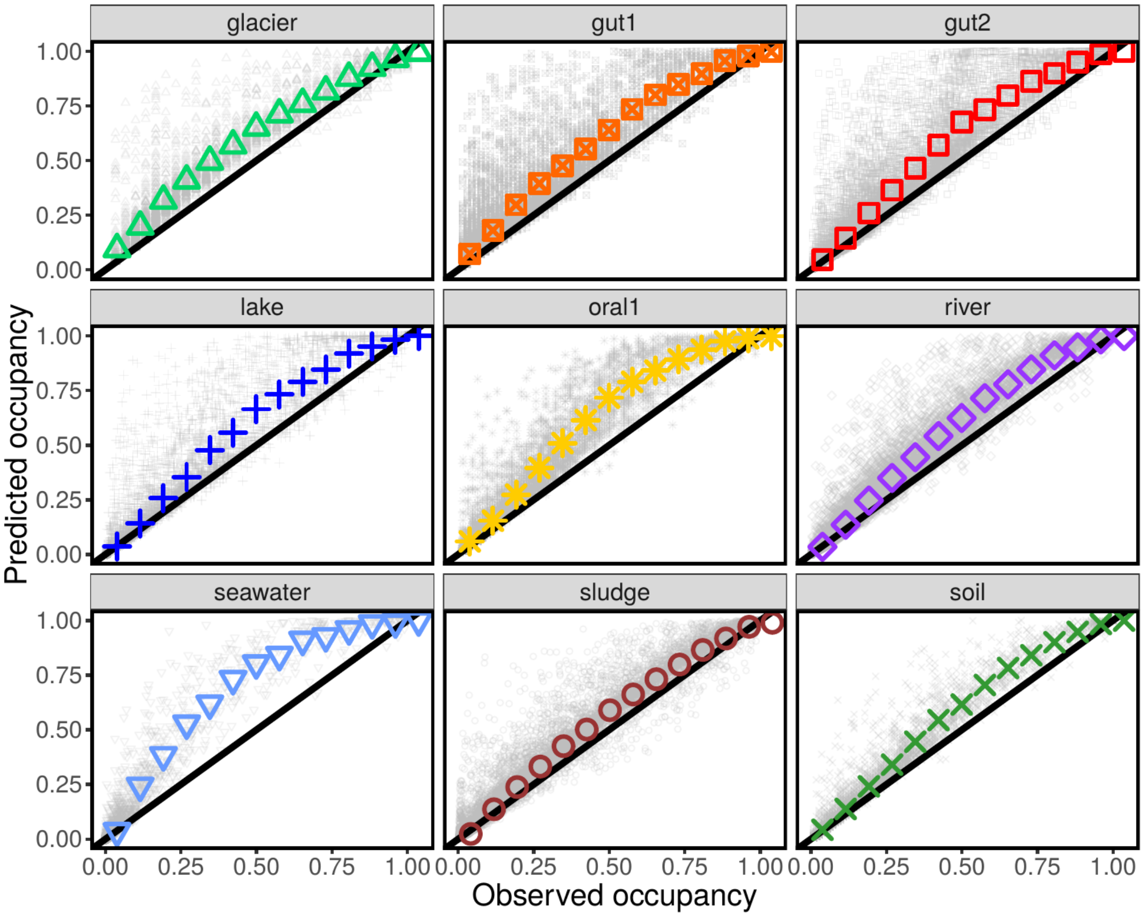

Supplementary Figure 4: **A Lognormal AFD fails in predicting species' occupancies.** The occupancy is defined as the fraction of samples/communities where a given species is found to be present. The predicted occupancy was obtained using equation 39, which assumes a Lognormal AFD. Using the average and the variance of species' relative abundances, one can in fact estimate the parameters of the AFD and the probability that a species is not found in a sample/community, given the level of sampling. The black line is the 1 : 1 line, indicating a correct prediction. The gray points are individual species (no filter on average abundance was applied), while the colored points are averages over species.

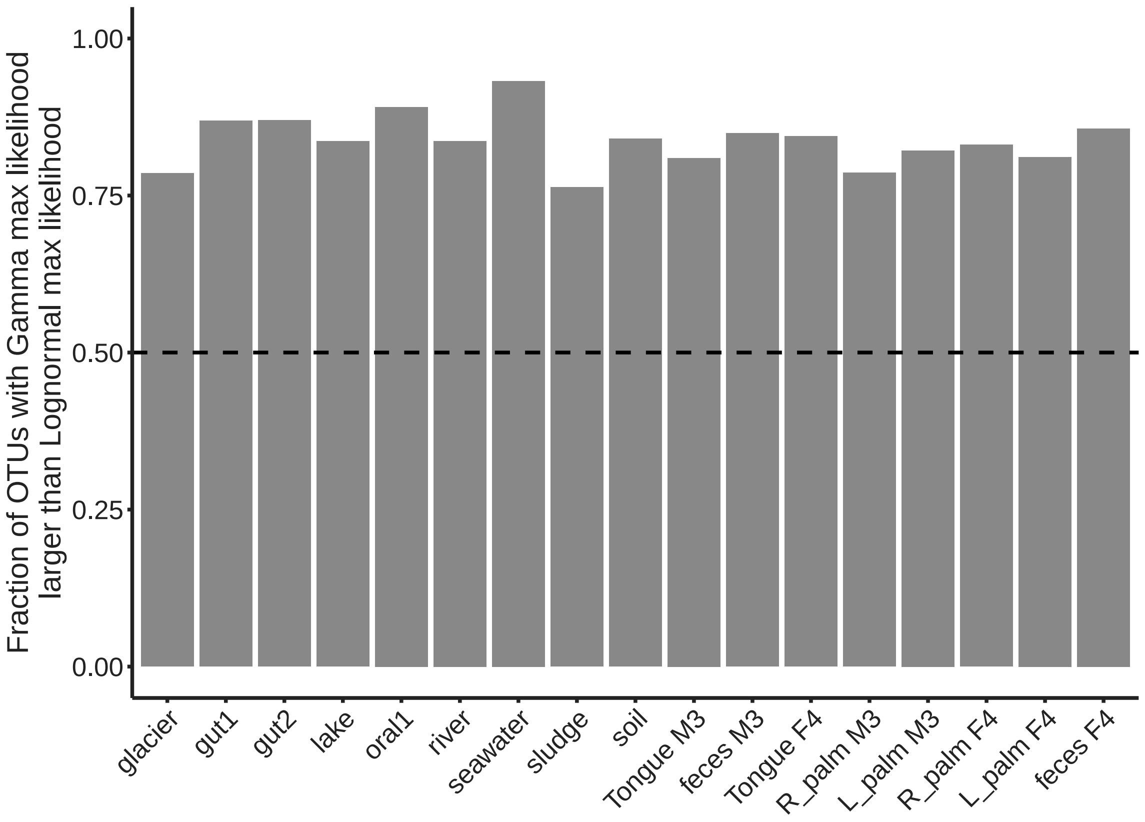

Supplementary Figure 5: **Model selection for AFD.** The bars represent the fraction of OTUs for which a Gamma distribution is statistically preferred (has a larger maximum likelihood) than a Lognormal distribution in describing the AFD.

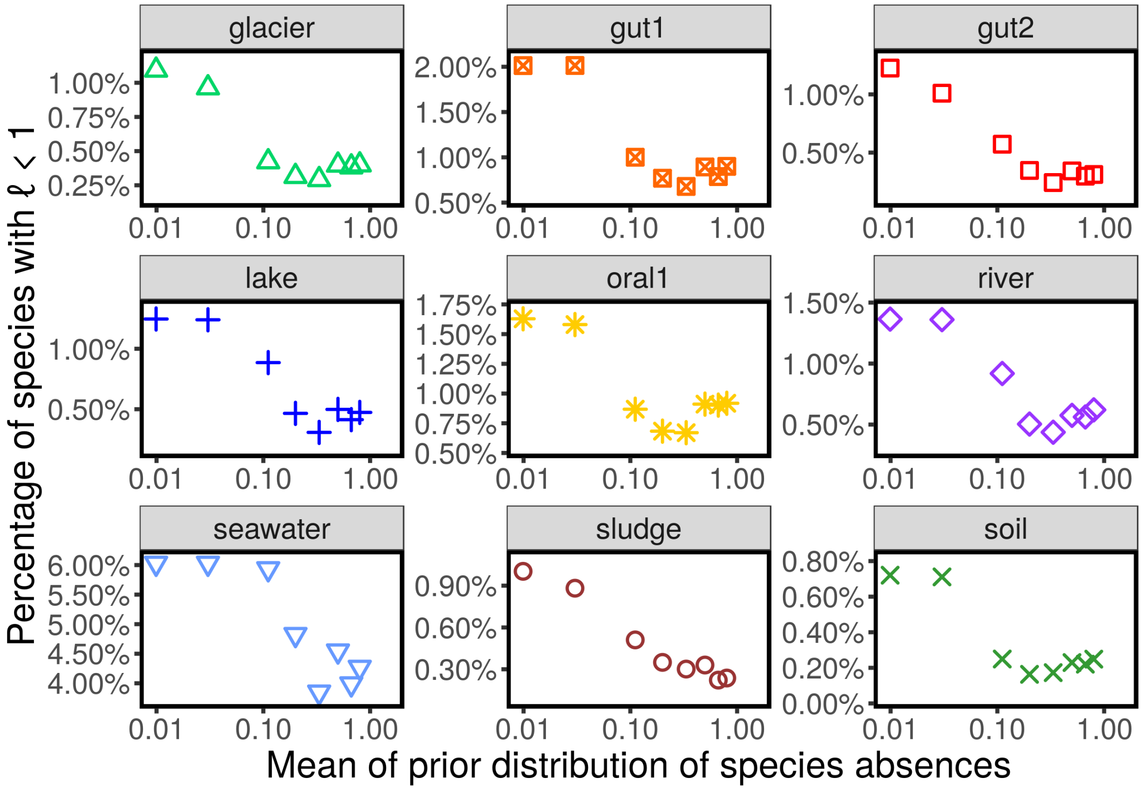

Supplementary Figure 6: **Fraction of species for which exclusion is significant.** The plots show the fraction of species for which the inflated Gamma model (which allows true zeros in the abundance distribution) is more supported by the data then the standard Gamma model (which predicts that all the instances when a species is absent are due to sampling errors), measured as the fraction of species for which $\ell_i < 1$ (see definition in eq. 42). These values are plotted for different choices of the hyper-parameters a and b, showing that the fraction of species with $\ell_i < 1$ decreases with increasing the average probability of a true absence ($a/(a+b)$), on the x-axes).

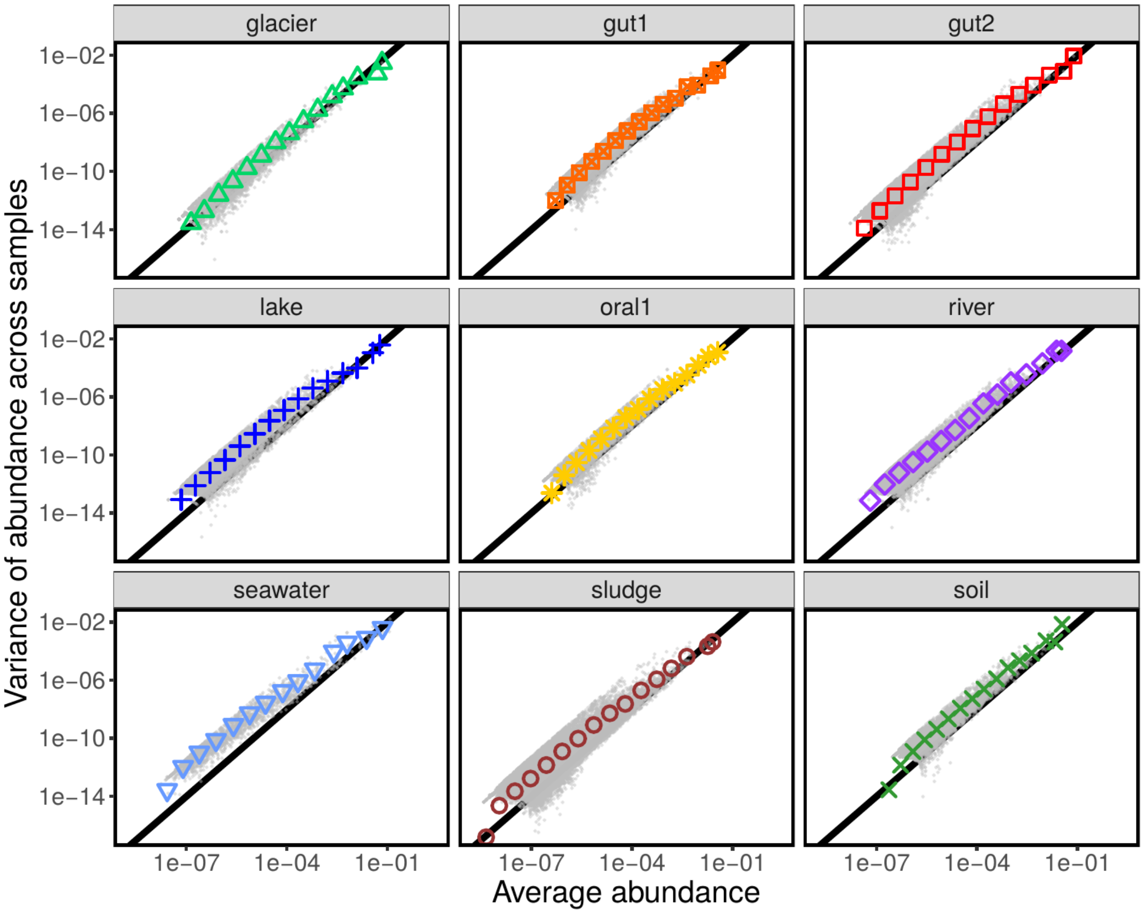

Supplementary Figure 7: **Taylor's law.** The panels show the relationship between mean and variance of abundance. The black line has slope 2, representing the quadratic relationship between variance and mean abundance.

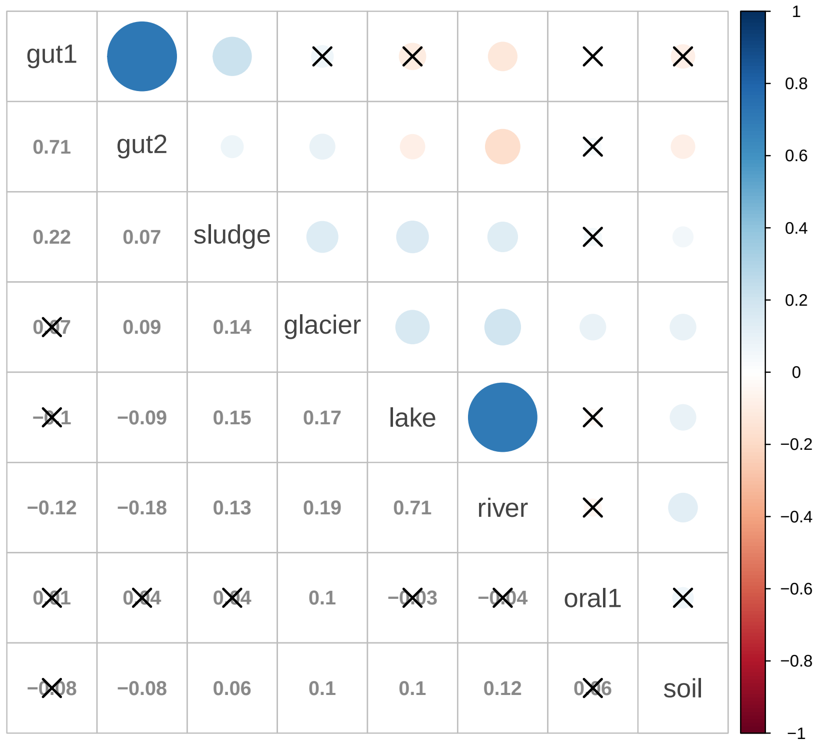

Supplementary Figure 8: **Correlation between average abundances across biomes.** The correlation plot shows the correlation between species average abundances across biomes. The colored circles represent the correlation value (also reported in the lower diagonal part). Crossed circles/values are non-significant correlations (the ones with p-value larger than 0.001). Note that the sample size varies across pairs of biomes as one can consider only species which appeared in both biomes. Seawater was excluded from this plot as the OTU picking method does not match with the ones of other biomes.

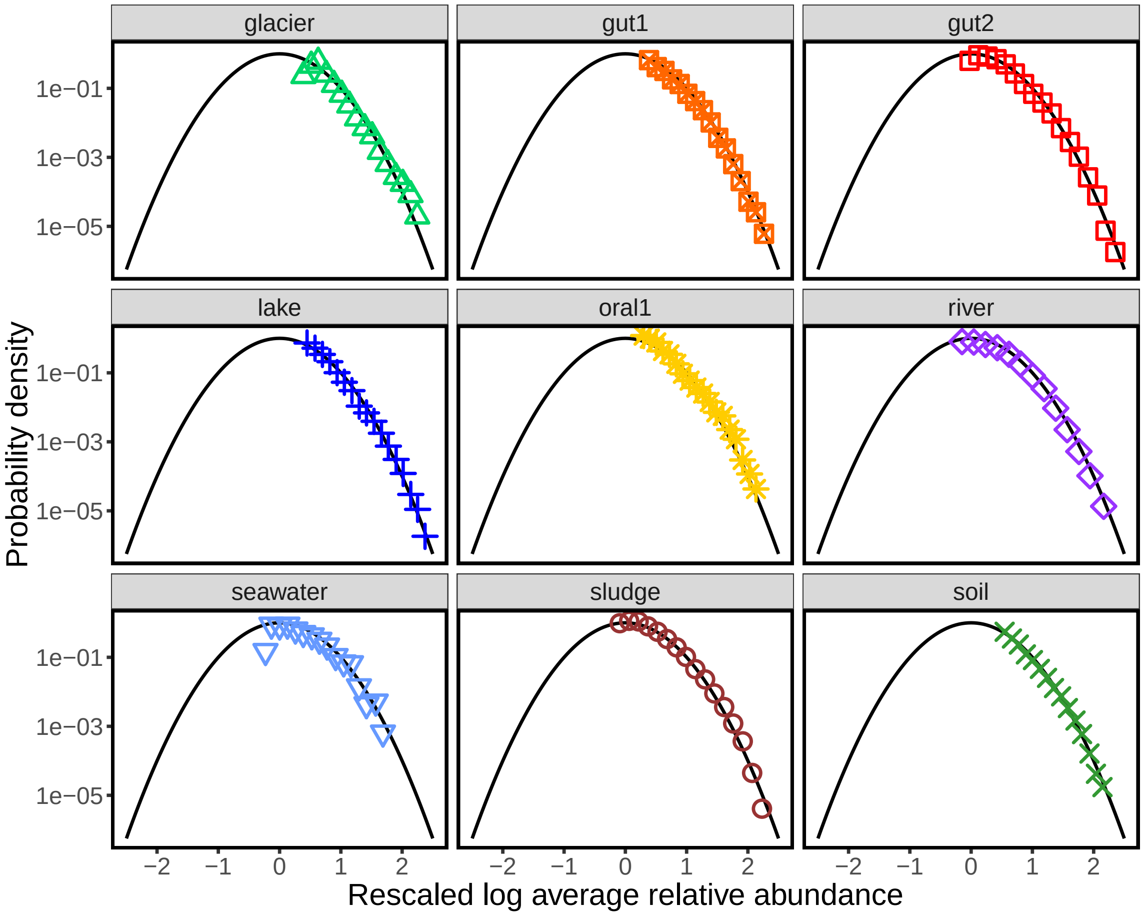

Supplementary Figure 9: **The Mean Abundance Distribution (MAD) is Lognormal.** The panels show the distribution of the logarithm of the average abundance $\log \bar{x}_i$. A Lognormal MAD corresponds to normally distributed $\log \bar{x}_i$. The black line is standardized gaussian distribution (mean zero and variance one). The log average abundances were rescaled as $z_i = (\log \bar{x}_i - \mu)/\sigma$, where μ and σ were obtained for each biome from equation 49. The panels show that the rescaled log average abundances z_i are distributed according to a standard normal, implying that the average abundances $\bar{x}_i$ are lognormally distributed.

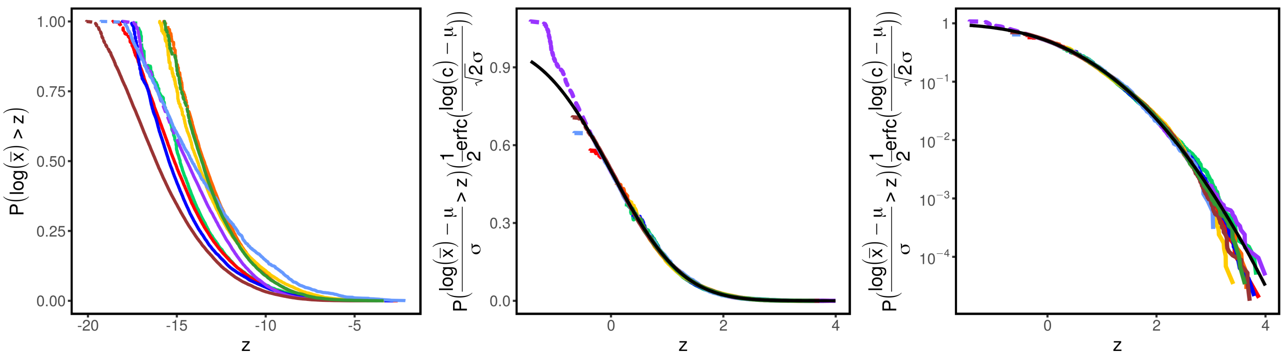

Supplementary Figure 10: **The Mean Abundance Distribution (MAD) is Lognormal.** The left panel shows the cumulative distribution of the average abundance across species for different datasets (colored lines). The center and right panels show that the cumulative distribution collapse if rescaled accordingly to a lognormal distribution with lower cutoff, matching the cumulative distribution of standardized lognormal (black line). The dashed portion of the curves represent the data below the cutoff c.

Supplementary Table 2: Estimate of parameters across biomes and datasets. s_{tot} is the inferred total number of species, μ and σ are the two parameters characterizing the Lognormal MAD (mean and standard deviation of $\log \bar{x}_i$), while β is the shape parameter of the Gamma AFD (which is equal to the inverse coefficient of variation squared of abundance fluctuations).

Biome ID	s_{tot}	μ	σ	β
glacier	20221	-18.5	4.1	1.3
gut1	35469	-16.1	3.5	0.4
gut2	50186	-17.2	3.8	0.3
lake	46912	-19.8	4.4	0.4
oral1	29149	-17.3	4.1	0.4
river	20833	-14.2	2.7	0.3
seawater	3336	-16.2	4.6	0.2
sludge	56671	-17.5	3.7	0.6
soil	29387	-17.1	3.8	0.38
feces F4	1742	-22.1	7.0	3.2
feces M3	5575	-28.0	8.5	1.2
L_palm F4	3429	-14.7	4.1	0.7
L_palm M3	3179	-14.8	4.0	0.3
R_palm F4	2743	-13.7	3.8	0.9
R_palm M3	6547	-18.4	5.2	0.3
Tongue F4	1708	-22.6	7.3	1.5
Tongue M3	2368	-23.9	7.4	1.4

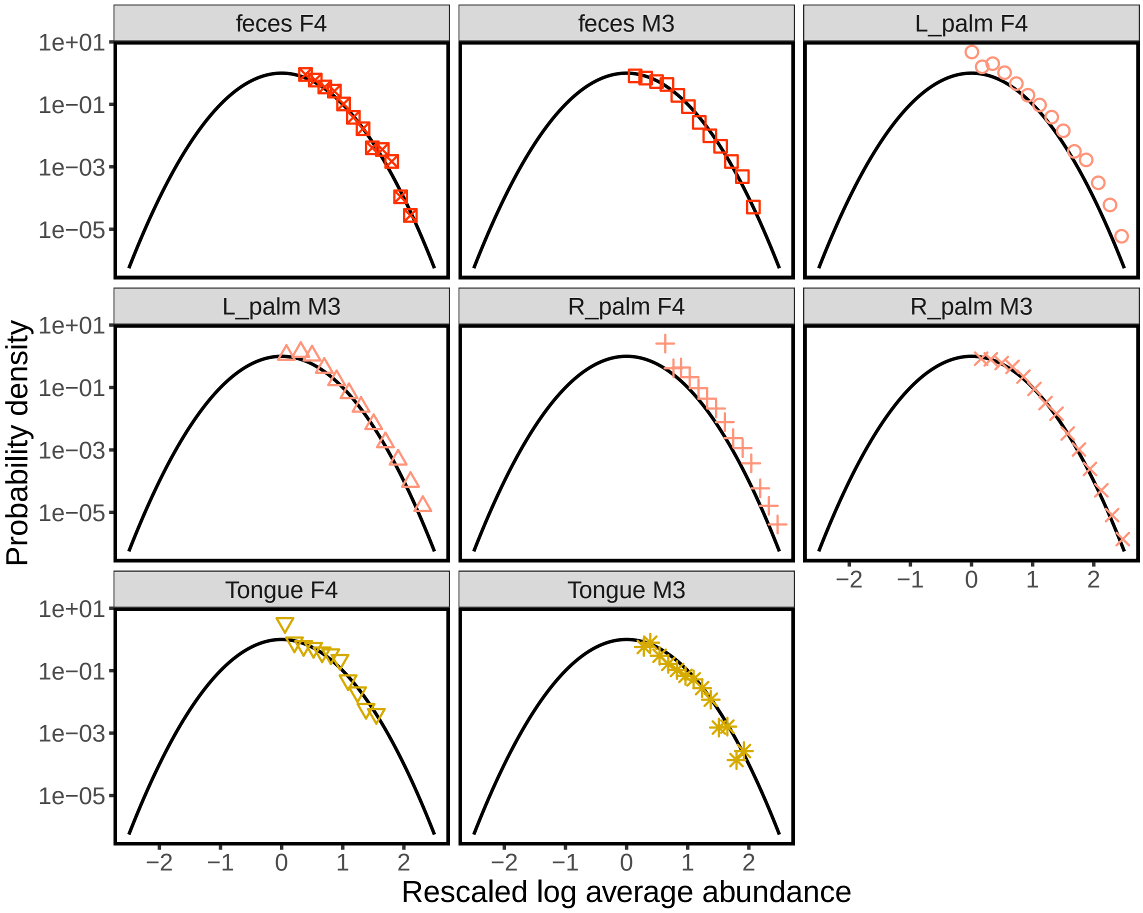

Supplementary Figure 11: **Mean Abundance Distribution (MAD) for time data.** The panels show the distribution of the logarithm of the average abundance $\log \bar{x}_i$. A Lognormal MAD corresponds to normally distributed $\log \bar{x}_i$. The black line is standardized gaussian distribution (mean zero and variance one). The log average abundances were rescaled as $z_i = (\log \bar{x}_i - \mu)/\sigma$, where μ and σ were obtained for each biome from equation 49. The panels show that the rescaled log average abundances z_i are distributed according to a standard normal, implying that the average abundances $\bar{x}_i$ are lognormally distributed.

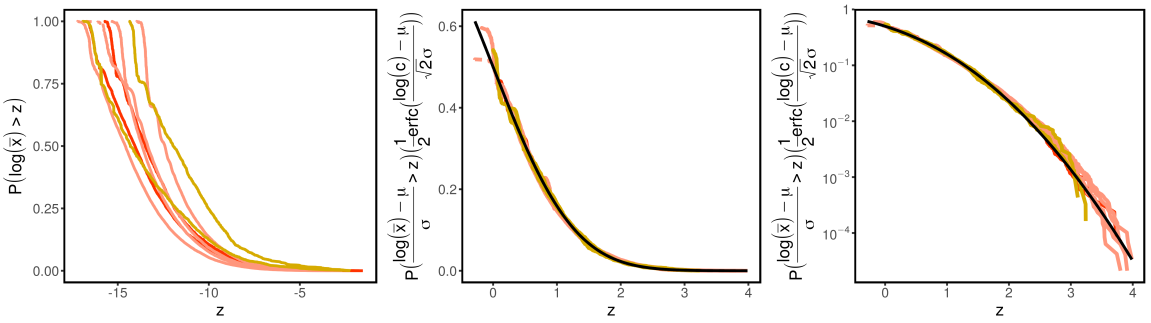

Supplementary Figure 12: **The Mean Abundance Distribution (MAD) is Lognormal for time data.** The left panel shows the cumulative distribution of the average abundance across species for different datasets (colored lines). The center and right panels show that the cumulative distribution collapse if rescaled accordingly to a Lognormal distribution with lower cutoff, matching the cumulative distribution of standardized Lognormal (black line). The dashed portion of the curves represent the data below the cutoff c.

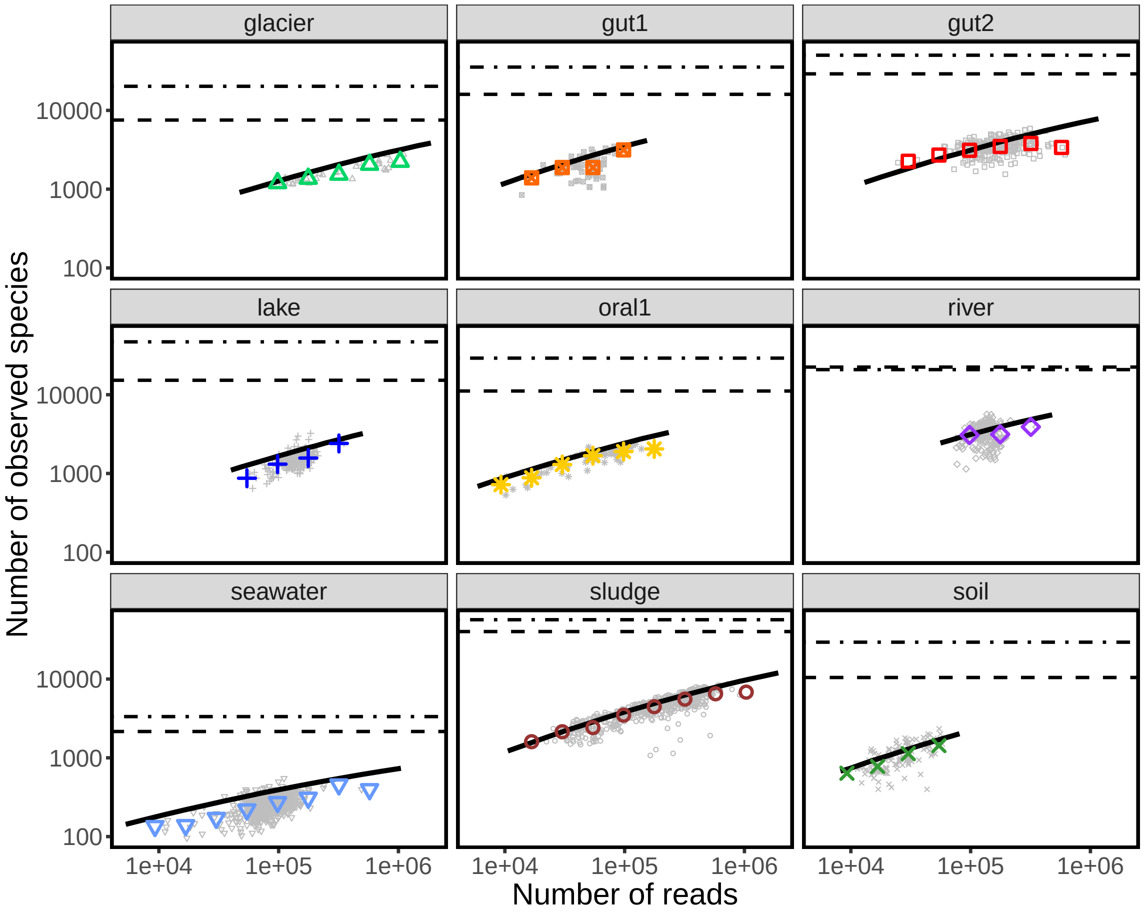

Supplementary Figure 13: **Number of observed species vs. total number of reads.** The number of observed species in a sample/community, depends on the total number of sequences sampled N_s. The more sequences are sampled, the more likely it is to find new species. The gray points report the number of species observed in each community, while the colored symbols are averaged over communities in a range of N_s. The black dashed line represent s_{obs}, the total number of observed species across all the communities in that biome. The black dot-dashed line reports the value of the inferred value of s_{tot}, which includes also the species that have not been observed in that biome. The solid black line is the prediction, reported in equation 59, obtained by combining the three macroecological laws. Equation 59 correctly predicts the typical values of observed number of species, as well as the quantitative relationship between species and number of sequences.

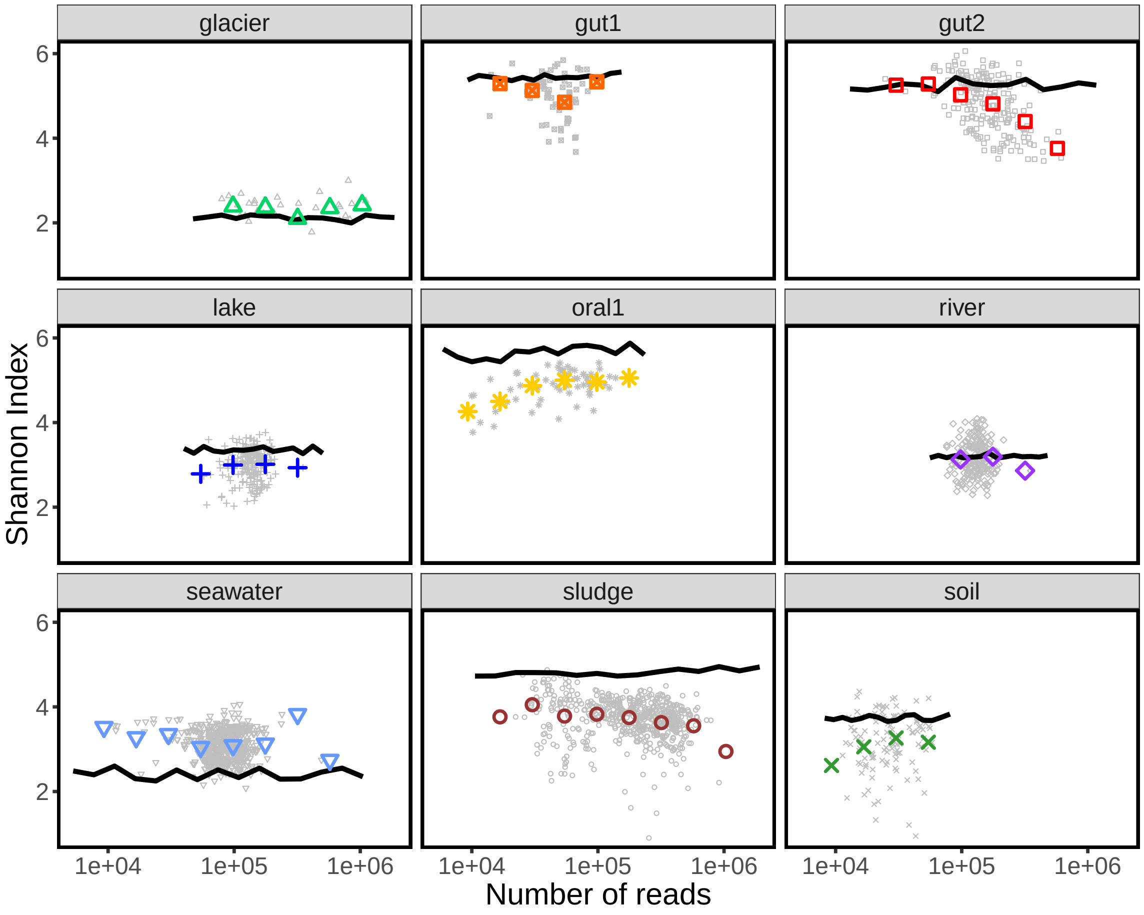

Supplementary Figure 14: **Shannon index vs. total number of reads.** The Shannon index is a measure of diversity which explicitly takes into account the distribution of abundances of species. The Shannon index is defined as the (Shannon) entropy of the probability that a random individual belongs to a given species. The gray points report the number of species observed in each community, while the colored symbols are averaged over communities in a range of number of reads N_s. The solid black line is the prediction, reported in equation 60, obtained by combining the three macroecological laws.

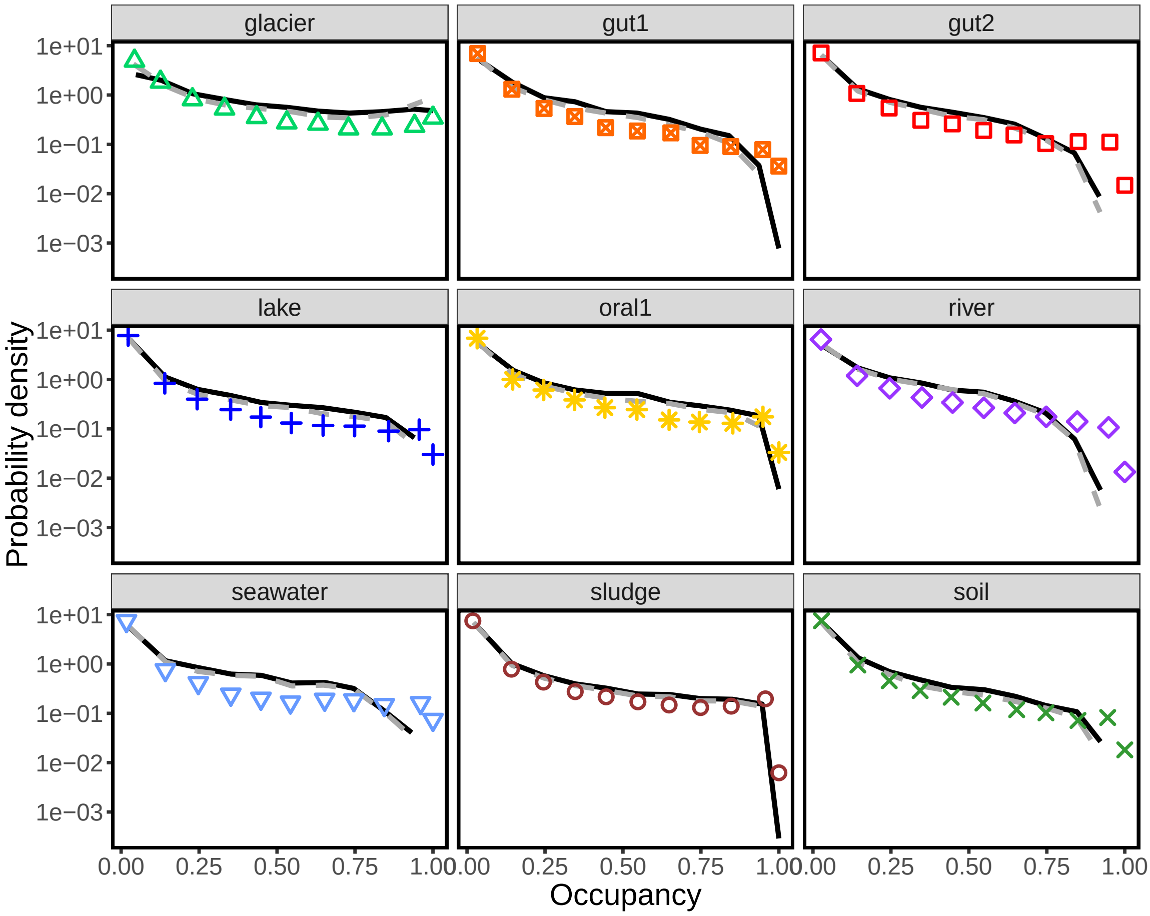

Supplementary Figure 15: **Distribution of occupancy.** The colored symbols report the distribution of species' occupancy in each biome. The solid black line is the prediction, reported equation 63, obtained by combining the three macroecological laws. The gray dashed line is the approximation to the prediction reported in equation 65.

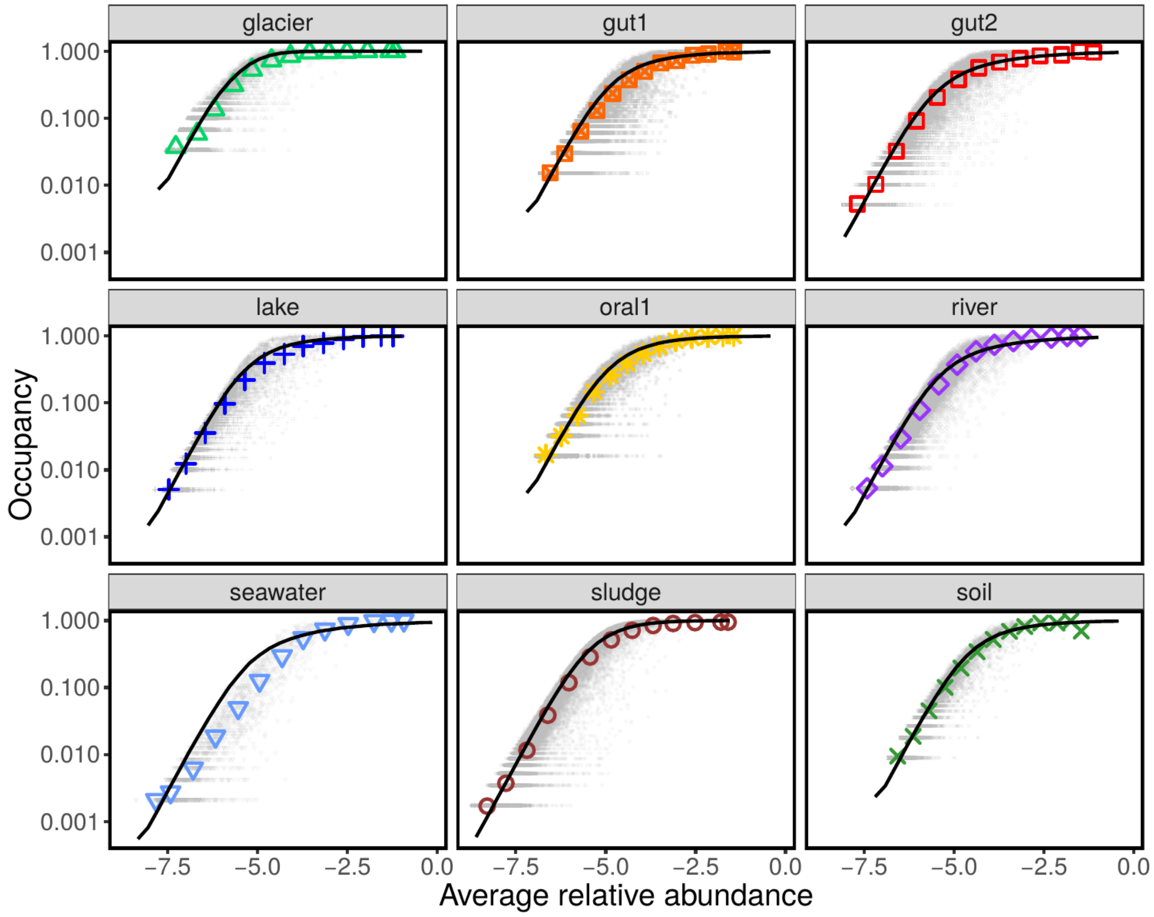

Supplementary Figure 16: **Occupancy-abundance relationship.** The panels report the occupancies o_i vs the log average abundances $\log \bar{x}_i$ of each individual species (gray points). The colored points are averages over species, binned by abundance. The solid black line is the prediction obtained from equation 66.

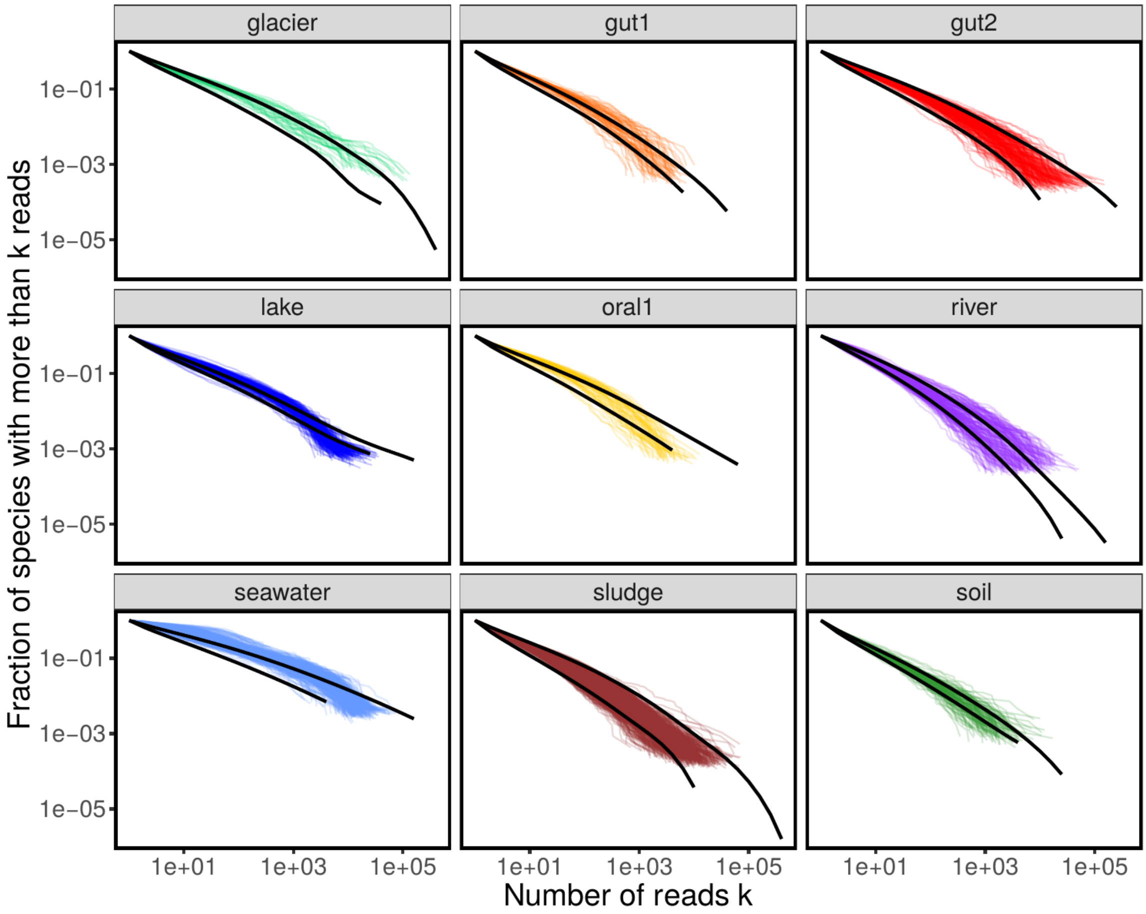

Supplementary Figure 17: **Cumulative Species Abundance Distribution.** The panels report the cumulative Species Abundance Distribution for individual communities (colored lines). The SAD are expected to be influenced by sampling effects, and, in particular, by the total number of reads. The two solid black lines are the expected cumulative SAD for the smallest (on the bottom) and largest (on top) values of total number of reads of each biome.

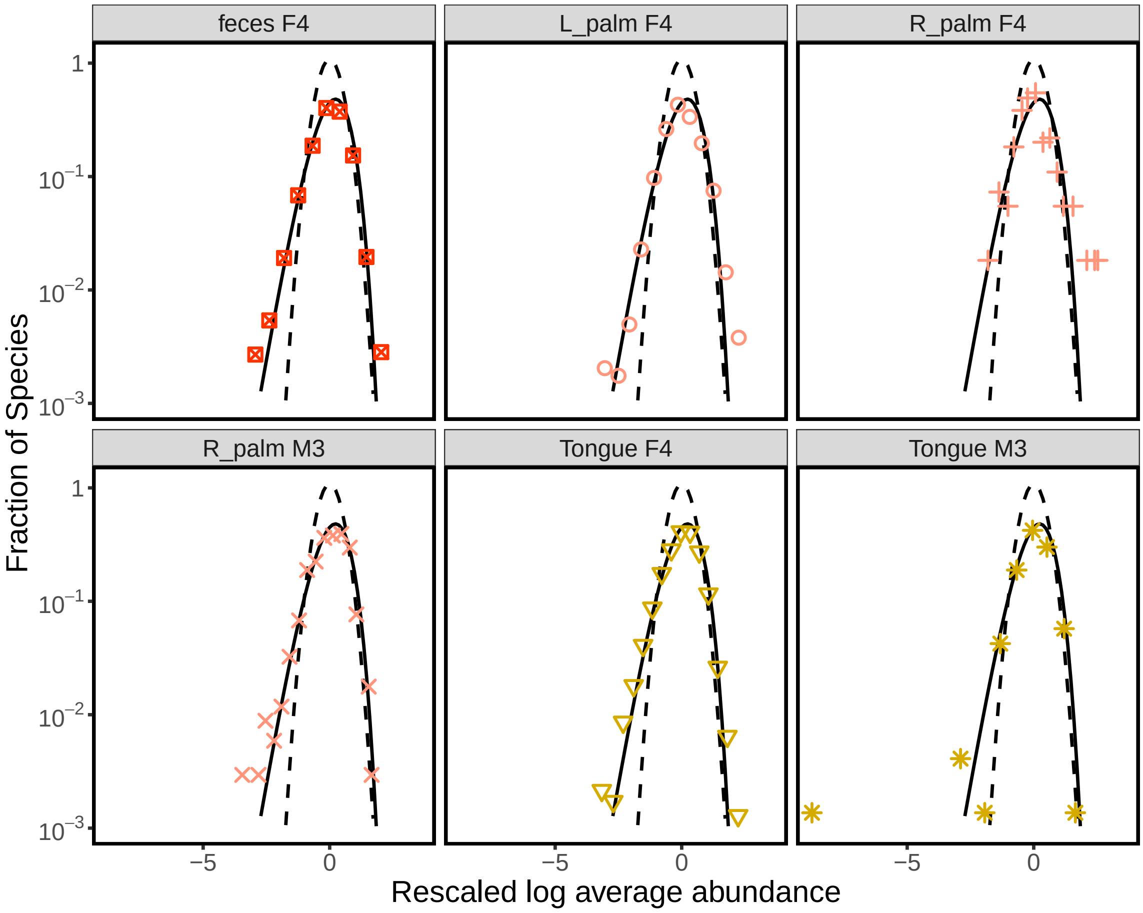

Supplementary Figure 18: **Fluctuations of species abundance over time.** These panels report exactly the same data shown in 4. For each time-series, they were considered only the species present in all the communities. The logarithm of their relative abundances were rescaled (so to have mean zero and unitary variance). The panels report the distribution of these rescaled fluctuations for each biome. Colored points are distributions calculated over both communities and species (same as shown is figure 4). The black continuous line is a Gamma distribution and the black dashed line a Lognormal distribution.

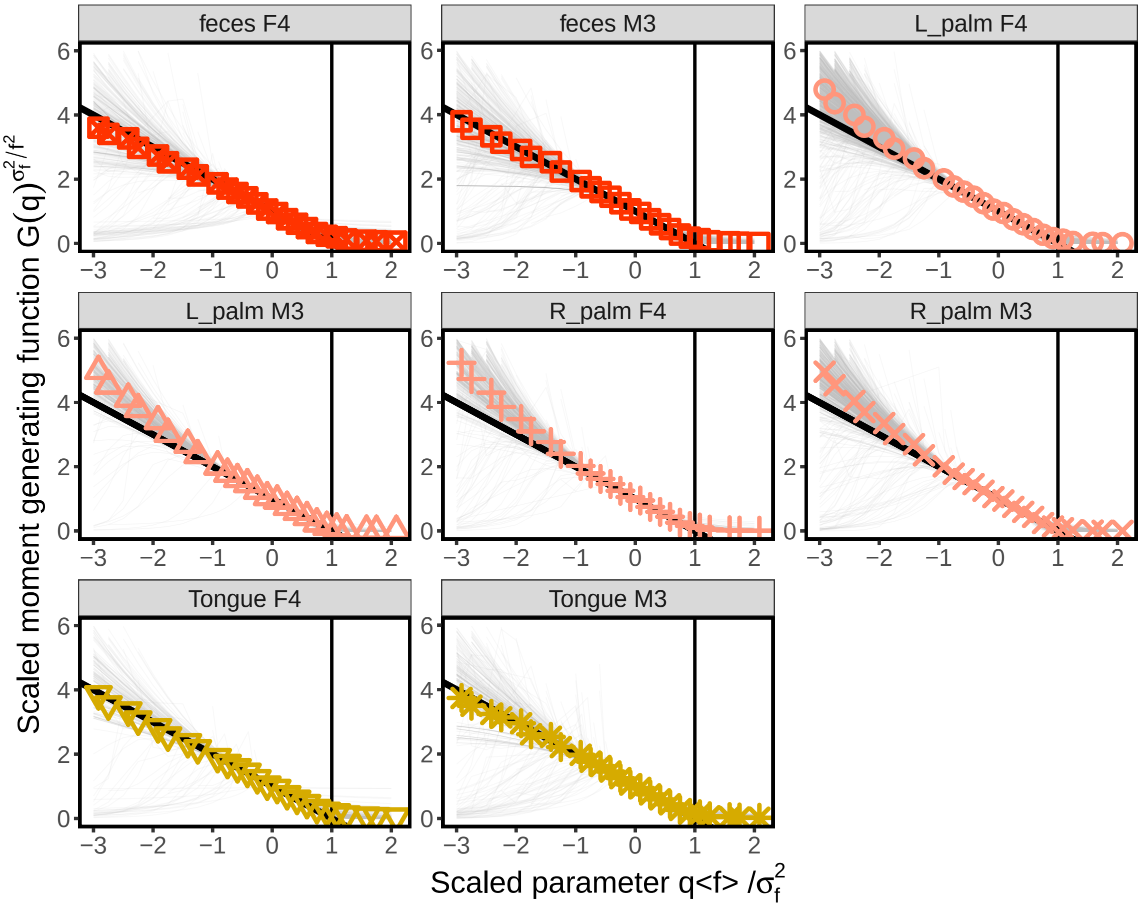

Supplementary Figure 19: **Moment generating function of species fluctuation distribution estimated from time data.** The panels show the moment generating function estimated from the data using equation 33. The gray lines were obtained for individual species (with average abundance $\bar{x}_i > 5 \cdot 10^{-5}$), while colored points are averages over species. The black solid line is the prediction for the Gamma distribution.

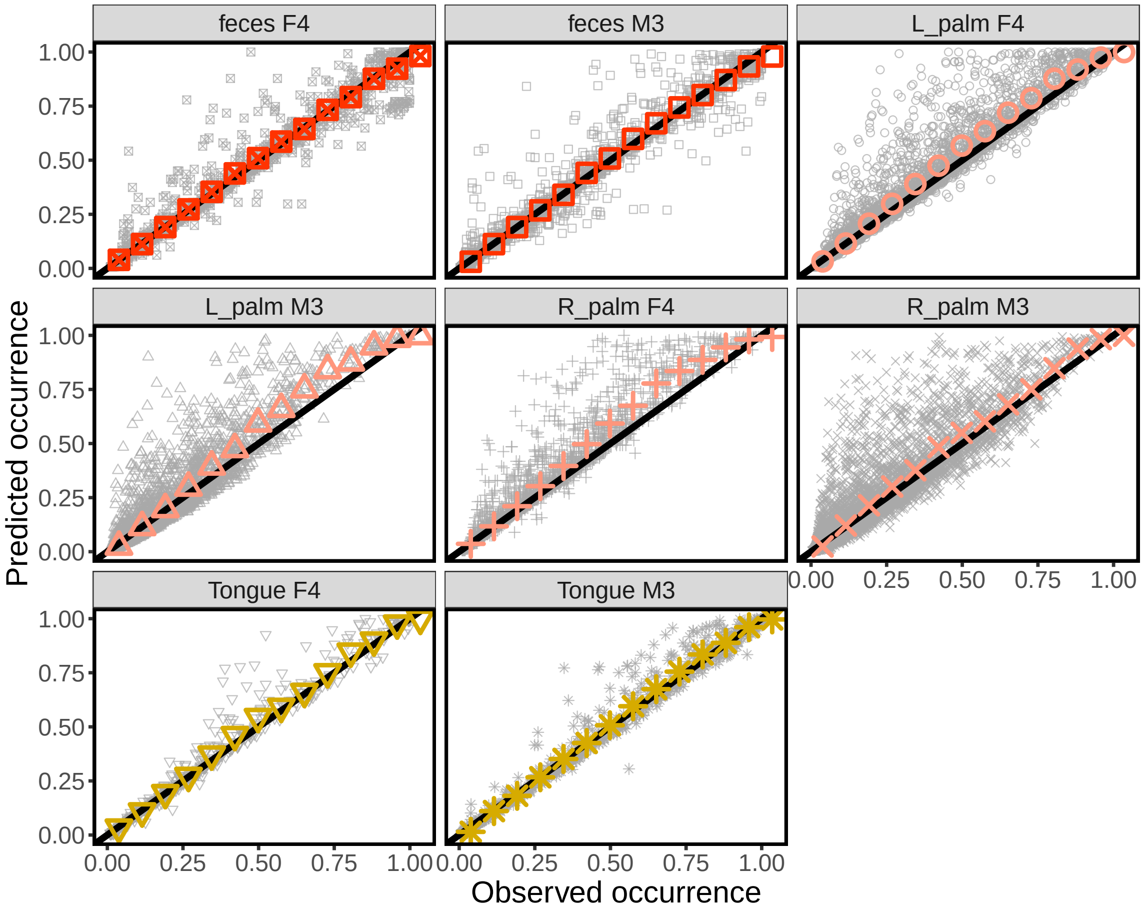

Supplementary Figure 20: **A Gamma AFD correctly predicts species' occupancies for time data.** The occupancy is defined as the fraction of time points where a given species is found to be present. The predicted occupancy was obtained using equation 38, which assumes a Gamma AFD. Using the average and the variance of species' relative abundances, one can in fact estimate the parameters of the AFD and the probability that a species is not found at a given time, given the level of sampling. The black line is the 1 : 1 line, indicating a correct prediction. The gray points are individual species (no filter on average abundance was applied), while the colored points are averages over species.

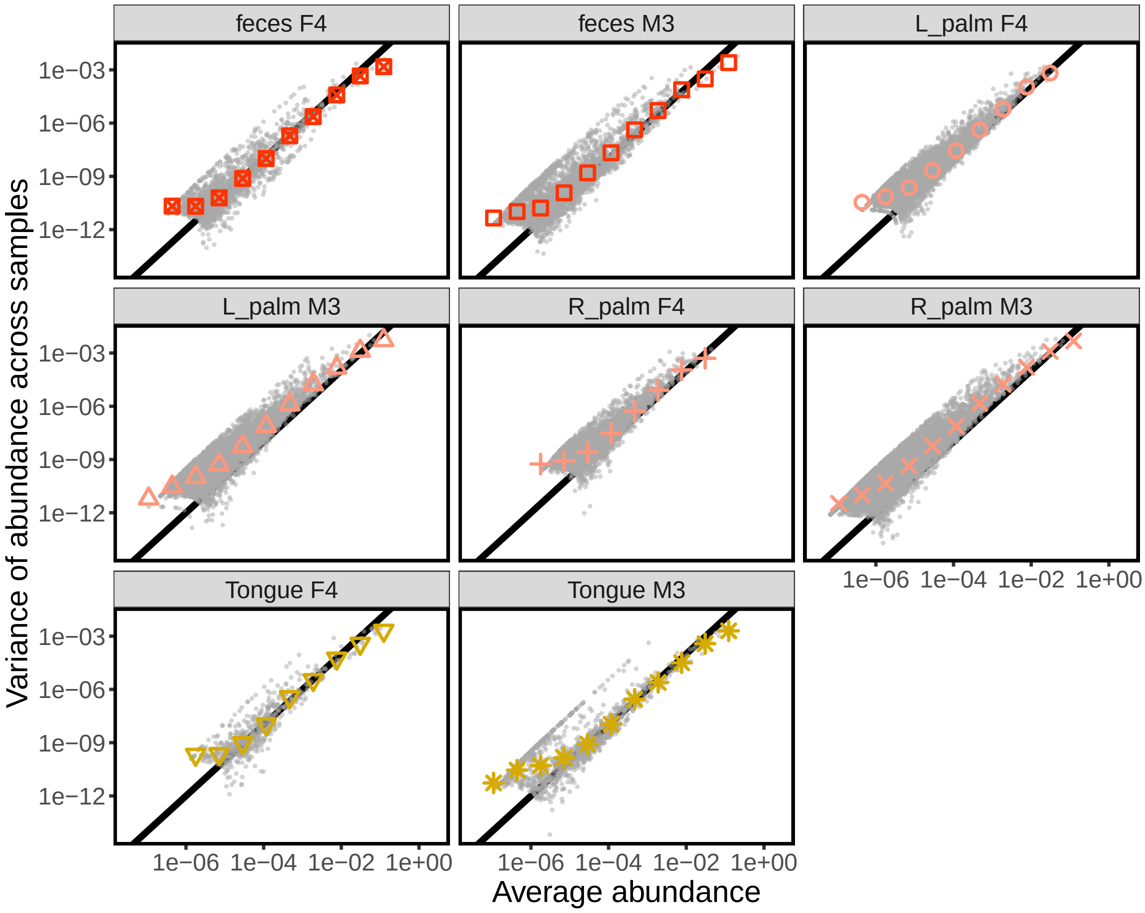

Supplementary Figure 21: **Taylor's law for time data.** The panels show the relationship between mean and variance of abundance. The black line has slope 2, representing the quadratic relationship between variance and mean abundance.

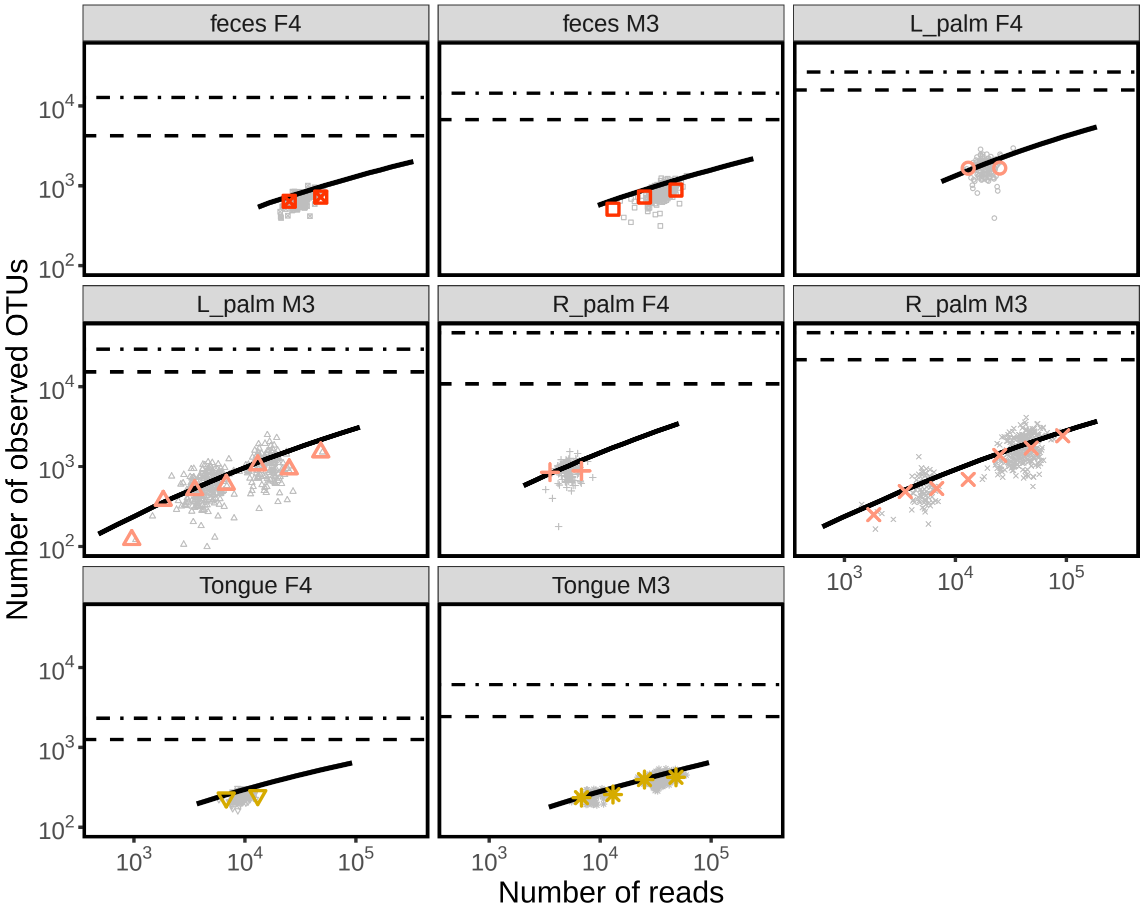

Supplementary Figure 22: **Number of observed species vs. total number of reads for time data.** The number of observed species at a given time, depends on the total number of sequences sampled N_s. The more sequences are sampled, the more likely it is to find new species. The gray points report the number of species observed in each community, while the colored symbols are averaged over times in a range of N_s. The black dashed line represent s_{obs}, the total number of observed species across all the times in that biome. The black dot-dashed line reports the value of the inferred value of s_{tot}, which includes also the species that have not been observed in that biome. The solid black line is the prediction, reported in equation 59, obtained by combining the three macroecological laws. Equation 59 correctly predicts the typical values of observed number of species, as well as the quantitative relationship between species and number of sequences.

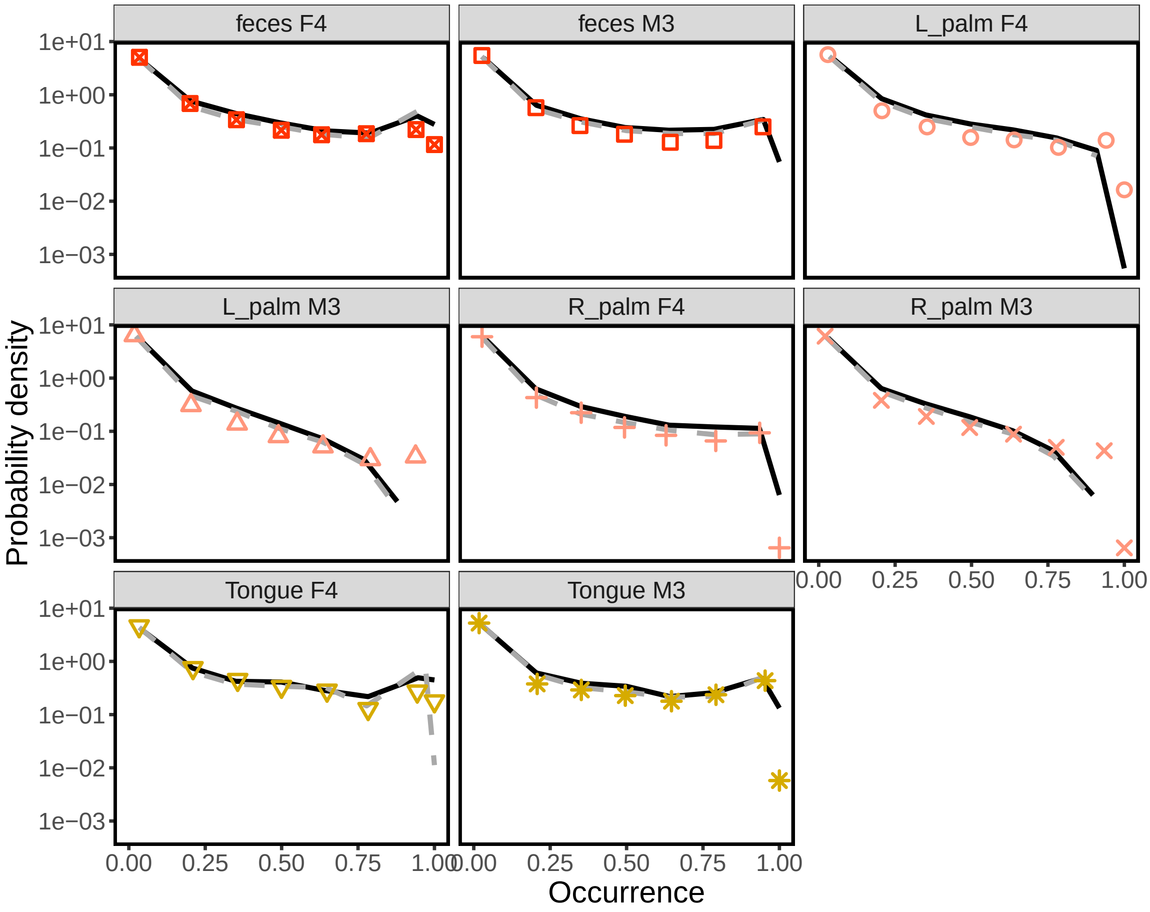

Supplementary Figure 23: **Distribution of occupancy of species across times.** The colored symbols report the distribution of species' occupancies (the fraction of times when a species has been observed) in each biome. The solid black line is the prediction, reported equation 63, obtained by combining the three macroecological laws. The gray dashed line is the approximation to the prediction reported in equation 65.

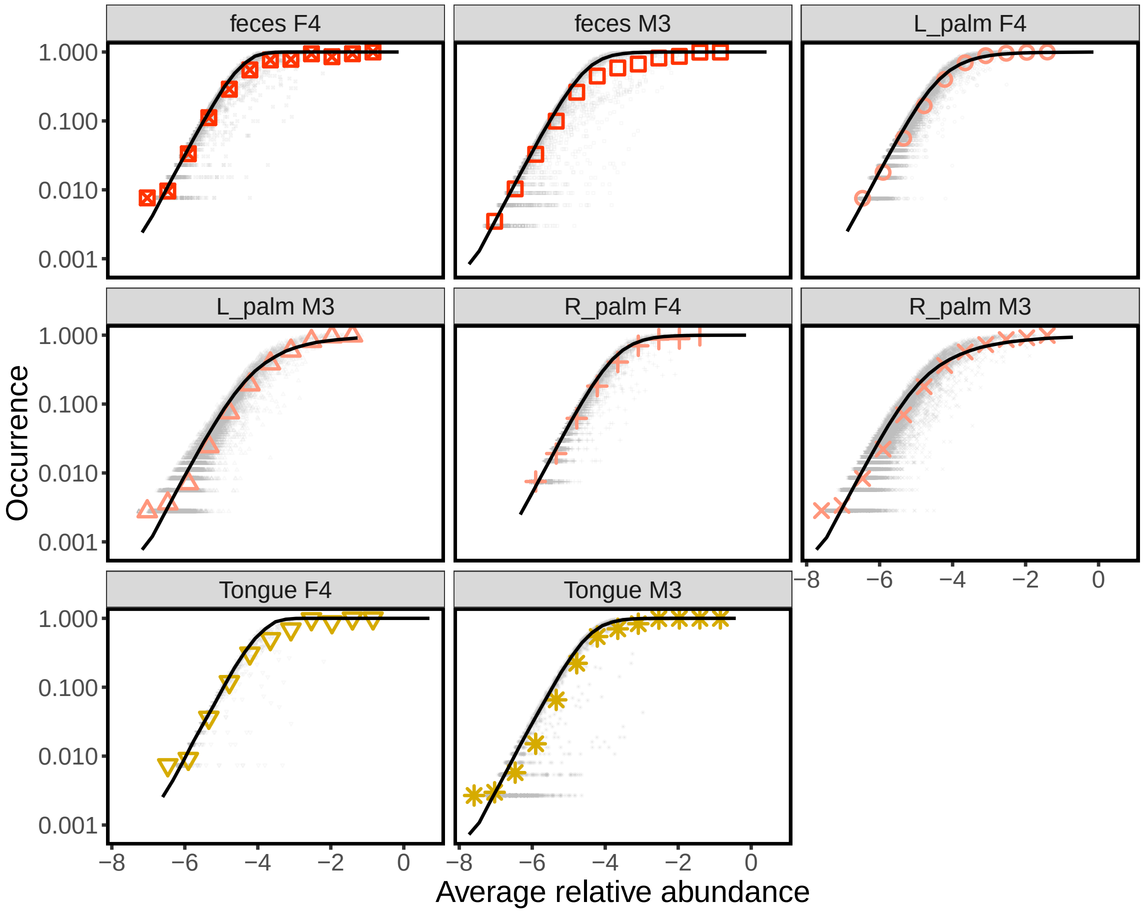

Supplementary Figure 24: **Occupancy-abundance relationship for time data.** The panels report the occupancies o_i vs the log average abundances $\log \bar{x}_i$ of each individual species (gray points). The colored points are averages over species, binned by abundance. The solid black line is the prediction obtained from equation 66.

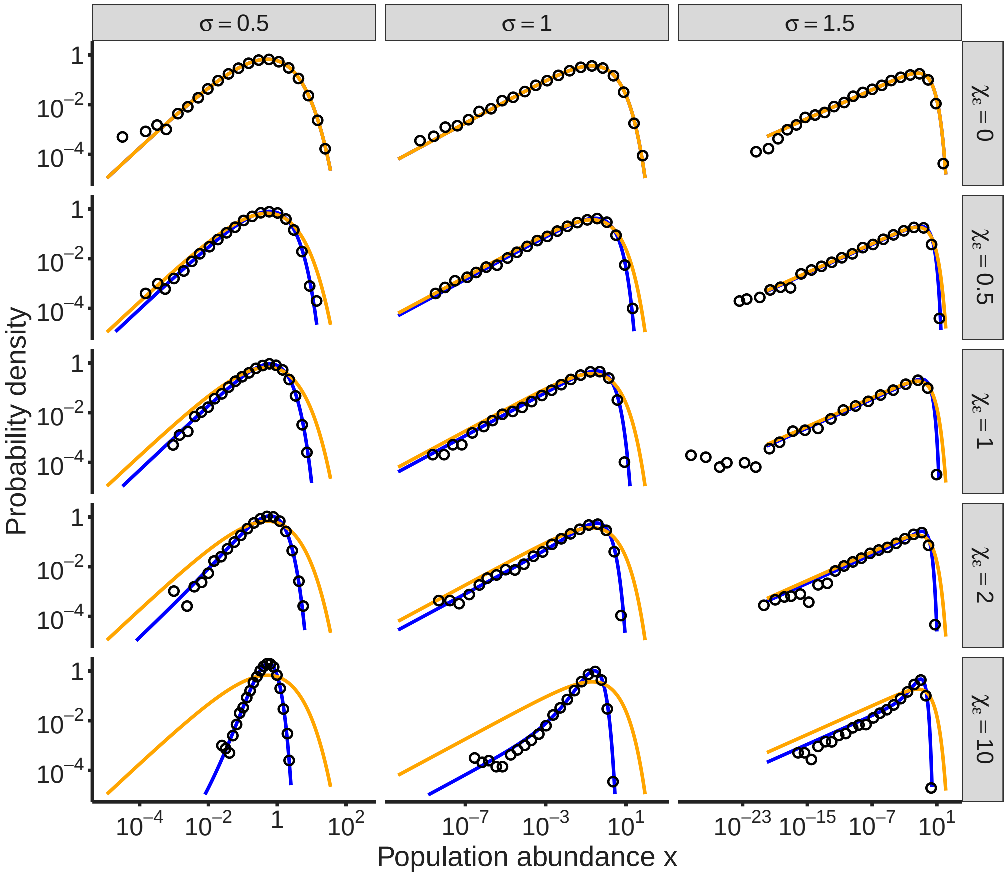

Supplementary Figure 25: **Stochastic logistic model with colored environmental noise.** Black points are numerical simulation of the model described in section 13 for different values of noise strenght σ (columns) and ratio between environmental timescale and population dynamics timescale χ_ϵ (rows). The orange lines are the stationary distributions for white noise ($\chi_\epsilon = 0$), while blue lines are the stationary distribution for arbitrary values of χ_ϵ obtained using the unified colored noise approximation (see equation 86).

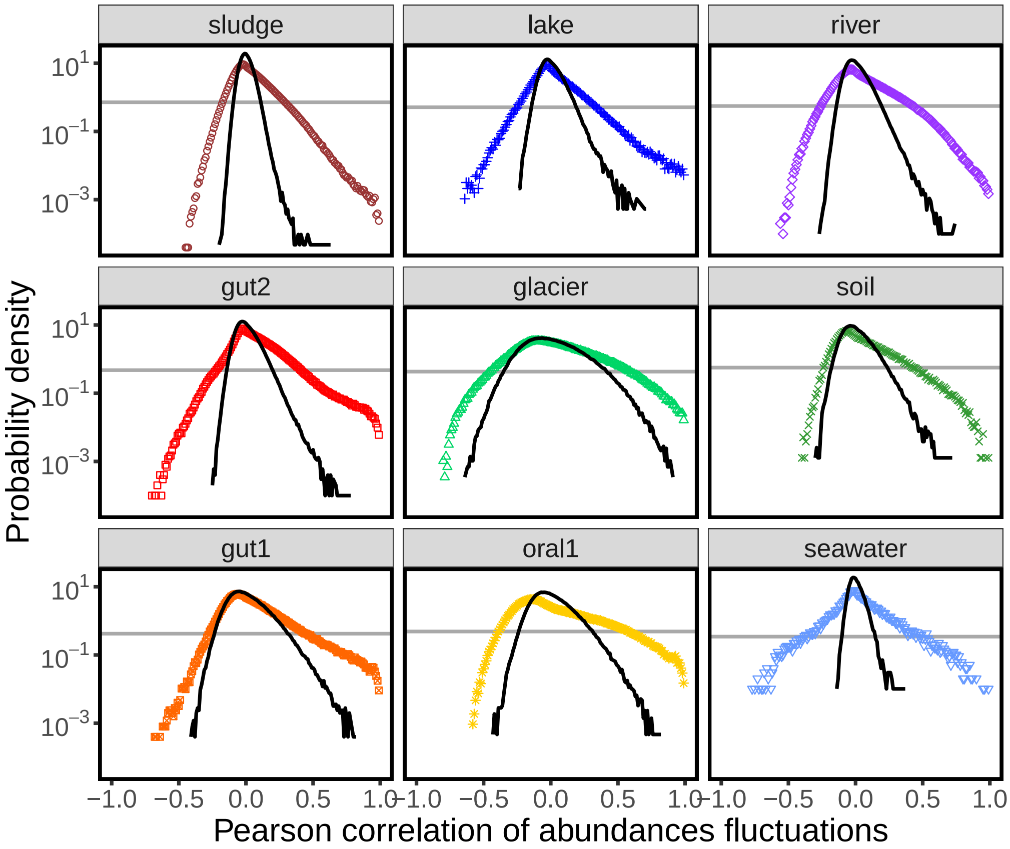

Supplementary Figure 26: **Distribution of Pearson correlation coefficients of abundances fluctuations.** For each biome and each pair of species (present in ar least 50% of the samples), we computed the Pearson correlation of their abundances fluctuations. Each panel shows the distribution of these correlation coefficients (over all the species pairs) for each biome. The colored points are data, while the black line is the null expectation obtained using the first macroecological law, where we empirically fixed the free parameters (empirical mean and variance of each species). They gray horizontal line represent the 95% threshold. These figures convey two important messages: 1) there exist significant correlation which are not captured by the the three macroecological laws alone; 2) correlations are present, but they are weak/sparse: most of pairs of species do not have large correlations.

Macroecological laws describe variation and diversity in microbial communities — Supplementary Materials—

1. DATA

All the datasets analyzed in this work were obtained from EBI Metagenomics [6] (now Magnify) and have been previously published. Raw data were processed under different version of EBI Metagenomics pipelines [6]. The consistency of results across studies and pipelines strongly support the generality of the conclusions. Supplementary Table 1 reports reference to the original works, Magnify pipeline and other information about each dataset. Note that the pipeline version 4.1 uses SILVA [7] to assign OTU classification, while previous versions of the pipeline use QIIME [8] and Greengenes [9]. In the following I will use the word "species" to refer to OTUs, defined accordingly to the methods referred above. Datasets were selected to represent a wide set of biomes. I considered only datasets with at least 50 samples with more than 10^4 reads. No dataset was excluded a-posteriori.

2. SAMPLING

A. Notation

Let N_s be the total number of reads in a biological sample s, and n_i^s the number of reads belonging to species (or any other taxonomic classification) i. By definition $\sum_i n_i^s = N_s$ (if not all the reads are assigned to a species, one can introduce an unassigned category, such that $n_\emptyset^s$ is the number of unassigned reads).

I assume that the set of reads $\{n_\cdot^s\}$ is sample s is produced by a (biased) sampling process. The probability $P_s(\{n.\}|N_s)$ of observing a given set of reads $\{n.\}$ conditioned to a total number of reads N_s is multinomially distributed, i.e.,

$$P_s(\{n.\}|N_s) = \frac{N_s!}{\prod_i n_i!} \prod_i (p_i^s)^{n_i} \ , \tag{1}$$

where p_i^s is the actual frequency of species i in sample s.

We are interested in the variability of abundance across samples. This variability can be recapitulated as fluctuations of the frequencies ps across samples, which are described by some (unknown) probability distribution $\rho(\{p.\})$. In this way, one can write equation 1 as

$$P(\{n.\}|N) = \int [dp] \ \rho(\{p.\}) \frac{N!}{\prod_i n_i!} \prod_i (p_i)^{n_i} \ . \tag{2}$$

I dropped the subscript s of N for simplicity. This equation disentangles two important sources of variability: the variability across samples/communities (described by $\rho(\{p.\})$) and the variability/noise due to sampling (described by the multinomial sampling). Our goal is to study the properties of $\rho(\{p.\})$, i.e. the fluctuations of the ps, how they are distributed, and how they are correlated.

By marginalizing equation 2 one obtains, without any additional assumption (e.g., independence), that the probability of observing n reads of the OTU i equals

$$P_i(n|N) = \int dp \ \rho_i(p) \binom{N}{n} p^n (1-p)^{N-n} \ , \tag{3}$$

where

$$\rho_i(p) = \int [dp] \;\; \rho(\{p.\})\delta(p_i - p) \; , \tag{4}$$

where $\delta(\cdot)$ is the Dirac's delta function.

From equation 2, it is easy to obtain

$$\langle n_i \rangle_N := \sum_{\{n\}} P(\{n\}|N) n_i = \int [dp] \; \rho(\{p.\}) N p_i = N \langle p_i \rangle \; . \tag{5}$$

Since the sampling effort (total number of reads) N_s varies across samples, one cannot directly evaluate $\langle n_i \rangle_N$ from the data. On the other hand one can remove the effect of N by computing $\langle n_i \rangle_N / N$. One can therefore easily estimate $\langle p_i \rangle$

$$\langle p_i \rangle \approx \frac{1}{T} \sum_{s=1}^{T} \frac{n_i^s}{N_s} \; , \tag{6}$$

where T is the number of samples. Applying the same concept to the second moment, one obtains

$$\langle n_i^2 \rangle_N = \int [dp] \; \rho(\{p.\})(N p_i (1 - p_i) + N^2 p_i^2) = N \langle p_i \rangle + N(N-1) \langle p_i^2 \rangle \; . \tag{7}$$

One has therefore that

$$\frac{\langle n_i^2 \rangle_N - \langle n_i \rangle_N}{N(N-1)} = \langle p_i^2 \rangle \; , \tag{8}$$

from which one can estimate

$$\langle p_i^2 \rangle \approx \frac{1}{T} \sum_{s} \frac{n_i^s (n_i^s - 1)}{N_s (N_s - 1)} \; . \tag{9}$$

Note that, even if $N_s \gg 1$, it is in principle incorrect to evaluate $\langle p_i^2 \rangle$ as the sample mean of $(n_i^s / N_s)^2$. This approximation is justified only if all the $n_i^s \gg 1$.

B. Poisson approximation and compositional data

Sequencing data are compositional [10] and therefore their fluctuations are always relative: the equivalence $\sum_i p_i = 1$ constraints the fluctuations of species. If the abundance of a species increases, the abundance of all the other species has to decrease on average. This constraint is explicit in equation 1, or, equivalently in the probability of observing n_i individuals of species i given a relative abundance p_i

$$P(n_i | N, p_i) = \binom{N}{n_i} p_i^n (1 - p_i)^{N-n} \; . \tag{10}$$

At this point, it is useful to consider that one is typically interested in the case where the number of reads is large, i.e. $N_s \gg 1$ (I considered only samples where $N_s > 10^4$, see table 1). Moreover, the most abundant species are a typically small fraction of the samples (i.e., $p_i \ll 1$). In this regime, I can approximate the Binomial distribution which appears in equation 10 with a Poisson distribution, obtaining

$$P_i(n|N) = \int dx \; \rho_i(x) \frac{(xN)^n}{n!} e^{-xN} \; , \tag{11}$$

which correspond to equation 3. The variable x has the same interpretation as p: the relative abundance of a given species.

In principle the constraint $\sum_i x_i = 1$ still holds. The constraint can be relaxed to a milder condition, when it holds on average and $\sum_i \bar{x}_i = 1$. The joint distribution $\rho(\{x.\})$ can be written as

$$\rho(\{x.\}) = \frac{1}{Z}\tilde{\rho}(\{x.\})\delta\left(\sum_{i=1}^{s} x_i - 1\right) \ , \tag{12}$$

where s is the number of species and $\tilde{\rho}(\{x.\})$ is a distribution without the constraint $\sum_i x_i = 1$ and Z is a normalization factor. The moment generating function is defined as $G(h.) = \int [dx] \ \rho(\{x.\}) \exp(i \sum_{j=1}^{s} h_j x_j)$. Using the integral representation of the delta function $\int dk \ \ \exp(ikx) = \delta(x)$, one obtains

$$G(\{h.\}) = \frac{1}{Z}\int [dx] \ \rho(\{x.\}) \exp(i\sum_{j=1}^{s} h_j x_j) = \frac{1}{Z}\int dk \ \int [dx] \ \tilde{\rho}(\{x.\}) e^{-ik + i\sum_{j=1}^{s}(h_j+k)x_j} = \frac{1}{Z}\int dk \ e^{-ik}\tilde{G}(\{h. + k\}) \ , \tag{13}$$

where $\tilde{G}(\{h.\}) = \int [dx] \ \tilde{\rho}(\{x.\}) \exp(i \sum_{j=1}^{s} h_j x_j)$. By writing the Taylor series of $\tilde{G}(\{h. + k\})$ around $k = 0$, I obtain

$$\begin{aligned} G(\{h.\}) = \frac{1}{Z}\int dk \ e^{-ik} \sum_{N=0}^{\infty} \sum_{\{n_1,n_2,\ldots,n_s\}} \delta_{\sum_i n_i, N} \frac{1}{N!} \frac{\partial^N G(\tilde{\{h.\}})}{\partial^{n_1} h_1 \partial^{n_2} h_2 \cdots \partial^{n_s} h_s} k^N = \\ = \frac{1}{Z}\tilde{G}(\{h.\}) \int dk \ e^{-ik} \sum_{N=0}^{\infty} \sum_{\{n_1,n_2,\ldots,n_s\}} \delta_{\sum_i n_i, N} \frac{1}{N!} \langle \tilde{x}_1^{n_1} \tilde{x}_2^{n_2} \ldots \tilde{x}_s^{n_s} \rangle (ik)^N = \\ = \frac{1}{Z}\tilde{G}(\{h.\}) \int dk \ e^{-ik} \tilde{G}_S(k) \ , \end{aligned} \tag{14}$$

where a factor 2π coming from the integral representation of the Delta function was absorbed in Z. The function $\tilde{G}_S(k)$ is equal to

$$\tilde{G}_S(k) = \int [dx] \ \tilde{\rho}(\{x.\}) \exp\left(ik\sum_i x_i\right) \ . \tag{15}$$

Using standard properties of the moment generating function, one has that

$$\tilde{G}_S(k) = \exp(ik \sum_{k=1}^{\infty} c_k) \ , \tag{16}$$

where c_k is the kth cumulant of $\sum_i x_i$. For instance c_1 is the average of $\sum_i x_i$, c_2 is the variance and so on. These cumulants are calculated using the distribution $\tilde{\rho}(\{x.\})$ (which does not have constraints on the sum of the variables). If all the variables x_i were independent, it is easy to show that the k-th cumulant of $\sum_{i=1}^{s} x_i$ scales with the total number of species s as $c_k \sim s^{1-k}$. This scaling relationship is obtained assuming that all the cumulants of sx_i are finite in the limit $s \to \infty$. In this case the leading term is therefore given by $k = 1$, and therefore, for large s one obtains

$$\tilde{G}_S(k) \approx \exp(ikc_1) = \exp(ik \sum_{i=1}^{s} \bar{x}_i) \ , \tag{17}$$

where

$$\bar{x}_i = \int [dx] \ \tilde{\rho}(\{x.\}) \ x_i \ , \tag{18}$$

,

By inserting this expression in equation 14, one obtains

$$G(\{h.\}) \approx \frac{1}{Z}\tilde{G}(\{h.\}) \int dk\; e^{-ik} \exp(ik \sum_{i=1}^{s} \bar{x}_i) = \frac{1}{Z}\tilde{G}(\{h.\})\delta\left(\sum_{i=1}^{s} \bar{x}_i - 1\right) \; . \tag{19}$$

Which translates into

$$\rho(\{x.\}) = \frac{1}{Z}\tilde{\rho}(\{x.\})\delta\left(\sum_{i=1}^{s} \bar{x}_i - 1\right) \; . \tag{20}$$

which constrains the sum of the averages abundances and not on the sum of the random variables. In other words, if the number of species is large and abundances fluctuates independently, the constraints of equation 12 that impose that the sum of the random variables is equal to the unity can be relaxed to a constrain on the sum of the average abundances. If the abundances do not fluctuate dependently is not in principle true that $c_k \sim s^{1-k}$. The first cumulant (the average) is not affected by correlations, while the second cumulant is. In fact

$$c_2 = \langle\left(\sum_i x_i\right)^2\rangle - \langle\left(\sum_i x_i\right)\rangle^2 = \sum_{ij}\langle x_i x_j\rangle - \sum_i \langle x_i\rangle \sum_j \langle x_j\rangle = \sum_i \sigma^2_{x_i} + \sum_{i\neq j}\left(\langle x_i x_j\rangle - \langle x_i\rangle\langle x_j\rangle\right) \; . \tag{21}$$

Since the second cumulants of sx_i is finite, then the variance $\sigma^2_{x_i} \sim s^{-2}$ and therefore $\sum_i \sigma^2_{x_i} \sim s^{-1}$. The second term contains a covariance and is therefore determined by correlation. One can always write the covariance as $\langle x_i x_j\rangle - \langle x_i\rangle\langle x_j\rangle = \rho_{ij}\sigma_{x_i}\sigma_{x_j}$. The product $\sigma_{x_i}\sigma_{x_j}$ scales as s^{-2} and the sum over $i \neq j$ gives a contribution $\sim s^2$. In order to have c_1 dominating over c_2, one need therefore that the typical $\rho \sim s^{-\alpha}$ with $\alpha > 0$. In other words, if the correlations are weak enough (i.e. not every species is correlated with every other species), our approximation still holds for large enough number of species. For instance, if correlations are byproducts of interactions between species and a typical species only interacts with a finite number of species (which does not grow indefinitely as the number of species increases), $\rho \sim s^{-1}$ recovering a scaling $c_2 \sim s^{-1}$. For large number of species, one can expect correlations not to play a role in determining the constraint for species abundance fluctuations.

C. Moment generating function

In equation 7 the second moment of the number of reads of a given OTUs was computed, obtaining a non trivial dependence on the total number of reads N_s. Knowing this dependence allowed to remove the effect of sampling and to estimate the second moment of relative abundance from data obtained under different sampling efforts N_s (eq. 9). It is straightforward to generalize this calculation to the other moments. A more compact and efficient way to achieve the same goal is to estimate the moment generating function. One can calculate

$$\langle z^{n_i}\rangle_N = \sum_{n=0}^{\infty} z^{n_i} P_i(n_i|N) = \int dx_i\; \rho_i(x_i)\exp\left(Nx_i(z-1)\right) = \langle \exp\left(Nx_i(z-1)\right)\rangle \; , \tag{22}$$

where it was used

$$\sum_{n=0}^{N} z^n \frac{(x_i N)^n}{n!} e^{-x_i N} = e^{Nx_i(z-1)} \; . \tag{23}$$

By introducing $q = N(z-1)$, one then obtains

$$\langle\left(1+\frac{q}{N}\right)^{n_i}\rangle_N = \langle\exp\left(x_i q\right)\rangle \ . \tag{24}$$

This suggests that one can remove the effect of the variability in the sampling effort and estimate the moment generating function of $\rho_i(x)$ as

$$G_i(q) = \langle\exp\left(x_i q\right)\rangle \approx \frac{1}{T}\sum_{s=1}^{T}\left(1+\frac{q}{N_s}\right)^{n_i^s} \ , \tag{25}$$

where T is the total number of samples.

3. LAW #1: FLUCTUATIONS OF OTUS ABUNDANCE ACROSS SAMPLES ARE GAMMA DISTRIBUTED

Figure 1 shows that the species that are present in all samples have Gamma distributed fluctuations of abundance, i.e.

$$\rho_i(x) = \frac{1}{\Gamma(\beta_i)}\left(\frac{\beta_i}{\bar{x}_i}\right)^{\beta_i} x^{\beta_i - 1}\exp\left(-\beta_i\frac{x}{\bar{x}_i}\right) \ . \tag{26}$$

Fig. 1 reports only the abundances of species always present. It is in fact not obvious (a-priori) how to treat abundances equal to zero. The instances where species are absent are in fact potentially due to sampling errors, which confound the shape of the AFD at low abundances. Section 4 shows that this in fact happens in the vast majority of cases.

The average relative abundance $\bar{x}_i$ can be simply estimated using equation 6

$$\bar{x}_i \approx \frac{1}{T}\sum_{s=1}^{T}\frac{n_i^s}{N_s} \ . \tag{27}$$

Since the variance of x_i can be estimated as

$$\sigma_{x_i}^2 \approx \frac{1}{T}\sum_s \frac{n_i^s(n_i^s - 1)}{N_s(N_s - 1)} - \left(\frac{1}{T}\sum_{s=1}^{T}\frac{n_i^s}{N_s}\right)^2 \ , \tag{28}$$

and one can write β_i as

$$\beta_i = \left(\frac{\bar{x}_i}{\sigma_{x_i}}\right)^2 \ . \tag{29}$$

The parameter β_i (which is related to the inverse of the coefficient of variation), can be therefore obtained as

$$\beta_i \approx \left(\frac{1}{T}\sum_{s=1}^{T}\frac{n_i^s}{N_s}\right)^2\left(\frac{1}{T}\sum_s \frac{n_i^s(n_i^s - 1)}{N_s(N_s - 1)} - \left(\frac{1}{T}\sum_{s=1}^{T}\frac{n_i^s}{N_s}\right)^2\right)^{-1} \ . \tag{30}$$

The moment generating function of a Gamma distribution is

$$G_i(q) = \langle\exp\left(x_i q\right)\rangle = \left(1 - \frac{\bar{x}_i}{\beta_i}q\right)^{-\beta_i} \ , \tag{31}$$

which can be estimated from data using equation 25. The moment generating function is species dependent, as is depends on $\bar{x}_i$ and β_i. On the other, it is easy to notice that $G_i(q)$ can be collapsed by rescaling both q and G_i:

$$G_i\left(\beta_i\frac{q}{\bar{x}_i}\right)^{-\frac{1}{\beta_i}} = 1 - q \ , \tag{32}$$

which is independent of i. One can therefore test whether x_i is Gamma distributed, by checking that

$$\left(\frac{1}{T}\sum_{s}\left(1+\frac{q}{N_s}\right)^{n_i^s}\right)^{-1/\beta_i} \approx (1-q)\ . \tag{33}$$

Figure 2 shows that the moment generating function estimated from the data using equation 33 is consistent with a Gamma AFD.

4. EXCLUDING COMPETITIVE EXCLUSION

A. Prediction of occupancy from abundance average and variance

The fluctuations of abundance of species i across samples are described by a Gamma distribution

$$\rho_i(x) = \frac{1}{\Gamma(\beta_i)}\left(\frac{\beta_i}{\bar{x}_i}\right)^{\beta_i} x^{\beta_i-1}\exp\left(-\beta_i\frac{x}{\bar{x}_i}\right)\ . \tag{34}$$

The probability of observing n_i reads of OTU i in a sample with N total number of reads is

$$P_i(n_i|N) = \frac{\Gamma(\beta_i+n_i)}{n_i!\Gamma(\beta_i)}\left(\frac{\bar{x}_i N}{\beta_i+\bar{x}_i N}\right)^{n_i}\left(\frac{\beta_i}{\beta_i+\bar{x}_i N}\right)^{\beta_i}\ , \tag{35}$$

and, in particular, the probability of not observing species i is equal to

$$P_i(0|N) = \left(1+\frac{\bar{x}_i N}{\beta_i}\right)^{-\beta_i}\ . \tag{36}$$

The occurrence of species i is defined as the fraction of samples where species i is present, i.e.

$$o_i = \frac{1}{T}\sum_{s=1}^{T}\left(1-\delta_{n_i^s,0}\right) = 1-\frac{1}{T}\sum_{s=1}^{T}\delta_{n_i^s,0}\ , \tag{37}$$

where the Kronecker delta $\delta_{k,0}$ is equal to 1 if $k=0$ and zero otherwise. Using equation 36 one can calculate $\langle o_i\rangle$, the expected occurrence of OTU i, which reads

$$\langle o_i\rangle = 1-\frac{1}{T}\sum_{s=1}^{T}P_i(0|N_s) = 1-\frac{1}{T}\sum_{s=1}^{T}\left(1+\frac{\bar{x}_i N_s}{\beta_i}\right)^{-\beta_i}\ . \tag{38}$$

Note that the two parameters $\bar{x}_i$ and β_i are estimated independently of the occupancy, as they are function of the average relative abundance across samples and its variance only.

Figure 3 shows that a Gamma AFD, using which one can obtain equation 38, correctly predicts empirical species' occupancies. One might wonder how sensitive is this result to the choice of a Gamma AFD. For instance, if the AFD was Lognormal (with parameters m_i and s_i), one would expect

$$P_i(0|N_s) = \int d\eta\ \exp(-e^{\eta})\frac{\exp\left(-\frac{(\eta-m_i)^2}{2s_i^2}\right)}{\sqrt{2\pi s_i^2}}\ , \tag{39}$$

from which one can compute the expected occupancy $\langle o_i\rangle$ as $1-\sum_{s=1}^{T}P_i(0|N_s)/T$. Figure 4 shows that a Lognormal AFD fails in reproducing the occupancies of species, always overestimating occupancies at intermediate values. The observation of a statistical superiority of the Gamma AFD is confirmed by Fig. 5 that shows that the Gamma AFD has a larger maximum likelihood for most of the OTUs across all datasets.

B. Model selection

In this section we compare a purely Gamma AFD with a zero inflated Gamma, which reads

$$\varrho_i(x|\vartheta,\beta,\bar{x}) = \vartheta_i\delta(x) + (1-\vartheta_i)\frac{1}{\Gamma(\beta_i)}\left(\frac{\beta_i}{\bar{x}_i}\right)^{\beta_i} x^{\beta_i-1}\exp\left(-\beta_i\frac{x}{\bar{x}_i}\right) \ , \tag{40}$$

where $\varrho_i(x|\vartheta,\beta,\bar{x})$ is the probability that species i has abundance x, ϑ_i is the probability that a species is truly absent in a community and $\delta(\cdot)$ is the Dirac delta distribution. The assumption behind this model is that if a species is present (which happens with probability $1-\vartheta$) its abundance fluctuations are Gamma distributed. Under this distribution the probability of observing n_i reads for species i in a sample with N total number of reads is

$$P_i(n_i|N) = \vartheta_i\delta_{n_i,0} + (1-\vartheta_i)\frac{\Gamma(\beta_i+n_i)}{n_i!\Gamma(\beta_i)}\left(\frac{\bar{x}_iN}{\beta_i+\bar{x}_iN}\right)^{n_i}\left(\frac{\beta_i}{\beta_i+\bar{x}_iN}\right)^{\beta_i} \ , \tag{41}$$

which reduces to equation 35 when $\vartheta_i = 0$ Note that $P_i(0|N_s)$ is always larger than ϑ_i, as sampling errors are also present here and the probability of false negatives is always nonzero.

Our goal is to test whether the ϑ_is are significantly different from zero. Since the two models tested here are nested, I introduce a prior $\mu(\vartheta)$ over the ϑ and compare the maximum likelihood estimator in the case $\vartheta_i = 0$ with the (maximum) likelihood marginalized over ϑ with prior $\mu(\vartheta)$. Given the number of reads n_i^s of species i in sample s (with N_s) total number of reads, one can compute the ratio

$$\begin{aligned}\ell_i &= \frac{\max_{\bar{x},\beta}\prod_s\int dx\varrho_i(x|0,\beta,\bar{x})\frac{(xN_s)^{n_i^s}}{n_i^s!}e^{-xN_s}}{\int d\vartheta\ \mu(\vartheta)\left(\max_{\bar{x},\beta}\prod_s\int dx\varrho_i(x|\vartheta,\beta,\bar{x})\frac{(xN_s)^{n_i^s}}{n_i^s!}e^{-xN_s}\right)} = \\ &= \frac{\max_{\bar{x},\beta}\prod_s\frac{\Gamma(\beta+n_i^s)}{n_i^s!\Gamma(\beta)}\left(\frac{\bar{x}N_s}{\beta+\bar{x}N_s}\right)^{n_i^s}\left(\frac{\beta}{\beta+\bar{x}N_s}\right)^{\beta}}{\int d\vartheta\ \mu(\vartheta)\left(\max_{\bar{x},\beta}\prod_s\left(\vartheta\delta_{n_i^s,0}+(1-\vartheta)\frac{\Gamma(\beta+n_i^s)}{n_i^s!\Gamma(\beta)}\left(\frac{\bar{x}N_s}{\beta+\bar{x}N_s}\right)^{n_i^s}\left(\frac{\beta}{\beta+\bar{x}N_s}\right)^{\beta}\right)\right)} \ .\end{aligned} \tag{42}$$

If $\ell_i > 1$, the model with $\vartheta_i = 0$ is more strongly supported that the model with $\vartheta \neq 0$. I considered Beta prior

$$\mu(\vartheta) = \frac{\Gamma(a+b)}{\Gamma(a)\Gamma(b)}\vartheta^{a-1}(1-\vartheta)^{b-1} \ , \tag{43}$$

which depends on two hyperparameters a and b. In particular the average ϑ is equal to $a/(a+b)$.

For a given value of ϑ I numerically maximized $\prod_s\int dx\varrho_i(x|\vartheta,\beta,\bar{x})\frac{(xN_s)^{n_i^s}}{n_i^s!}e^{-xN_s}$ over β and $\bar{x}$, by using R non-linear equation solver with Broyden method and multiple initial conditions. By calculating this maximum for $\vartheta = 0$ and comparing it with the averaged value of the maximum over Beta distributed ϑ, I estimated ℓ_i for each species. Figure 6 reports the fraction of species for which $\ell_i < 1$, i.e., the fraction of species for which the inflated gamma is more statistically supported than the standard Gamma, suggesting a ϑ_i significantly different from zero. The value varies across biomes and over choices of a and b, with typical values of 1% to 10% of species displaying $\ell_i < 1$.

Interestingly the fraction of species with $\ell_i < 1$ increases as the average ϑ_i (equal to $a/(a+b)$) decreases. Therefore, the more the prior is concentrated around $\vartheta = 0$, the more likely the inflated Gamma distribution becomes, as it reduces to the standard Gamma.

5. LAW #2: TAYLOR'S LAW FOR ABUNDANCES FLUCTUATIONS

I showed that the fluctuations of OTU abundances across samples are well described by a Gamma distribution (what I called "Law #1"). The parameters of a Gamma distribution are fully specified by its mean and variance. Knowing

the mean and the variance of the relative abundance of a species, is therefore enough to specify the distribution of abundance and descending properties (e.g., the probability of being observed as shown in section 4 A).

In this section, I explore the relation between mean and variance. Figure 7 shows that mean and variance are not independent across species. The variance is, in fact, proportional to the square of the mean. A relation of the type

$$\sigma_x^2 = \langle x \rangle^{2b} \ , \tag{44}$$

is called Taylor's law and has been documented across multiple ecosystems [11]. Figure 7 shows that $b = 1$, which implies that the coefficient of variation is constant. This observations can be translated into constraints on the parameters of the AFD. In fact, since β_i depends only on the coefficient of variation, one can neglect the fluctuations of β_i across species.

6. REPRODUCIBILITY OF AVERAGE ABUNDANCE

The fact that fluctuations of species abundance are well described by a Gamma AFD, reduce variation of each species' abundance to two parameters: mean and variance. Taylor's law, by establishing a link between mean and variance, implies that the average abundance of a species is the most important parameter to characterize its abundance fluctuations. In this section I show that the average summarize non-trivial biological information, being characteristic of a species in a set of similar environments. The average abundance could, in fact, be just a fitted value with little biological significance, not carrying any information about the environment where the species lived in. This could happen in two extreme way: the average is just a value independent on both species identity and biome (like it would be in neutral theory, see section 11) or the average depends on the species only (for instance, if the average abundance was a consequence of some technical choice in the experiment). Figure 8 shows the correlation of species' average abundances across biomes. For each pair of biomes I consider the species present in both biomes and I calculate the correlation between there average relative abundances. Both significant positive and negative correlations are observed. Particularly high are the correlations between two different gut microbiome experiments and between river and lake microbiomes (both freshwater). This results indicates that average abundances are highly reproducible across experimental setups and have significant information about the particular set of environmental conditions.

7. LAW #3: AVERAGE ABUNDANCES ARE LOGNORMALLY DISTRIBUTED

The fluctuations of species abundances across samples are fully specified by the average (relative) abundance. In this section I show that the average abundances are Lognormally distributed across species.

Since we are always dealing with a finite number of (finite) samples, not all the species are observed. We are interested in how the average abundances $\bar{x}_i$ are distributed across species. If a species is rare enough, (i.e., if $\bar{x}_i < c$, where c is a cutoff) it becomes extremely unlikely to observe it. If the "true" distribution $\bar{x}_i$s is described by some probability distribution function $p(\bar{x})$, one expects to observe only the values larger than c

$$p_{emp}(\bar{x}) = \frac{\theta(\bar{x} - c)p(\bar{x})}{\int dz \theta(z - c)p(z)} \ , \tag{45}$$

where c is the cutoff under which species are never observed because they are too rare. Note that this cutoff is a probabilistic one (the probability of observing a species above this cutoff tends to one and is lower than one below).

Figure 9 shows that the distribution of $\bar{x}$ is consistent with a Lognormal distribution. In order to estimate the parameters of the Lognormal, we have to take into account the presence of the cutoff, as defined in equation 45. In other words, if $p(\bar{x})$ is Lognormal, the observed distribution of abundances will be

$$p_{emp}(\bar{x}) = \frac{\sqrt{2}}{\sqrt{\pi\sigma^2}\bar{x}}\theta(\bar{x}-c)\frac{\exp(-\frac{(\log(\bar{x})-\mu)^2}{2\sigma^2})}{\mathrm{erfc}\left(\frac{\log(c)-\mu}{\sqrt{2}\sigma}\right)} \ . \tag{46}$$

The parameters μ and σ are unknown, and should be inferred from data, while c is known (and depends on the number of samples and the sampling effort). The first and second moment of the log average abundances are given by

$$m_1 := \frac{1}{s_{obs}}\sum_i \log(\bar{x}_i) \ , \tag{47}$$

and

$$m_2 := \frac{1}{s_{obs}}\sum_i \left(\log(\bar{x}_i)\right)^2 \ , \tag{48}$$

where s_{obs} is the total number of OTUs observed across samples (with log average abundance larger than the cutoff c). It turns out that the maximum likelihood estimate of μ and σ is the solution of the following system of equations

$$\begin{cases} m_1 = \dfrac{\sqrt{\frac{2}{\pi}}\sigma e^{-\frac{(\log(c)-\mu)^2}{2\sigma^2}}}{\mathrm{erfc}\left(\frac{\log(c)-\mu}{\sqrt{2}\sigma}\right)} + \mu \\ m_2 = \sigma^2 + m_1\mu + cm_1 - \mu c \end{cases} \tag{49}$$

The species below an average log abundance c were not observed. The total number of species s_{tot} can be inferred calculating the probability of observing a species, by assuming that the abundances of non observed species is also lognormally distributed, i.e., to be more precise, that the true distribution of average abundances is

$$p_{true}(\bar{x}) = \frac{1}{\sqrt{2\pi\sigma^2}\bar{x}}\exp\left(-\frac{(\log(\bar{x})-\mu)^2}{2\sigma^2}\right) \ . \tag{50}$$

In this case the expected number of species $\langle s_{obs}\rangle$ with log abundance larger than c is given by

$$\langle s_{obs}\rangle = s_{tot}\int_c^\infty d\bar{x}\ p_{true}(\bar{x}) = \frac{s_{tot}}{2}\,\mathrm{erfc}\left(\frac{\log(c)-\mu}{\sqrt{2}\sigma}\right) \ , \tag{51}$$

where

$$\int_y^\infty dz\ \frac{1}{\sqrt{\pi}}\exp\left(-z^2\right) := \frac{1}{2}\mathrm{erfc}(y) = \frac{1}{2}\left(1-\mathrm{erf}(y)\right) \ . \tag{52}$$

One can infer the total number of species s_{tot} by inverting this equation to obtain

$$s_{tot} = \frac{2\langle s_{obs}\rangle}{\mathrm{erfc}\left(\frac{\log(c)-\mu}{\sqrt{2}\sigma}\right)} \ , \tag{53}$$

and by using the empirical value of s_{obs}. The inferred values of μ, σ and s_{tot} (together with the value of β) are reported in table 2.

Note that the true values of $\bar{x}_i$ are constrained by $\sum_{i=1}^{s_{tot}}\bar{x}_i = 1$, which implies a contraint on the value of parameters. In fact, we have that

$$\frac{1}{s_{tot}} = \frac{1}{s_{tot}}\sum_{i=1}^{s_{tot}}\bar{x}_i = \int_0^\infty d\bar{x}\ p_{true}(\bar{x})\ \bar{x} = \exp\left(\mu+\frac{\sigma^2}{2}\right) \ , \tag{54}$$

which translated in the constraint

$$s_{tot} \exp\left(\mu + \frac{\sigma^2}{2}\right) = 1 \ . \tag{55}$$

I did not impose this constraint in the inference of the parameters, as it leads to very unstable result. For instance, one could use equation 55 to infer s_{tot} from μ and σ. A relatively small error in μ and/or σ would be strongly amplified in the estimate of s_{tot}. I checked numerically (by generating samples from a constrained Lognormal distribution) that estimating independently the three parameters leads to more accurate results in the estimate of μ, σ and s_{tot} at the expenses of imposing the constraint of equation 55 exactly.

8. MACROECOLOGICAL PATTERNS ARE PREDICTED BY LAWS #1, #2 AND #3

Given laws #1, #2, and #3, the probability to observe n reads of a randomly chosen OTUs in a sample with N total reads is

$$P(n|N) = \int d\eta \, \frac{\Gamma(\beta+n)}{n!\Gamma(\beta)} \left(\frac{e^\eta N}{\beta + e^\eta N}\right)^n \left(\frac{\beta}{\beta + e^\eta N}\right)^\beta \frac{\exp\left(-\frac{(\eta-\mu)^2}{2\sigma^2}\right)}{\sqrt{2\pi\sigma^2}} \ , \tag{56}$$

where $\eta = \log(\bar{x})$ and where we are calculating the distribution over all the s_{tot} species (i.e., including the unobserved species).

All the properties of a species are fully specified by its mean abundance $\bar{x} = e^\eta$. The probability of observing n reads of a species with log average abundance η in a sample with N total number of reads is therefore

$$P(n|N,\eta) = \frac{\Gamma(\beta+n)}{n!\Gamma(\beta)} \left(\frac{e^\eta N}{\beta + e^\eta N}\right)^n \left(\frac{\beta}{\beta + e^\eta N}\right)^\beta \ . \tag{57}$$

A. Number of observed species vs total number of reads

The total number of observed species in a sample with N total number of reads can be easily calculated using equation 56. The probability of not observing a species is simply $P(0|N)$. The expected number of distinct OTUs $\langle s(N) \rangle$ in a sample with N reads is therefore

$$\langle s(N) \rangle = s_{tot} \left(1 - P(0|N)\right) = s_{tot} \left(1 - \int d\eta \, \frac{\exp\left(-\frac{(\eta-\mu)^2}{2\sigma^2}\right)}{\sqrt{2\pi\sigma^2}} \left(\frac{\beta}{\beta + e^\eta N}\right)^\beta\right) \ . \tag{58}$$

Figure 13 compares the empirical relation between number of species and total number of reads with the prediction of equation 59.

B. Shannon index

Given a sample s with N_s total number of reads and with n_i^s reads of OTU i, the Shannon diversity index is defined as

$$H_s = -\sum_{i \in s} \frac{n_i^s}{N_s} \log\left(\frac{n_i^s}{N_s}\right) \ . \tag{59}$$

The expected Shannon index is therefore given by

$$\langle H(N)\rangle = -s_{tot}\sum_{n>0}\frac{n}{N}\log\left(\frac{n}{N}\right)P(n|N) =$$
$$= -s_{tot}\int d\eta\,\sum_{n>0}\frac{n}{N}\log\left(\frac{n}{N}\right)\frac{\Gamma(\beta+n)}{n!\Gamma(\beta)}\left(\frac{e^{\eta}N}{\beta+e^{\eta}N}\right)^{n}\left(\frac{\beta}{\beta+e^{\eta}N}\right)^{\beta}\frac{\exp\left(-\frac{(\eta-\mu)^2}{2\sigma^2}\right)}{\sqrt{2\pi\sigma^2}}\ . \tag{60}$$

Figure 14 compares the predictions of equation 60 with data.

C. Occupancy distribution

The occupancy of a species is the fraction of samples where that species is present. The occupancy distribution is the probability distribution of observing a species with a given occupancy. The probability of observing a species with log average abundance η in a sample with N reads is $1-P(0|N,\eta)$, where $P(n|N,\eta)$ is defined in equation 57. Let us define $\chi_s(\eta)$, which is equal to 1 if an OTU with log average abundance η is present in sample s (which happens with probability $1-P(0|N_s,\eta)$) and zero otherwise. The probability $p(o|\eta)$ that a species has occupancy o is given by

$$p(o|\eta) = \sum_{\{\chi_1,\chi_2,\ldots,\chi_s,\ldots,\chi_T\}}\left[\delta\left(o-\frac{1}{T}\sum_s\chi_s\right)\prod_s\left(\delta_{\chi_s,1}\left(1-P(0|N_s,\eta)\right)+\delta_{\chi_s,0}P(0|N_s,\eta)\right)\right]\ . \tag{61}$$

The number of samples oT where a given OTUs is present is therefore given by a Poisson-Binomial distribution, which describes the distribution of the sum of independent non-identically distributed Bernoulli random variables. Using the Fourier transform of the Poisson-Binomial distribution [12], one can write

$$p(o|\eta) = \sum_{t=0}^{T}\delta(o-t/T)\frac{1}{T+1}\sum_{l=0}^{T}e^{-\frac{2\pi i}{T+1}lt}\prod_{s=1}^{T}\left(P(0|N_s,\eta)+e^{\frac{2\pi i}{T+1}l}\left(1-P(0|N_s,\eta)\right)\right)\ . \tag{62}$$

The distribution of occupancy across species $p_{obs}(o)$ can be obtained by averaging 62 over η. Since only the OTUs with $o>0$ can be observed, one has to restrict the summation in eq. 62 only over $t>0$ and to normalize the distribution over the probability of observing a given number of species. The result of this calculation reads

$$p_{obs}(o) = \frac{\int d\eta\,\sum_{t=1}^{T}\delta(o-t/T)\frac{1}{T+1}\sum_{l=0}^{T}e^{-\frac{2\pi i}{T+1}lt}\prod_{s=1}^{T}\left(\left(\frac{\beta}{\beta+e^{\eta}N_s}\right)^{\beta}+e^{\frac{2\pi i}{T+1}l}\left(1-\left(\frac{\beta}{\beta+e^{\eta}N_s}\right)^{\beta}\right)\right)\frac{\exp\left(-\frac{(\eta-\mu)^2}{2\sigma^2}\right)}{\sqrt{2\pi\sigma^2}}}{\int d\eta\,\frac{\exp\left(-\frac{(\eta-\mu)^2}{2\sigma^2}\right)}{\sqrt{2\pi\sigma^2}}\prod_{s=1}^{T}\left(1-\left(\frac{\beta}{\beta+e^{\eta}N_s}\right)^{\beta}\right)}\ . \tag{63}$$

This equation can be simplified by assuming that the occupancy of each species is equal to its mean value, which is justified if the number of samples is large. This assumption corresponds to write

$$p(o|\eta) = \delta(o-\langle t\rangle/T) = \delta(o-\langle o\rangle_\eta) = \delta\left(o-(1-\frac{1}{T}\sum_{s=1}^{T}P(0|N_s,\eta))\right)\ . \tag{64}$$

By averaging over the ηs one obtains the following approximation of equation 63

$$p_{obs}(o) = \frac{\int d\eta\,\delta\left(o-1+\frac{1}{T}\sum_{s=1}^{T}\left(\frac{\beta}{\beta+e^{\eta}N_s}\right)^{\beta}\right)\frac{\exp\left(-\frac{(\eta-\mu)^2}{2\sigma^2}\right)}{\sqrt{2\pi\sigma^2}}}{\int d\eta\,\frac{\exp\left(-\frac{(\eta-\mu)^2}{2\sigma^2}\right)}{\sqrt{2\pi\sigma^2}}\prod_{s=1}^{T}\left(1-\left(\frac{\beta}{\beta+e^{\eta}N_s}\right)^{\beta}\right)} \tag{65}$$

Figure 15 shows the empirical occupancy distributions of different biomes, and it compares them with the prediction of equation 63. Figure 15 also shows that the approximation of equation 65 closely match data and the exact expression of eq. 63.

D. Abundance-occupancy relation

Occupancy (the fraction of samples where a species is found) and average abundance are not independent properties. Given an average (relative) abundance $f = \exp(\eta)$, the expected occupancy is

$$\langle o \rangle_\eta = 1 - \frac{1}{T}\sum_{s=1}^{T} P(0|N_s,\eta) = 1 - \frac{1}{T}\sum_{s=1}^{T}\left(\frac{\beta}{\beta + e^\eta N_s}\right)^\beta \,, \tag{66}$$

where η is equal to the logarithm of the average abundance. Figure 16 shows the empirical occupancy-abundance relationship and its comparison with data.

E. Species Abundance Distribution

One of the most studied patterns in ecology is the Species Abundance Distribution (SAD), which is defined as the number of species with a given abundance. The expected number of species with abundance n in a sample with N total number of reads is given by

$$\langle s_n(N)\rangle = s_{tot} P(n|N) = s_{tot}\int d\eta\, \frac{\Gamma(\beta+n)}{n!\Gamma(\beta)}\left(\frac{e^\eta N}{\beta + e^\eta N}\right)^n \left(\frac{\beta}{\beta + e^\eta N}\right)^\beta \frac{\exp\left(-\frac{(\eta-\mu)^2}{2\sigma^2}\right)}{\sqrt{2\pi\sigma^2}} \,. \tag{67}$$

Consistently to equation 59, the number of observed species $\langle s(N)\rangle$ is given by

$$\langle s(N)\rangle = \sum_{n=1}^{\infty}\langle s_n(N)\rangle = s_{tot}\left(1 - P(0|N)\right) \,. \tag{68}$$

In order to compare different samples, it is often more convenient to study the fraction of species with a given abundance. According to our model, the expected fraction of species with abundance n is

$$\langle \Phi_n(N)\rangle := \frac{\langle s_n(N)\rangle}{\langle s(N)\rangle} = \frac{P(n|N)}{1 - P(0,N)} = \frac{\int d\eta\, \frac{\Gamma(\beta+n)}{n!\Gamma(\beta)}\left(\frac{e^\eta N}{\beta+e^\eta N}\right)^n \left(\frac{\beta}{\beta+e^\eta N}\right)^\beta \frac{\exp\left(-\frac{(\eta-\mu)^2}{2\sigma^2}\right)}{\sqrt{2\pi\sigma^2}}}{1 - \int d\eta\, \left(\frac{\beta}{\beta+e^\eta N}\right)^\beta \frac{\exp\left(-\frac{(\eta-\mu)^2}{2\sigma^2}\right)}{\sqrt{2\pi\sigma^2}}} \,. \tag{69}$$

The cumulative SAD is defined as

$$\langle \Phi_n^{>}(N)\rangle := \sum_{m=n}^{\infty}\langle \Phi_m(N)\rangle = \frac{\int d\eta\, I_{\frac{e^\eta N}{\beta+e^\eta N}}(n,\beta) \frac{\exp\left(-\frac{(\eta-\mu)^2}{2\sigma^2}\right)}{\sqrt{2\pi\sigma^2}}}{1 - \int d\eta\, \left(\frac{\beta}{\beta+e^\eta N}\right)^\beta \frac{\exp\left(-\frac{(\eta-\mu)^2}{2\sigma^2}\right)}{\sqrt{2\pi\sigma^2}}} \,, \tag{70}$$

where $I_p(n,\beta)$ is the regularized incomplete Beta function. Figure 17 compares the empirical cumulative SADs with the prediction of equation 70.

9. MACROECOLOGICAL LAWS IN TEMPORAL DATA

A. Law #1: Fluctuations of OTUs abundance across samples are Gamma distributed

Figure 18 shows the distribution of abundance fluctuations across times for the species with high occurrence. This figure parallels Figure 1, which was obtained using the fluctuations across communities instead of across times. Figure 19 shows the estimated moment generating function, obtained in the same way as of Figure 2.

1. Excluding competitive exclusion in time data

Using the same method explained in section 4 A, I obtained a prediction for the occupancy of each species, based on its average and variance of abundance, which is shown in Figure 20. Also for time data, the presence and absence of species abundances can be predicted from their mean and variance of abundances.

B. Law #2: Taylor's law for abundances fluctuations

Figure 21 shows that the quadratic relationship between mean and variance of abundance also hold for time data. These panels parallel Figure 7 obtained across communities.

C. Law #3: average abundances are Lognormally distributed

Figure 11 shows that the the average abundances are Lognormally distributed also for time data. These panels parallel Figure 9 obtained across communities.

10. PREDICTION OF MACROECOLOGICAL PATTERNS IN TEMPORAL DATA

As reported in section 8 for cross-sectional data, in this section I show that the three macroecological laws are sufficient to predict other commonly studied macroecological patterns also for temporal data.

Figure 22 shows that equation 59 well predicts the observed number of species as a function of the total number of reads. Figure 23 compares data and the prediction of equation 63 for the occupancy distribution, while Figure 24 shows that equation 66 captures the relationship between average abundance and occupancy.

11. MODELS THAT DO NOT REPRODUCE THE OBSERVED PATTERNS

In this section I compare the the three macroecological laws discussed in section 3, 7, and 5 with the predictions of commonly used theory and models. By design, I use three "laws" that were observed across biomes and therefore they are not specific and cannot reveal too many details about ecological mechanisms that act differently in those biomes. This is also the strength of this test. For any model or theory aiming at explaining community composition

and structure at any level of detail, it is a strong requirement to be able to capture the three minimal laws that this work considers.

A. Neutral theory

Neutral theory (NT) [13–15] has been extremely successful in capturing the empirical properties of Species Abundance Distribution of many communities [16–18]. It has also proved to be successful in capturing some temporal and spatial properties [19]. In the context of microbial communities, NT has been tested in different ways [20]. While the existence of significant correlations (as shown in section 14) contradicts NT, the general patterns of abundance and diversity (and in particular the Relative Species Abundance) do not show a strong disagreement with the prediction of NT. By disentangling the shape of the SAD as the result of the combination of a Gamma AFD and a Lognormal MAD, one is instead able to reject NT on the sole basis of patterns of abundance fluctuations. In fact, NT would predict a peaked MAD (Gaussian for a finite number of samples), while a Lognormal distribution in the data is observed. Moreover the average abundance is a conserved property, characteristic of some broad environmental conditions (see section 6).

B. Deterministic models with alternative stable states as source of variation

Lotka-Volterra [21, 22] or consumer-resource models [23] often leads to alternative stable states driven by competitive exclusion: only a subset of the species can coexist in the same community. Which subset of species is realized in a community depends on the initial condition and on its basin of attraction. Within a biome (whose definition is unclear, a-priori) I showed that the presence/absence of species is a consequence of sampling errors. This also applies in a single host when observed over time. While abundance of species fluctuates, the average value around which it fluctuates is conserved over time and across communities. This observations suggest that, as a first approximation, it exists in fact only one basin of attraction (or some globally stable equilibrium) around which species fluctuates. The sentence "as a first approximation" should be interpreted as to characterize the main contributors of fluctuations from community to community, from sample to sample, which are very likely not to be due to alternative stable states.

12. STOCHASTIC LOGISTIC MODEL

I assume that the dynamics of population abundance x_i is defined by

$$\frac{dx_i(t)}{dt} = \frac{x_i(t)}{\tau_i}\left(1 - \frac{x_i(t)}{K_i}\right) + \sqrt{\frac{\sigma_i}{\tau_i}}x_i(t)\xi_i(t)\ , \tag{71}$$

where $\xi_i(t)$ is a Gaussian delta correlated white noise ($\langle\xi_i(t)\rangle = 0$ and $\langle\xi_i(t)\xi_j(t')\rangle = \delta_{ij}\delta(t - t')$).

The term proportional to $\xi_i(t)$ represents environmental fluctuations, which translate into fluctuations of the growth rate and are therefore proportional to x_i. The only essential assumption here is that I assume these fluctuations to occur at a typical timescale that is much shorter than the population dynamics one and to have a finite variance. From this assumption it follows that $\xi_i(t)$ can be well approximated as a Gaussian delta correlated white noise. The

other term is a logistic growth term, with carrying capacity K_i. The parameter τ_i is the timescale of population dynamics and σ_i is measures the coefficient of variation of growth rate fluctuations.

Interpreted with Itô prescription (note however that the corresponding Stranovich equation has the same form up to a redefinition of parameters), equation 81 corresponds to the following Fokker-Planck equation which describes the dynamics of the probability $P_i(x,t)$ of finding species i with abundance x at time t

$$\tau_i \frac{\partial P_i(x,t)}{\partial t} = -\frac{\partial}{\partial x}\left(x\left(1-\frac{x}{K_i}\right)P_i(x,t)\right) + \frac{\sigma_i}{2}\frac{\partial^2}{\partial x^2}\left(x^2 P_i(x,t)\right) \ . \tag{72}$$

The stationary distribution $P_i^*(x) = \lim_{t\to\infty} P(x,t)$ can be found by setting the left hand side of equation 72 equal to zero. Imposing detailed balance, one obtains

$$x\left(1-\frac{x}{K_i}\right)P_i^*(x) = \frac{\sigma_i}{2}\frac{\partial}{\partial x}\left(x^2 P_i^*(x)\right) \ . \tag{73}$$

By introducing $x^2 P_i^*(x) = Q_i(x)$ one obtains

$$\left(\frac{1}{x}-\frac{1}{K_i}\right)Q_i(x) = \frac{\sigma_i}{2}Q_i'(x) \ , \tag{74}$$

which is solved by

$$Q_i(x) = c\exp\left(-\frac{2}{K_i\sigma_i}x\right)x^{2\sigma^{-1}} \ , \tag{75}$$

where c is an arbitrary constant. By using $P_i^*(x) = Q_i(x)x^{-2}$ and fixing c by imposing $\int dx P_i^*(x)$, we obtain

$$P_i^*(x) = \frac{1}{\Gamma(2\sigma_i^{-1}-1)}\left(\frac{2}{K_i\sigma_i}\right)^{2\sigma_i^{-1}-1}\exp\left(-\frac{2}{K_i\sigma_i}x\right)x^{2\sigma_i^{-1}-2} \ , \tag{76}$$

which is a Gamma distribution with mean

$$\langle x_i\rangle = K_i(1-\frac{\sigma_i}{2}) \ , \tag{77}$$

and squared coefficient of variation

$$\frac{\langle x_i^2\rangle - \langle x_i\rangle^2}{\langle x_i\rangle^2} = \frac{\sigma_i}{2-\sigma_i} \ . \tag{78}$$

It is important to observe that the mean abundance is positive and the variance is finite only if $\sigma_i < 2$. If the environmental fluctuations are too strong the population is, in fact, driven to extinction. This effect is driven by the multiplicative nature of the noise in equation 81. By introducing the variable $q_i = \log x_i$ in equation 81, one obtains (under Itô prescription)

$$\frac{dq_i(t)}{dt} = \frac{1}{\tau_i}\left(1-\frac{\sigma_i}{2}-\frac{e^{q_i}}{K_i}\right) + \sqrt{\frac{\sigma_i}{\tau_i}}\xi_i(t) \ . \tag{79}$$

The term $-\frac{\sigma}{2}$ comes from the change of variable following Itô prescription. By taking the average on both sides one obtains

$$\frac{d\langle q_i(t)\rangle}{dt} = \frac{1}{\tau_i}\left(1-\frac{\sigma_i}{2}-\frac{\langle e^{q_i}\rangle}{K_i}\right) \ . \tag{80}$$

Since the term $\langle e^{q_i}\rangle$ is always positive, it is evident that $\frac{d\langle q_i(t)\rangle}{dt}$ reaches a stationary value only if $1-\sigma_i/2 > 0$, i.e. if $\sigma_i < 2$. If $\sigma_i > 2$ the population abundance decreases indefinitely.

13. STOCHASTIC LOGISTIC MODEL WITH COLORED NOISE

The SLM describes the dynamics in presence of environmental noise. In the previous section I considered the case where environmental noise is white (i.e., the autocorrelation time goes to zero). In this section I extend the SLM to include colored environmental noise, with non-zero autocorrelation time τ_ϵ. In order to simplify the notation, and without loss of generality, the index i is removed. I consider the following model

$$\frac{dx(t)}{dt} = \frac{x(t)}{\tau}\left(1 - \frac{x(t)}{K}\right) + \sqrt{\frac{\sigma}{\tau}} x(t)\epsilon(t) \ , \tag{81}$$

where $\epsilon(t)$ is a Gaussian noise with mean zero $\langle \epsilon_i(t) \rangle = 0$ and exponentially decaying autocorrelation

$$\langle \epsilon(t)\epsilon(t') \rangle = \frac{1}{2\tau_\epsilon} \exp\left(-\frac{|t-t'|}{\tau_\epsilon}\right) \ . \tag{82}$$

One can equivalently write an equation to describe the dynamics of the variable $\epsilon(t)$ as

$$\frac{d\epsilon(t)}{dt} = -\frac{1}{\tau_\epsilon}\epsilon(t) + \frac{1}{\tau_\epsilon}\xi(t) \ , \tag{83}$$

where $\xi(t)$ is Gaussian, delta correlated, white noise $\langle \xi(t)\xi(t') \rangle = \delta(t-t')$. One can introduce $\chi_\epsilon = \tau_\epsilon/\tau$, which measures the relative timescale of the environment compared to the population dynamics one. When $\chi_\epsilon \to 0$ one obtains the SLM with white noise. In fact, if $\tau_\epsilon \to 0$ the autocorrelation of the environmental noise in eq 82 tend to a delta function. Less formally, in that limit, one obtains that in equation 83 one can substitute $\epsilon(t) = \xi(t)$ when $\tau_\epsilon \to 0$.

Figure 25 compares the stationary distribution obtained for different values of σ and ξ_ϵ. Interestingly, even for values of $\chi_\epsilon \sim 1$ one obtains a distribution that resembles a Gamma. Only for significantly larger values of χ_ϵ important deviations emerge.

One can obtain an accurate approximation of the stationary distribution for arbitrary values of χ_ϵ using the unified colored-noise approximation [24]. This approximation is exact only in the limits $\chi_\epsilon \to 0$ and $\chi_\epsilon \to \infty$, but it turns out to be accurate also for intermediate values. In order to obtain this approximation, it is convenient to write the stationary distribution for $q = \log(x)$. In the case $\chi_\epsilon = 0$, it can be obtained from 76 and reads

$$P_i^*(q|\chi_\epsilon = 0) = \frac{1}{\Gamma(2\sigma^{-1}-1)}\left(\frac{2}{K\sigma}\right)^{2\sigma^{-1}-1} \exp\left(-\frac{2}{K\sigma}e^q + (2\sigma^{-1}-1)q\right) \ . \tag{84}$$

With a straightforward application of the unified colored-noise approximation [24], one obtains

$$P_i^*(q|\chi_\epsilon) = \frac{1}{Z(\chi_\epsilon)} P_i^*(q|\chi_\epsilon = 0)\left(1 + \chi_\epsilon \frac{e^q}{K}\right) \exp\left(-\frac{\chi_\epsilon}{\sigma}\left(1 - \frac{\sigma}{2} - \frac{e^q}{K}\right)^2\right) \ , \tag{85}$$

where $Z(\chi_\epsilon)$ is a normalization constant. By substituting $x = e^q$ one easily obtains

$$P_i^*(x|\chi_\epsilon) = \frac{1}{Z(\chi_\epsilon)} P_i^*(x|\chi_\epsilon = 0)\left(1 + \chi_\epsilon \frac{x}{K}\right) \exp\left(-\frac{\chi_\epsilon}{\sigma}\left(1 - \frac{\sigma}{2} - \frac{x}{K}\right)^2\right) \ . \tag{86}$$

Figure 25 shows that the analytical approximation of equation 86 accurately matches the numerical simulations.

14. CORRELATIONS OF SPECIES ABUNDANCE FLUCTUATIONS

In the previous sections I showed that the three macroecological laws correctly capture many statistical properties of the empirical data. In this section I show that they do not capture all the statistical properties, and, in particular, they fail in describing the correlations between species abundance fluctuations.

In section 8, in order to reproduce the macroecological patterns starting from the three macroecological laws, I assumed that the abundance fluctuations were independent across species. More generally one can write

$$P_{ij}(n_i, n_j|N) = \int dx\ dy\ \frac{(xN)^{n_i}}{n_i!} e^{-xN}\ \frac{(yN)^{n_i}}{n_i!} e^{-yN}\ \rho_{ij}(x, y)\ , \tag{87}$$

where $P_{ij}(n_i, n_j|N)$ is the probability of observing n_i reads of species i and n_j reads of species j in a sample with N total number of reads, and $\rho_{ij}(x, y)$ is the joint probability distribution of the (relative) abundances. So far I have assumed $\rho_{ij}(x, y) = \rho_i(x)\rho_j(y)$ which implies in turn $P_{ij}(n_i, n_j|N) = P_i(n_i, N)P_j(n_j|N)$. Using the same concepts introduced in section 2, it is easy to show that

$$\int dx\ dy\ xy\ \rho_{ij}(x, y) = \langle x_i x_j \rangle \approx \frac{1}{T} \sum_{s=1}^{T} \frac{n_i^s}{N_s} \frac{n_j^s}{N_s}\ , \tag{88}$$

for a large number of samples T. By considering also mean and variance of the two marginal distributions $\rho_i(x)$ and $\rho_j(y)$ one can easily estimate the Pearson correlation coefficient r_{ij}. In the limit of large number of samples, if the abundances were independent, the Pearson correlation coefficient would tend to zero. Since we are working with a finite number of samples, the fluctuations in the estimated correlation coefficient cannot be neglected. Instead of studying all the correlations independently, I considered the distribution of coefficients r_{ij}, formally defined as

$$q(r) := \frac{2}{s(s-1)} \sum_{i>j} \delta(r - r_{ij})\ , \tag{89}$$

where s is the number of species considered.

Figure 26 compares the empirical distribution of Pearson correlations $q(r)$, with the one obtained by imposing independence between species and using the three macroecological laws. The first important observation is that the two distribution differs: there are in fact significant correlation which cannot be neglected. The second important observation is that the empirical $q(r)$ is centered about zero: most of the species pairs have low / non-significant correlations. This latter observation agrees qualitatively with the null expectation, but is far from trivial. In fact, it implies that correlations are weak (correlation coefficient are small) and sparse (pairs of species with large correlations are rare).

These two important results are retrospectively important to interpret one of the main results of the paper. It is possible to make predictions about the macroecological patterns by assuming only the three macroecological laws and independence between species because correlations are weak, and therefore they do not affect much the macroecological patters (which are averages over species or samples). In turn, the results of the papers are essential to detect correlation, providing an empirically-validated null model.

Supplementary References

---

[1] Ambrosini, R. *et al.* Diversity and Assembling Processes of Bacterial Communities in Cryoconite Holes of a Karakoram Glacier. *Microbial Ecology* **73**, 827–837 (2017).

[2] Li, J. *et al.* Gut microbiota dysbiosis contributes to the development of hypertension. *Microbiome* **5**, 14 (2017).

[3] Niño-García, J. P., Ruiz-González, C. & del Giorgio, P. A. Interactions between hydrology and water chemistry shape bacterioplankton biogeography across boreal freshwater networks. *The ISME Journal* **10**, 1755–1766 (2016).

[4] Easson, C. G. & Lopez, J. V. Depth-Dependent Environmental Drivers of Microbial Plankton Community Structure in the Northern Gulf of Mexico. *Frontiers in Microbiology* **9**, 3175 (2019).

[5] Caporaso, J. G. *et al.* Moving pictures of the human microbiome. *Genome Biology* **12**, R50 (2011).

[6] Mitchell, A. L. *et al.* EBI Metagenomics in 2017: enriching the analysis of microbial communities, from sequence reads to assemblies. *Nucleic Acids Research* **46**, D726–D735 (2018).

[7] Quast, C. *et al.* The SILVA ribosomal RNA gene database project: Improved data processing and web-based tools. *Nucleic Acids Research* **41** (2013).

[8] Bolyen, E. *et al.* Reproducible, interactive, scalable and extensible microbiome data science using QIIME 2. *Nature Biotechnology* **37**, 852–857 (2019).

[9] McDonald, D. *et al.* An improved Greengenes taxonomy with explicit ranks for ecological and evolutionary analyses of bacteria and archaea. *ISME Journal* **6**, 610–618 (2012).

[10] Gloor, G. B., Macklaim, J. M., Pawlowsky-Glahn, V. & Egozcue, J. J. Microbiome Datasets Are Compositional: And This Is Not Optional. *Frontiers in Microbiology* **8**, 2224 (2017).

[11] Taylor, L. R. & Woiwod, I. P. Comparative Synoptic Dynamics. I. Relationships Between Inter- and Intra-Specific Spatial and Temporal Variance/Mean Population Parameters. *The Journal of Animal Ecology* **51**, 879 (1982).

[12] Fernandez, M. & Williams, S. Closed-Form Expression for the Poisson-Binomial Probability Density Function. *IEEE Transactions on Aerospace and Electronic Systems* **46**, 803–817 (2010).

[13] Hubbell, S. P. *The Unified Neutral Theory of Biodiversity and Biogeography* (Princeton University Press).

[14] Rosindell, J., Hubbell, S. P., He, F. L., Harmon, L. J. & Etienne, R. S. The case for ecological neutral theory. *Trends in Ecology \& Evolution* **27**, 203–208 (2012).

[15] Azaele, S. *et al.* Statistical mechanics of ecological systems: Neutral theory and beyond. *Reviews of Modern Physics* **88**, 035003 (2016).

[16] Volkov, I., Banavar, J. R., Hubbell, S. P. & Maritan, A. Neutral theory and relative species abundance in ecology. *Nature* **424**, 1035–7 (2003).

[17] Volkov, I., Banavar, J. R., He, F., Hubbell, S. P. & Maritan, A. Density dependence explains tree species abundance and diversity in tropical forests. *Nature* **438**, 658–61 (2005).

[18] Volkov, I., Banavar, J. R., Hubbell, S. P. & Maritan, A. Patterns of relative species abundance in rainforests and coral reefs. *Nature* **450**, 45–9 (2007).

[19] Azaele, S., Pigolotti, S., Banavar, J. R. & Maritan, A. Dynamical evolution of ecosystems. *Nature* **444**, 926–8 (2006).

[20] Bertuzzo, E. *et al.* Spatial effects on species persistence and implications for biodiversity. *Proceedings of the National Academy of Sciences of the United States of America* **108**, 4346–51 (2011).

[21] Goh, B. & Jennings, L. Feasibility and stability in randomly assembled Lotka-Volterra models. *Ecological Modelling* **3**, 63–71 (1977).

[22] Biroli, G., Bunin, G. & Cammarota, C. Marginally stable equilibria in critical ecosystems. *New Journal of Physics* **20** (2018). 1710.03606.

[23] Marsland, R. *et al.* Available energy fluxes drive a transition in the diversity, stability, and functional structure of microbial communities. *PLoS Computational Biology* **15** (2019). 1805.12516.

[24] Jung, P. & Hänggi, P. Dynamical systems: A unified colored-noise approximation. *Physical Review A* **35**, 4464–4466 (1987).
